# Efficient synthesis of new indenopyridotriazine [4.3.3]propellanes and spiroindenopyridotriazine-4*H*-pyran derivatives†

**DOI:** 10.1039/d3ra06248a

**Published:** 2023-10-27

**Authors:** Monireh Rezaei, Mohammad Bayat

**Affiliations:** a Department of Chemistry, Faculty of Science, Imam Khomeini International University Qazvin Iran bayat_mo@yahoo.com m.bayat@sci.ikiu.ac.ir

## Abstract

The pyrido[1,2,4]triazines as substrates, generated from 1,6-diaminopyridinone derivatives and ninhydrin, were reacted with malononitrile and CH-acids to afford a new library spiro[indeno[1,2-*e*]pyrido[1,2-*b*][1,2,4]triazine-7,5′-pyran]-1,3,6′-tricarbonitrile in ethanol at reflux condition in excellent yield. Also, novel indenopyridotriazine [4.3.3]propellanes were synthesized *via* the reaction of pyrido[1,2,4]triazine and *N*-methyl-1-(methylthio)-2-nitroethenamine (NMSM) by using of HOAc in ethanol. The important aspects of this protocol are the abundance of starting materials, mild conditions, structural diversity of products, excellent yields and easy isolation of products with no chromatographic technique.

## Abstract 2

Efficient synthesis of indenopyridotriazine [4.3.3]propellanes and spiroindenopyran derivatives using a sequential one-pot four component reaction.

## Graphical abstract

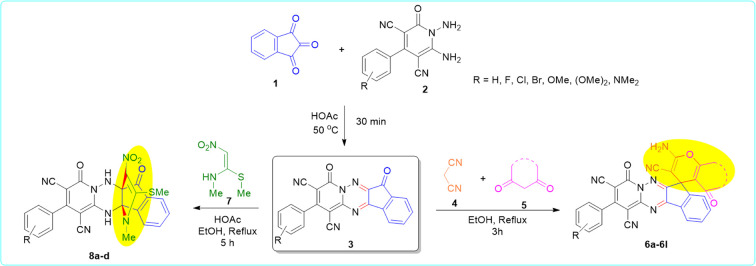

## Introduction

Fused nitrogen heterocycles are among the most special scaffolds in natural and synthetic products. Pyridotriazine derivatives are one of the significant fused nitrogen heterocycles which have displayed a broad range of biological properties such as anti-avian influenza virus (H_5_N_1_),^1^ anticancer,^2^ antifungal,^3^ anti-anxiety, anti-inflammatory,^4^ antibacterial,^5^ antioxidant,^6^ antimicrobial functions.^7^ Moreover, some examples of pyridotriazine can show optical^8^ and corrosion inhibition properties.^9^ Manifold and varied syntheses of these systems are well documented in the literature.^10–12^ Pyridotriazine moieties are commonly synthesized *via* heterocyclization reaction between 1,6-diaminopyridones and bifunctional electrophiles. As an unsymmetrical diamine, 1,6-diaminopyridone is a very active substrate for building diverse heterocyclic systems.^13^ The majority of the new reported methods for the construction of pyrido[1,2,4]-triazines, use reactions of 4-aryl-1,6-diamino-2-oxo-1,2-dihydro-pyridine-3,5-dicarbonitrile derivatives as a precursor with some active dicarbonyl compounds in the presence of a catalyst.^14–16^

**Scheme 1:**
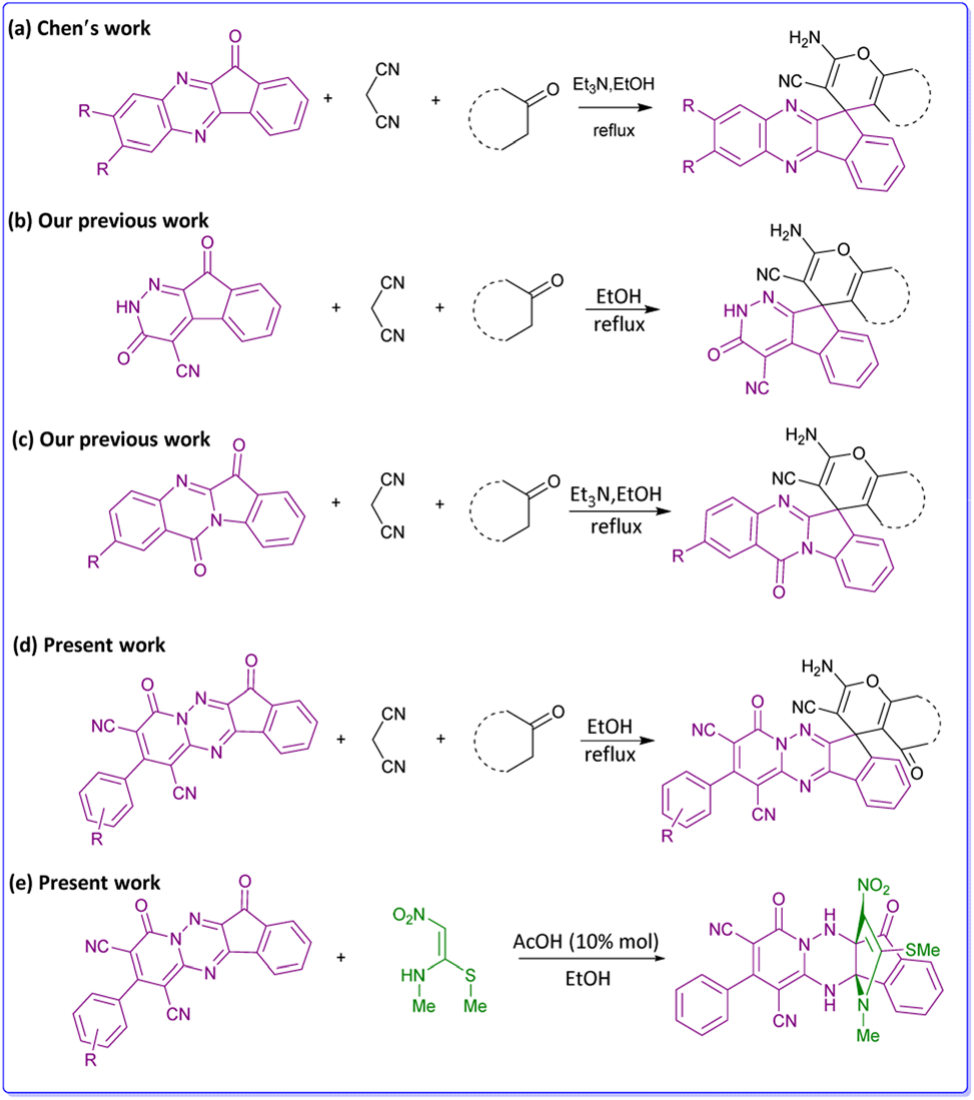
Previous studies for synthesis of spiro-4*H*-pyrans.

Diversity-Oriented Synthesis (DOS) is a new approach consists of synthetic methods to generating and developing new chemical diverse compounds libraries.^17^ DOS explores some promising new applications, that have the potential to significantly advance studies in drug discovery and chemical biology, so that increase the biological outcome of the molecules.^18,19^ Spiro compounds are a specific category of heterocycles that are extensively found in many natural products and bioactive substances.^20^ Pyran core-containing compounds also have a great variety of pharmaceutical potentials and biological sensors.^21^ In this regard, spiro-4*H*-pyrans are significant in organic synthesis because they have a wide spectrum of important biological effects that consist of anti-anaphylactic, antibacterial,^22^ antitumor, antifungal,^23^ and antirheumatic activities. Through multicomponent reactions,^24,25^ several strategies of synthesis of these compounds have been reviewed,^26–28^ particularly involving the reaction of active carbonyl compounds such as acenaphthoquinone, isatin or ninhydrin with 1,3-diketones, and malononitrile/ethyl cyanoacetate *via* a three-component reaction in the presence or absence of a catalyst.^29–31^ Recently, numerous publications reported the synthesis of these compounds with indeno[1,2-*b*]quinoxalin (Scheme 1a),^32^ indeno[2,1-*c*]pyridazine (Scheme 1b),^33^ indolo[2,1-*b*]quinazoline (Scheme 1c)^34^ instead of active carbonyl compounds. In our previous works, also we have reported the biological properties of spiro-4*H*-pyran derivatives (Scheme 1b and 1c).^35–37^ To extend these works, we decided to use indeno[1,2-*e*]pyrido[1,2-*b*][1,2,4]triazine as a main component in the reaction with malononitrile and CH-acids for building novel spiro-4*H*-pyrans (Scheme 1d). As a result, the fusion of the spiro-pyran system with the pyrido-triazine moiety could be as a privileged nucleus for many biological activities.^38^ We have successfully synthesized a series of compounds containing spiro-4*H*-pyrans attached to pyrido[1,2,4]triazine, which are completely new.

Nitrogen-containing propellane and analogs are remarkable models for the study of their synthesis, and have gained much consideration due to their pharmaceuticals.^39^ Many approaches have recently been reported for the synthesis of propellanes and the formation of framework of these compounds was discussed.^40–44^

(*E*)-*N*-Methyl-1-(methylthio)-2-nitroethenamine that have flexibility and high reactivity has shown important attention as an ambiphilic synthon and suitable framework due to the attendance of push–pull system to synthesis of numerous heterocyclic compounds and mainly exist in natural and synthetic drugs.^45,46^ In continuation of our effort, by using pyrido-triazine as a main component and NMSM as an ambiphilic synthon, we also investigate an efficient synthesis of indenopyridotriazine [4.3.3]propellans in the presence of HOAc in ethanol (Scheme 1e). So far, there are no reports on the synthesis of these structures.

## Results and discussion

The starting material, 1,6-diaminopyridinone derivatives 2 were easily synthesised from cyanoacetohydrazide with arylbenzilidenemalononitriles *via* cyclocondensation reaction.^47^ Then reaction of ninhydrin 1 and 1,6-diamino-2-oxo-4-phenyl-1,2-dihydropyridine-3,5-dicarbonitrile derivatives 2 in acetic acid as a solvent without any catalyst led to corresponding dihydroindenopyrido[1,2,4]triazine derivatives 3. Therefore, we used compound 3 to synthesize some novel spiro[indeno[1,2-*e*]pyrido[1,2-*b*][1,2,4]triazine-7,5′-pyran]-1,3,6′-tricarbonitrile derivatives 6. Initially, we have chosen pyrido[1,2,4]triazine 3a (1 mmol), malononitrile 4 (1 mmol) and *N*,*N*-dimethylbarbituric acid 5a (1 mmol) as model substrates in numerous temperatures and solvents to acquire an optimum condition which was accomplished in ethanol without any catalyst (entry 1, Table 1).

**Table 1:** Optimization conditions for the generation of 6a^a^

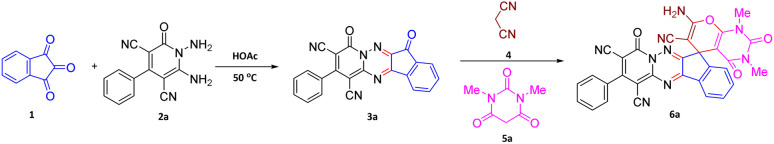					
Entry	Solvent	Catalyst	Time (h)	Temp. (°C)	Yield^b^ (%)
**1**	**EtOH**	**—**	**3**	**78**	**90%**
2	H_2_O	—	24	100	No reaction
3	EtOH	HOAc	5	78	Trace
4	EtOH	Piperidine	24	78	50%
5	EtOH	Et_3_N	10	78	61%
6	MeOH	—	10	64	45%
7	MeCN	—	24	82	58%
8	Iso-propanol	—	10	82	70%
9	DMF	—	12	120	10%

a Reaction conditions were accomplished using 1, 2a, 3a, 4, 5a (1 mmol), catalyst (0.01 mmol), and solvent (10 mL).

b Isolated yield based on 6a.

**Scheme 2:**
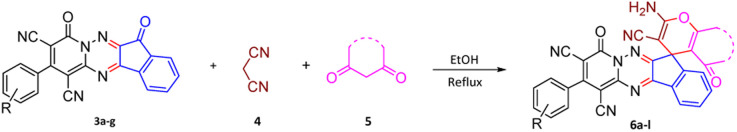
Synthetic scheme for the formation of products 6a–l.

Using the results obtained from the optimized table, we succeeded in synthesizing a three-component one-pot procedure of new spiro[indeno[1,2-*e*]pyrido[1,2-*b*][1,2,4]triazine-7,5′-pyran]-1,3,6′-tricarbonitrile derivatives 6a–l from pyrido[1,2,4]triazine derivatives 3a–g, malononitrile 4 and CH-acids 5 in EtOH under catalyst-free condition (Scheme 2).

The scope of the process is shown in Table 2. The desired compounds 6a–l were obtained in excellent yields in all cases and substituent with electron-donating/withdrawing of compounds 3a–g have not been effect in this reaction.

**Table 2:** Synthesis of spiro[indeno[1,2-*e*]pyrido[1,2-*b*][1,2,4]triazine-7,5′-pyran]-1,3,6′-tricarbonitrile 6a–l^a^

Entry	1,6-Diaminopyridone	CH-acid	Product 6	Yield (%)
1	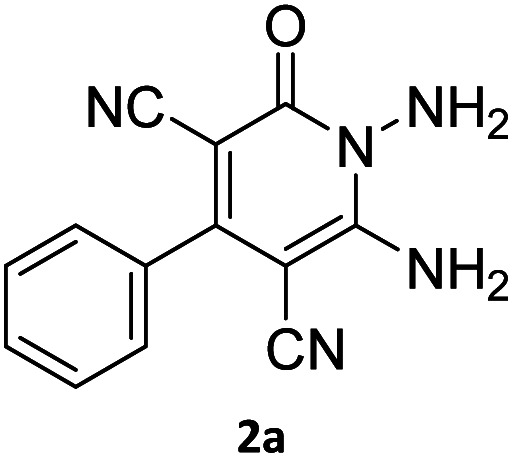	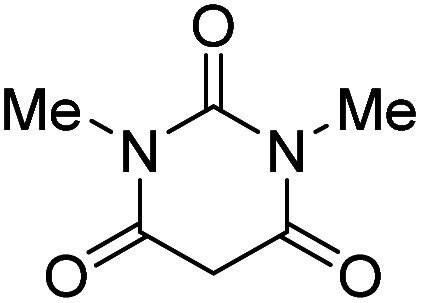	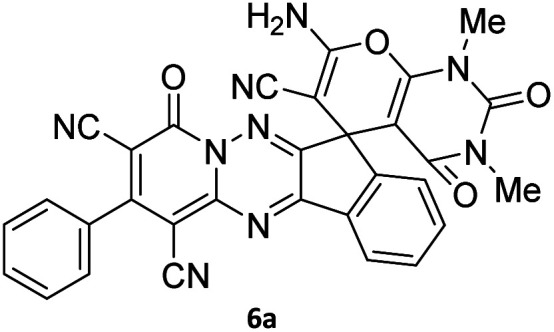	90
2	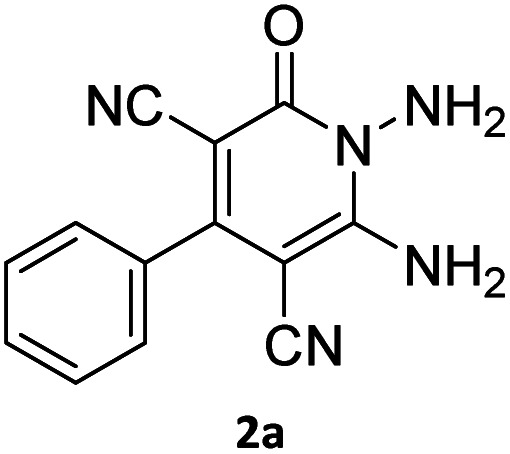	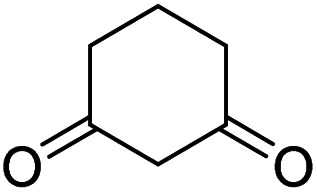	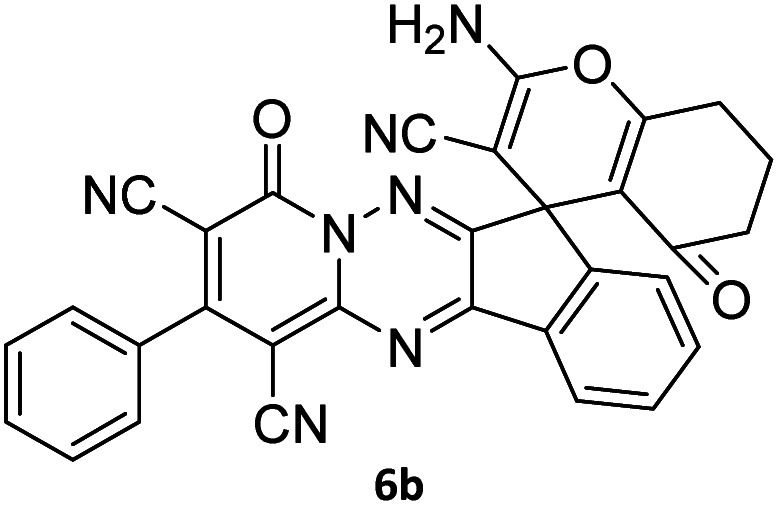	83
3	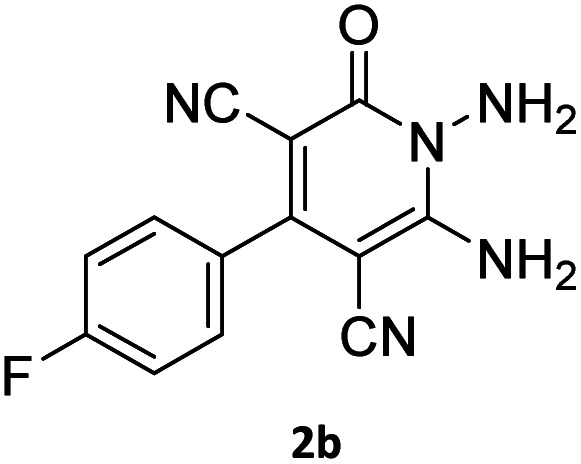	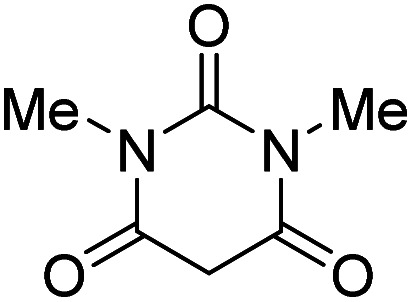	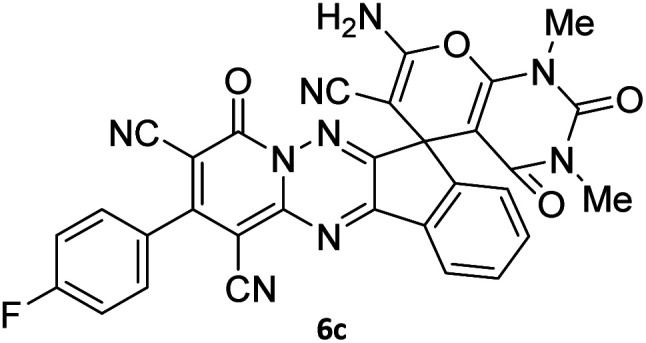	86
4	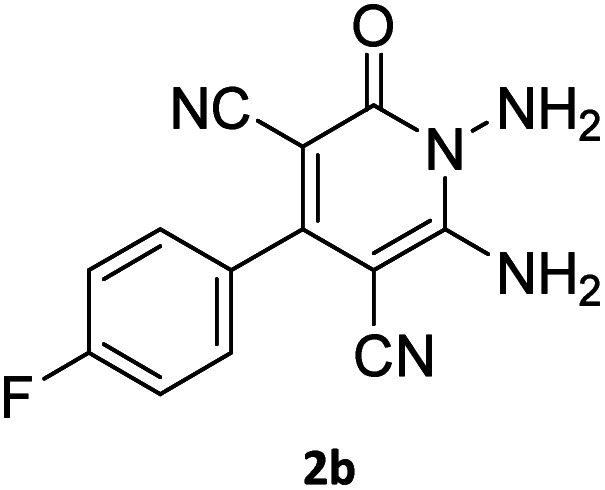	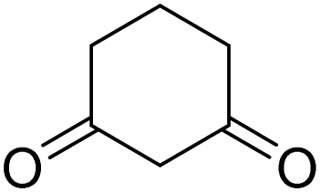	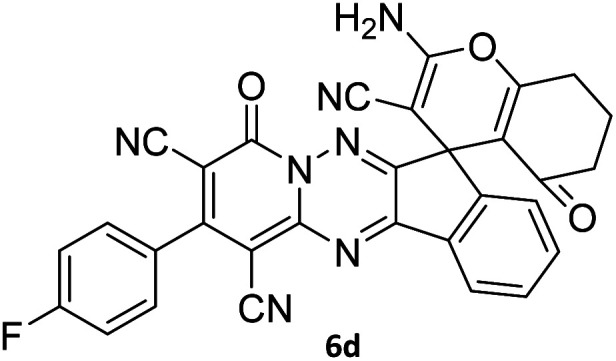	91
5	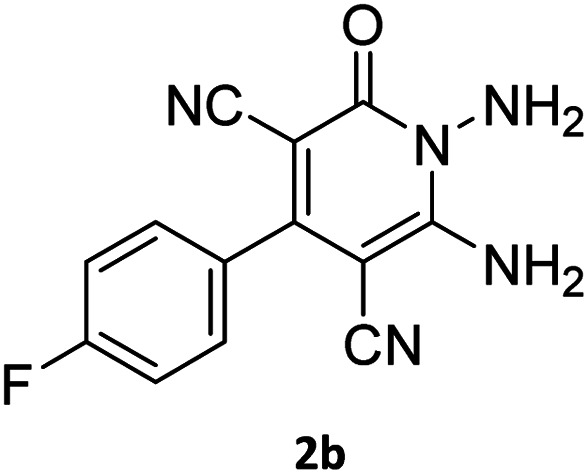	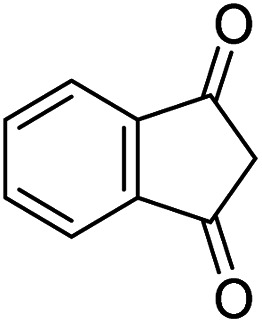	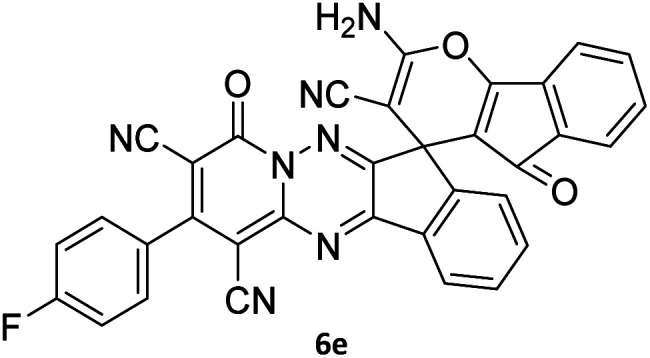	81
6	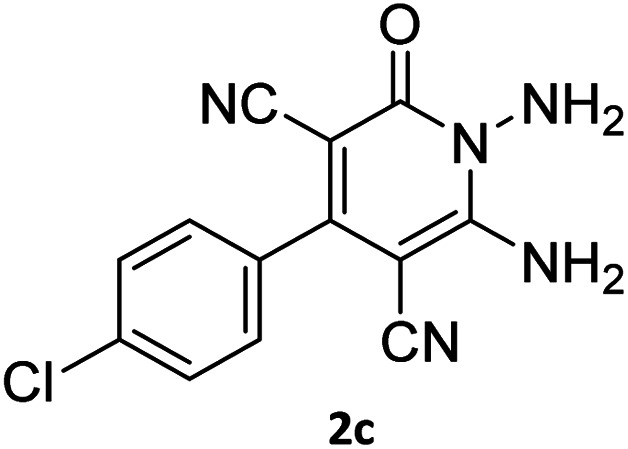	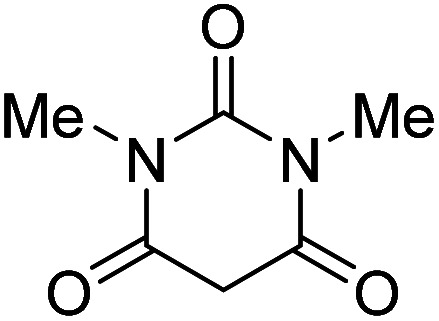	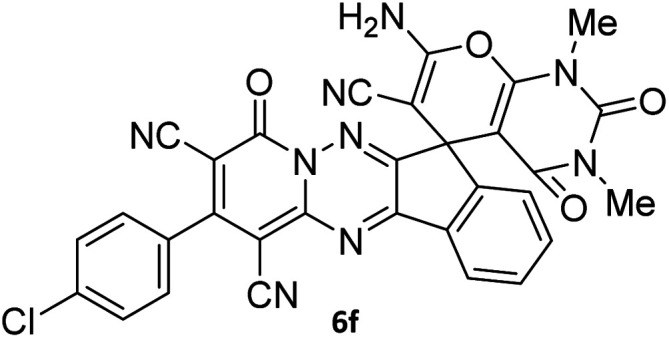	85
7	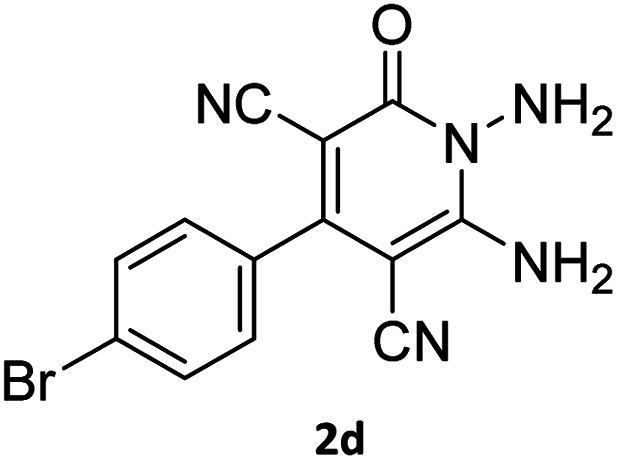	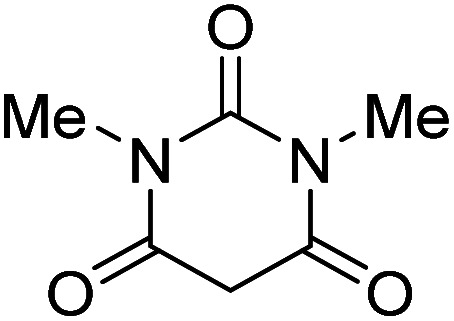	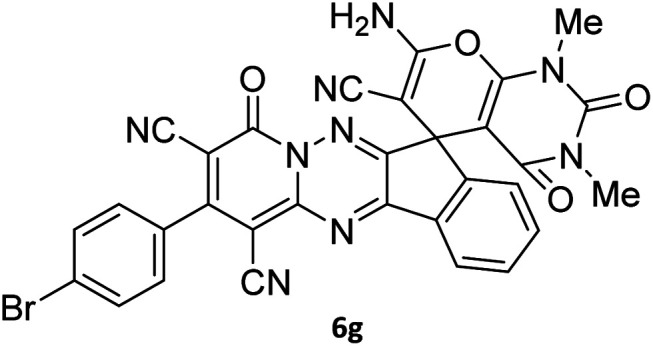	89
8	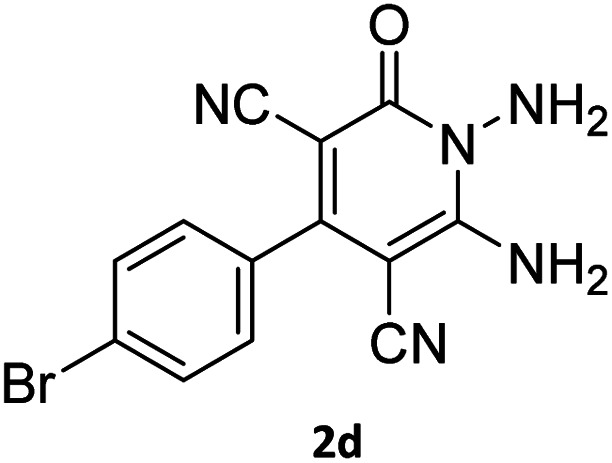	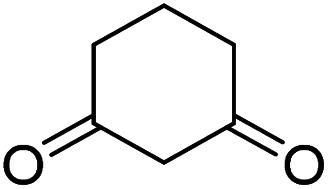	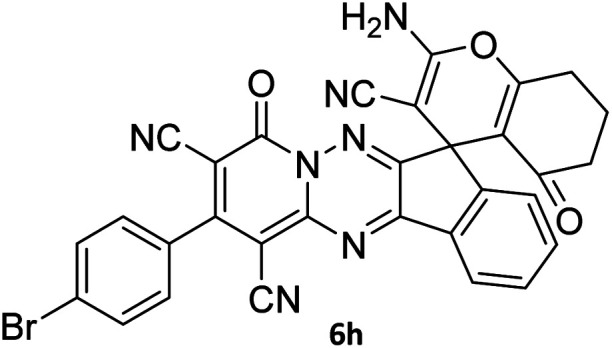	80
9	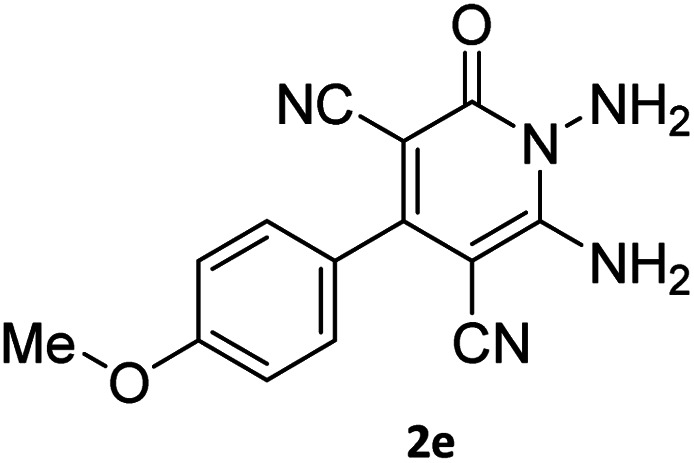	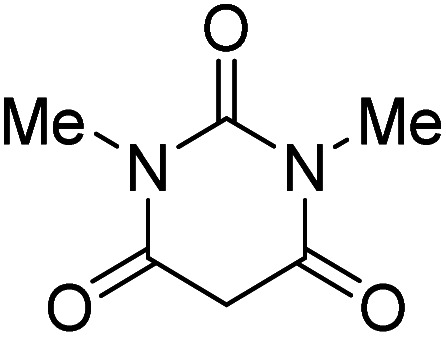	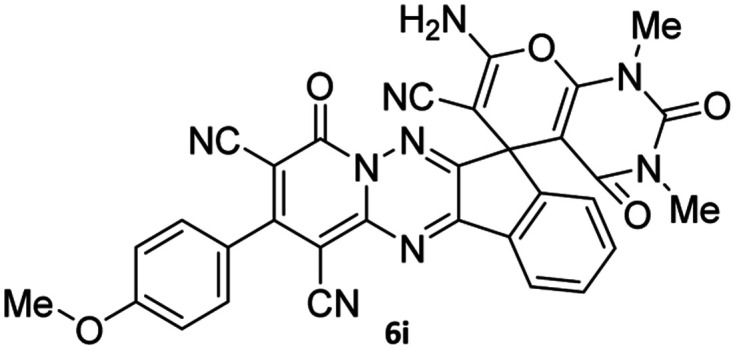	86
10	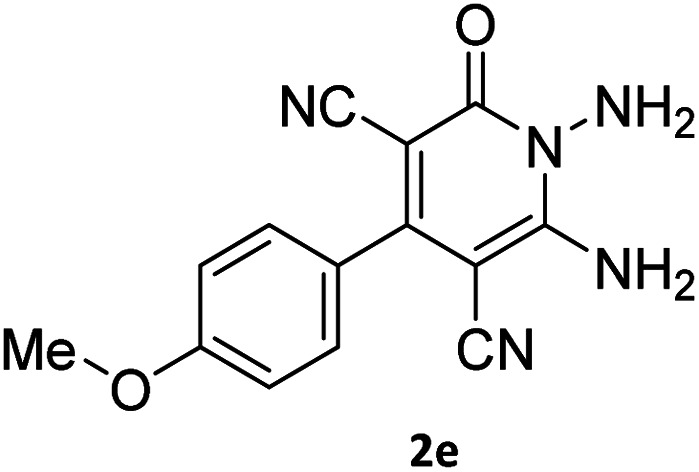	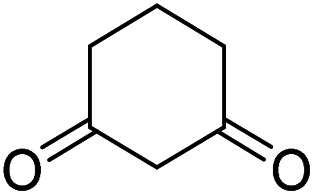	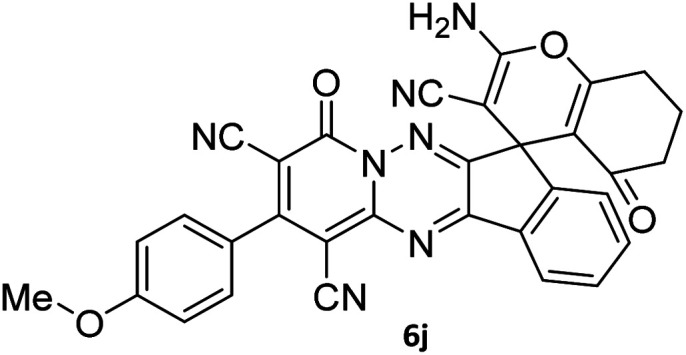	88
11	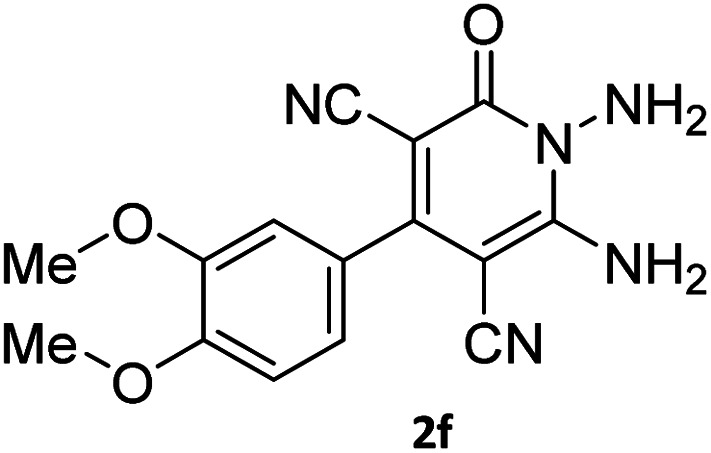	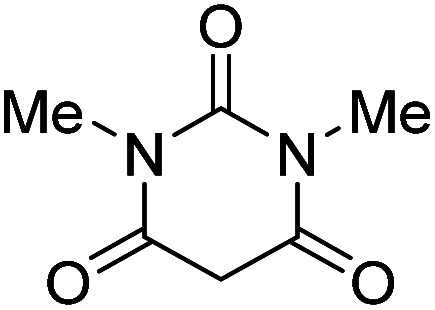	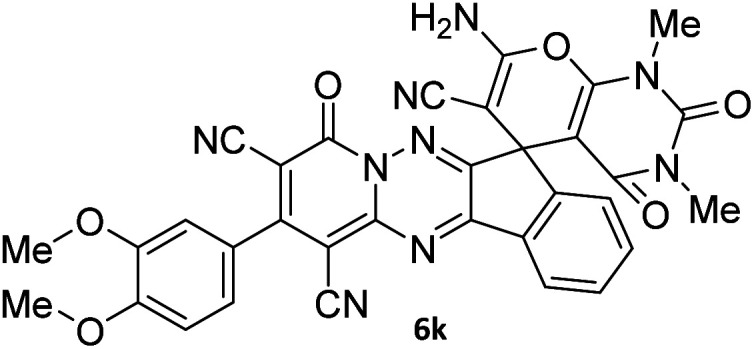	82
12	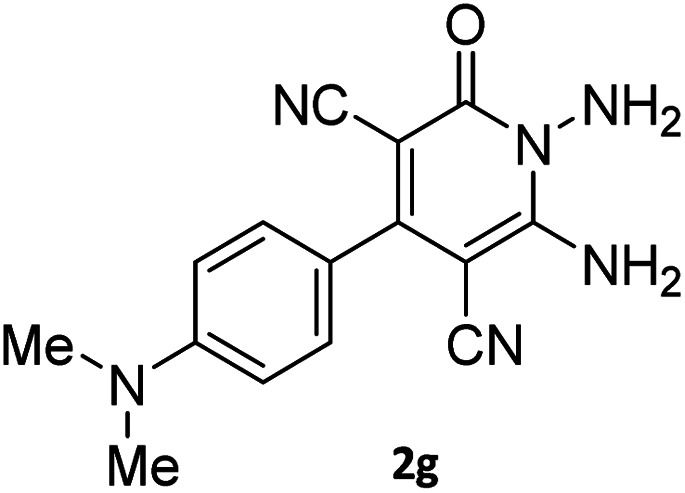	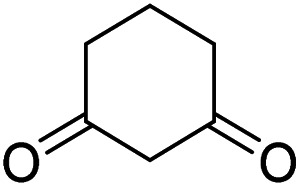	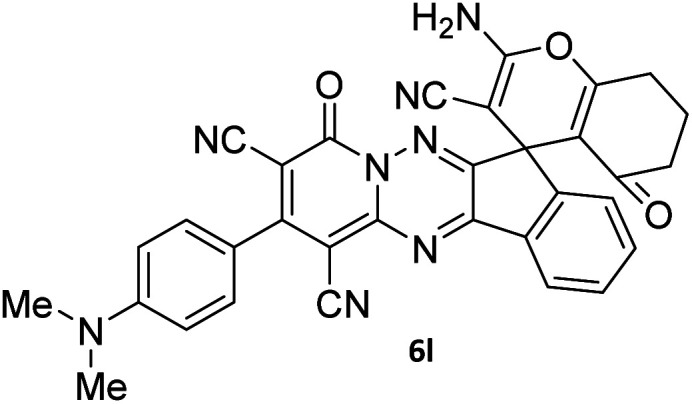	81

a The reactions were performed using 1,6-diaminopyridone (1 mmol), malononitrile (1 mmol), C–H acid (1 mmol), EtOH (10 mL).

**Fig. 1:**
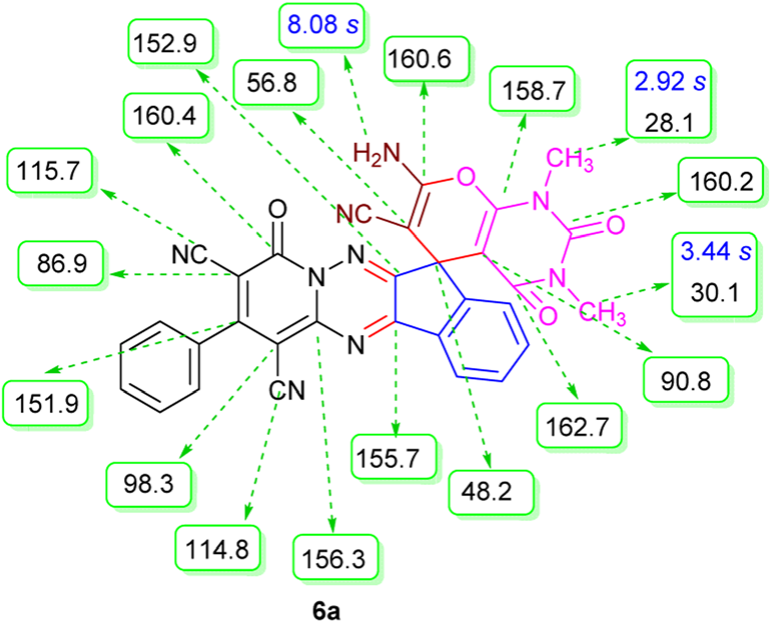
^1^H and ^13^C NMR chemical shifts of compound 6a.

The structures of products 6a–l were elucidated from their IR, ^1^H NMR, ^13^C NMR and Mass spectra that confirmed the formation of 6a–l (see the ESI†). The ^1^H NMR spectrum of 6a exhibited two signals assigned as arising from two methyl groups (*δ* 2.92 and 3.44 ppm), signals of the aromatic moiety showed multiplets (*δ* 7.61–8.21 ppm), the NH_2_ protons (*δ* 8.08 ppm) that exchangeable with D_2_O. Chemical shifts of compound 6a were completely assigned and shown in Fig. 1. The ^13^C NMR spectrum of 6a recognized 29 clear resonances which is in accordance with the proposed structure. Resonances due to spiro carbon and *C*=CNH_2_ appeared at *δ* 48.2 and 56.8 ppm respectively. Three signals at *δ* 86.9, 90.8 and 98.3 ppm were assigned to *C*CON, *C*–CONMe and *C*–CN respectively. The carbonyl group of pyridone ring was displayed at *δ* 160.6 ppm. The signal at *δ* 160.4 ppm was assigned to C–NH_2_. The characteristic signals of three CN were observed at *δ* 114.8, 115.7 and 117.0 ppm. The mass spectrum of 6a showed the molecular-ion peak at *m*/*z* 579 that confirmed the proposed structure. Absorption bands of 6a in the FT-IR spectrum displayed due to NH_2_, 3CN and 3CO groups at 3376, 2317, 2227, 2204, 1690, 1642, 1594 cm^−1^ respectively.

**Scheme 3:**
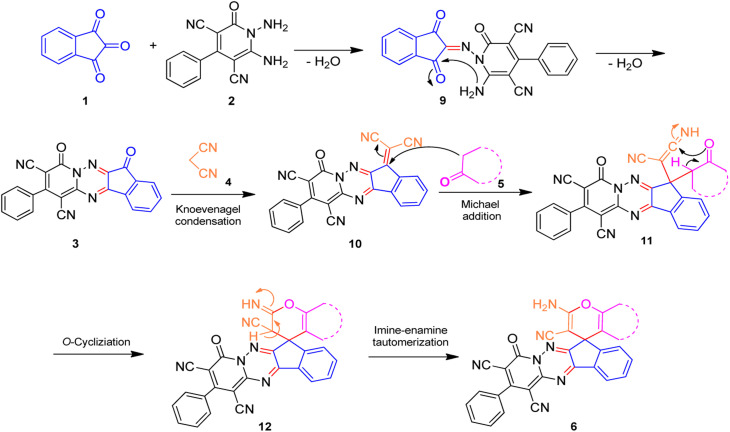
Proposed mechanism for the formation of products 6.

A proposed mechanism for the synthesis of spiroindenopyridotriazine 6 is illustrated in Scheme 3. First, condensation of ninhydrin 1 with 1,6-diaminopyridinone 2 leads to the formation of intermediate 9. Then nucleophilic attack of the NH_2_ to the carbonyl group in intermediate 9*via* intramolecular cyclization gives the corresponding pyrido[1,2,4]triazine 3. Subsequently, pyrido[1,2,4]triazine 3 and malononitrile 4 participate in the Knoevenagel condensation reaction to produce the adduct 10. Next, CH-acid compound 5 attacks adduct 10*via* its nucleophilic core to construct the Michael adduct 11, followed by intermolecular *O*-cyclization *via* nucleophilic addition of oxygen to the nitrile group that yields intermediate 12. In the final step, imine-enamine tautomerization of 12 leads to the final product 6.

To further expand the scope of these studies, we also tested nitroketene acetals as a 1,3-*N*,*C* di-nucleophilic precursor for the preparation of indenopyridotriazine [4.3.3]propellane. By using pyrido[1,2,4]triazine 3 (containing two C=N as 1,2-dielectrophilic sources) and NMSM 7 as a nitroketene *N*,*C*-acetal in the presence of HOAc as a catalyst, the [4.3.3]propellane system, was obtained. We used different solvents and catalysts for the optimization of this reaction. As a result, without catalyst, the yield was low, so the best conditions were achieved in EtOH in the vicinity of HOAc (10 mol%) at reflux condition (Table 3). When we used compounds 3 containing electron-donating groups at high temperature conditions, the pyridone moiety was separated from the final product (TLC and NMR evidence). Thus we could synthesize the [4.3.3]propellane system 8a–d with good to high yields, when 1,6-diaminopyridone have electron-withdrawing groups. In all of these reactions, all products were obtained with high efficiency and good purity (Table 4).

**Table 3:** Optimization of reaction conditions for the synthesis of 8a

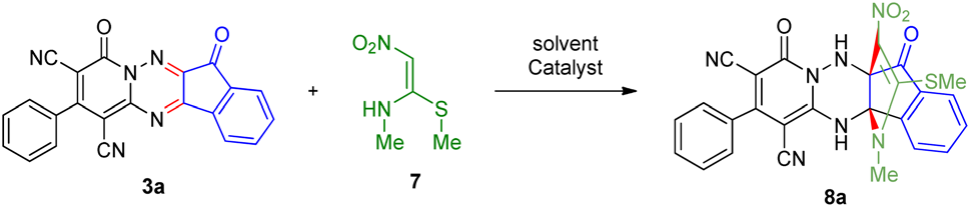					
Entry	Solvent	Catalyst	Time (h)	Temp. (°C)	Yield (%)
1	EtOH	—	24	78	Trace
**2**	**EtOH**	**HOAc**	**5**	**78**	**91**
3	EtOH	*p*-TSA	24	78	40
4	EtOH	CCl_3_COOH	20	78	50
5	EtOH	I_2_	8	78	Trace
6	EtOH	l-Proline	10	78	58
7	MeOH	—	10	64	45
8	MeCN	—	24	82	58
9	DMF	—	24	90	0
10	Neat	—	24	90	0

**Table 4:** Synthesis of indenopyridotriazine [4.3.3]propellane 8a–d

Entry	1,6-Diaminopyridone	Product 8	Yield (%)
1	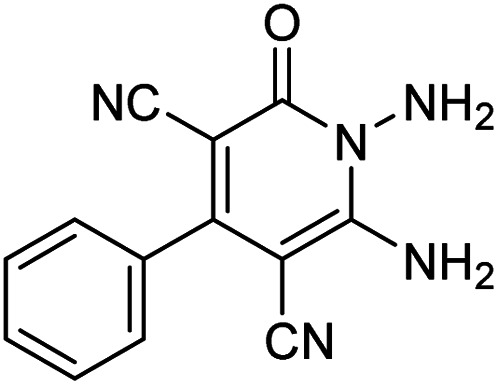	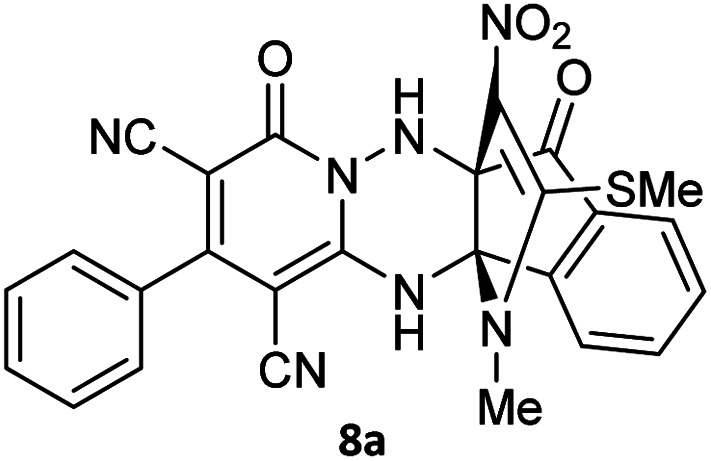	91
2	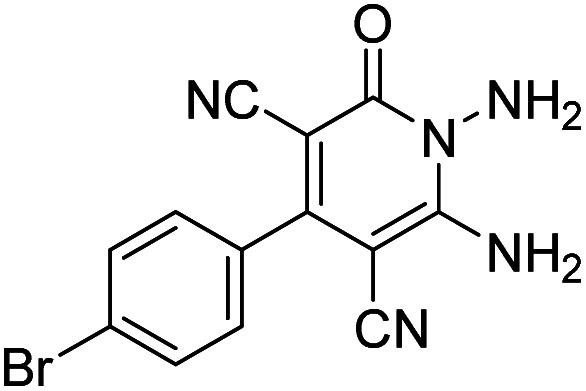	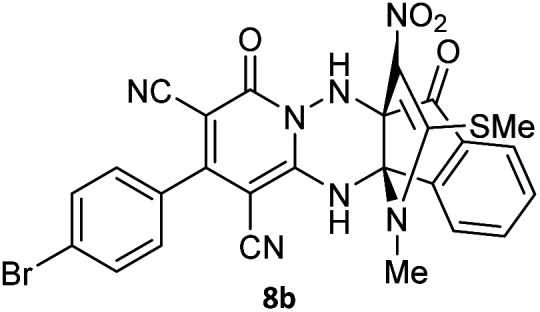	87
3	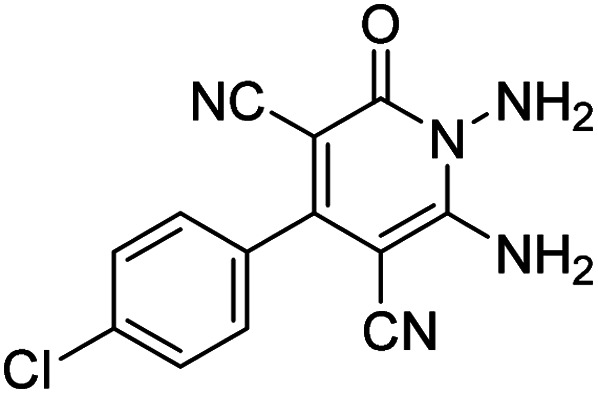	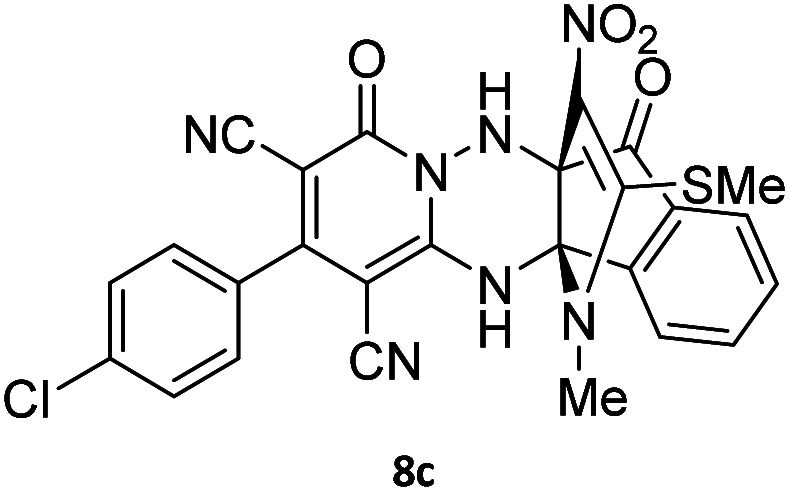	85
4	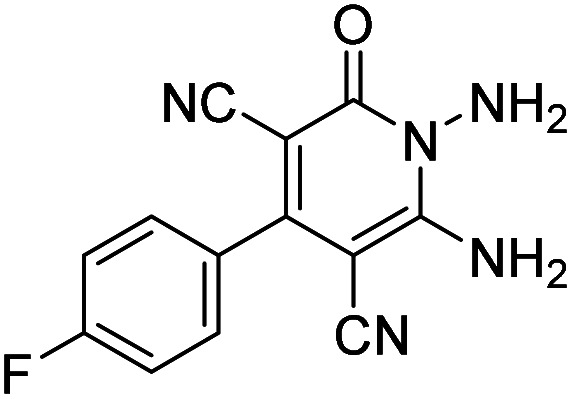	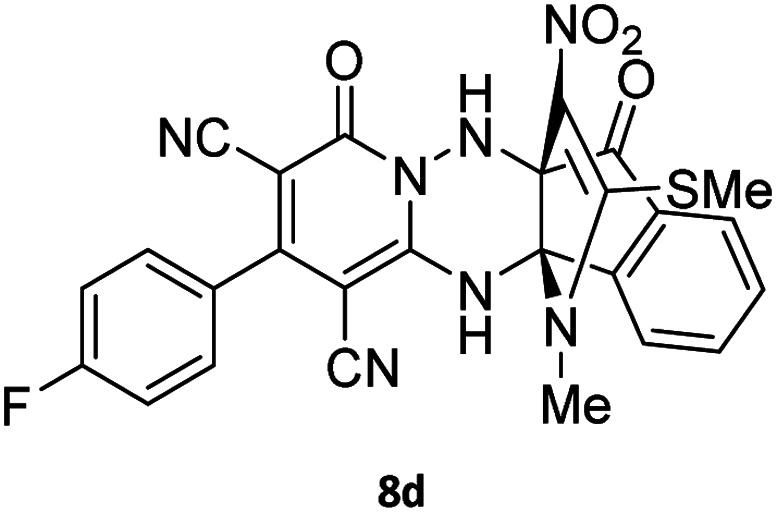	79

The low solubility of products 6 and 8 in organic solvents significantly simplified workup and purification of the products. The product is withdrawn from the reaction mixture by precipitation due to its low solubility in the solvent. However, the low solubility of these molecules made it difficult to evaluate their biological activities. Due to the low solubility of the products, NMR data could be obtained only for selected products. Just in case, it was not possible to obtain ^13^C NMR data for compound 6e due to insolubility, while in other cases NMR data could be obtained by increasing the number of scans.

**Scheme 4:**
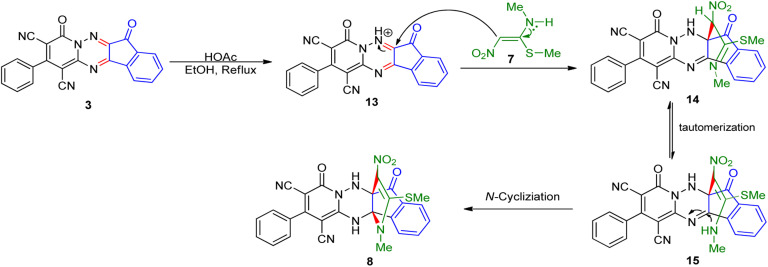
Proposed mechanism for the formation of products 8.

The structures of products 8a–d were deduced from their IR, ^1^H NMR, ^13^C NMR and Mass spectral data. The ^1^H NMR spectrum of 8a showed characteristic two signals of the N–H proton at *δ* = 8.25 and 9.64 ppm (exchangeable with D_2_O). Aromatic protons appeared at *δ* 7.49–8.61 ppm. The NCH_3_ and SCH_3_ groups exhibited two sharp singlets at 3.40 and 2.61 ppm, respectively. ^13^C NMR spectra revealed a signal at *δ* = 191.3 ppm for C=O carbon of the ninhydrin moiety and signals corresponding to the C–SMe, C–NO_2_, C–NMe groups were observed at about 160.2, 124.3 and 87.9 ppm respectively. Two nitrile groups were observed at 114.7 and 116.1 ppm. The IR spectrum of 8a displayed vibrational stretching bands for NH (3441 and 3170 cm^−1^), CN (2224 cm^−1^), C=O (1729, 1674 cm^−1^) and NO_2_ (1544 and 1388 cm^−1^) groups. Mass data also supported product 8a (see the ESI†). A probable reaction mechanism was suggested as shown in Scheme 4. First, pyrido[1,2,4]triazine 3 reacts with HOAc to give protonated triazolopyridinone 13 which reacts with NMSM 7 to generate the intermediate 14. The intermediate 14 undergoes successive tautomerization followed by *N*-cyclization *via* nucleophilic addition of nitrogen of NMSM that leads to the formation of desired products 8.

## Conclusion

We have developed the synthesis of structurally diverse products from a simple reaction followed by cyclization steps and appendage diversity. This study describes a convenient protocol for the synthesis of novel spiro[indeno[1,2-*e*]pyrido[1,2-*b*][1,2,4]triazine-7,5′-pyran]-1,3,6′-tricarbonitrile *via* the reaction of pyrido[1,2,4]triazine with malononitrile and CH-acids under reflux condition in ethanol. Also, we have expanded an easy one-pot reaction for the synthesis of indenopyridotriazine [4.3.3]propellanes from the reaction of pyrido[1,2,4]triazine and NMSM in HOAc as a catalyst. The diverse structures that we synthesized in this study are completely new and no reports of them have been presented, and the biological evaluations of these derivatives are underway.

## Experimental

### Materials

All commercially available chemical compounds and other solvents in this study were purchased from Merck and Aldrich chemical companies. The NMR spectra for synthesized compounds were acquired using a Bruker DRX-300 AVANCE instrument (300 MHz for ^1^H and 75.4 MHz for ^13^C) with DMSO-*d*_6_ as the deuterated solvent. Chemical shifts were reported in ppm (*δ*) relative to the internal TMS, and the coupling constant (*J*) given in hertz (Hz). FT-IR spectra and melting points of all the compounds were measured with a Bruker Tensor 27 spectrometer and electrothermal 9100 apparatus, respectively. Mass spectra were given by an Agilent 5975C VL MSD with a Triple-Axis detector operating at an ionization potential of 70 eV.

#### General procedure of the synthesis of pyrido[1,2,4]triazine compounds (3a–g)

In a 10 mL round-bottomed flask mounted over a magnetic stirrer a mixture of ninhydrin 1 (2.0 mmol) and 1,6-diaminopyridinone derivatives 2 (2.0 mmol) and HOAc 10 mL was located. The contents were stirred magnetically in an oil-bath maintained at 50 °C (30–60 min). The progress of the reaction was monitored with TLC (using ethyl acetate–*n*-hexane 1 : 1), after the reaction was completed; the reaction mixture was cooled to room temperature. Then, the solid was separated by filtration, washed twice times with water, and was subsequently washed with Et_2_O. The precipitate was dried in 130 °C to obtain crude product 3 without recrystallization.^48^

#### Procedure of the synthesis of spiroindenopyridotriazine-4*H*-pyran compounds (6a–l)

The stoichiometric mixture of pyrido[1,2,4]triazine 3 (1 mmol), malononitrile 4 (1 mmol) and cyclic CH-acid 5 (1 mmol) in EtOH (10 mL) was stirred at reflux for maximum 3 hours. The reaction time was monitored by TLC and after completion of the reaction, the reaction mixture was cooled to room temperature and filtered to give the crude product. The solid was washed with cold EtOH to give pure product 6a–l.

#### Procedure of the synthesis of indenopyridotriazine [4.3.3]propellanes (8a–d)

The mixture of pyrido[1,2,4]triazine 3 (1 mmol), NMSM 7 (1 mmol) and HOAc (0.1 mmol, 10 mol%) in EtOH (10 mL) was stirred at reflux for 5–6 hours. The progress of completion of the reaction was controlled with TLC monitoring, then the precipitate that formed in this reaction was filtered after cold at room temperature and was washed with cold EtOH to give the pure product 8.

#### 4,7-Dioxo-2-phenyl-4,7-dihydroindeno[1,2-*e*]pyrido[1,2-*b*][1,2,4]triazine-1,3-dicarbonitrile (3a)

Prepared according to the general procedure, orange crystal, 88%, mp: 325–327 °C, IR (KBr): 2222 (CN), 1698, 1736 (C=O), 1473 (C=C), cm^−1^; ^1^H NMR (300 MHz, DMSO-*d*_6_) *δ*: 8.33–8.36 (1H, m, ArH), 8.18–8.20 (1H, m, ArH), 8.08–8.10 (2H, m, ArH), 7.65–7.68 (5H, m, ArH); ^13^C NMR (75.4 MHz, DMSO-*d*_6_) *δ*: 160.7, 183.6 (C=O), 156.2, 160.5 (C=N), 151.2, 148.3, 141.0, 138.5, 138.1, 137.2, 133.7, 131.6, 129.4, 128.8, 125.9, 125.6, 115.35, 114.4 (Ar), 91.7, 99.8 (CN).

#### 7′-Amino-1′,3′-dimethyl-2′,4,4′-trioxo-2-phenyl-1′,2′,3′,4′-tetrahydro-4*H*-spiro[indeno[1,2-*e*]pyrido[1,2-*b*][1,2,4]triazine-7,5′-pyrano[2,3-*d*]pyrimidine]-1,3,6′-tricarbonitrile (6a)

Orange powder, 90%, mp: 286–288 °C; IR (KBr): 3376, 2354, 2317, 2227, 1690, 1642, 1596, 1547, 1468, 1386, 1201, 763 cm^−1^; ^1^H NMR (300 MHz, DMSO-*d*_6_) *δ*: 8.20 (1H, d, ^3^*J*_HH_ = 7.0 Hz, ArH), 8.08 (2H, s, NH_2_), 7.93–7.98 (1H, m, ArH), 7.86–7.89 (1H, m, ArH), 7.69–7.74 (1H, m, ArH), 7.61–7.62 (5H, m, ArH), 3.44 (3H, s, NCH_3_), 2.92 (3H, s, NCH_3_); ^13^C NMR (75.4 MHz, DMSO-*d*_6_) *δ*: 162.7 (CONMe), 160.6 (CONN), 160.4 (CNH_2_), 160.2 (CO(NMe)_2_), 158.7 (CNMe), 156.3 (CNN), 155.7 (C=N), 152.9 (C=N–N), 150.0 (*C*C=N), 151.9, 138.9, 134.2, 131.8, 131.4, 131.2, 129.3, 128.7, 126.5, 124.9 (Ar), 117.0, 115.7, 114.8 (3CN), 98.3 (*C*–CN), 90.8 (*C*–CONMe), 86.9 (*C*CON), 56.8 (*C*=CNH_2_), 48.2 (C_spiro_), 30.1 (NCH_3_), 28.1 (NCH_3_); MS (EI, 70 eV): *m*/*z* (%) = 579 (12) [M]^+^, 515 (12), 423 (30), 254 (23), 156 (100), 129 (31), 83 (38), 56 (69).

#### 2-Amino-4′,5-dioxo-2′-phenyl-5,6,7,8-tetrahydro-4′*H*-spiro[chromene-4,7′-indeno[1,2-*e*]pyrido[1,2-*b*][1,2,4]triazine]-1′,3,3′-tricarbonitrile (6b)

Orange powder; 83%, mp: 296–298 °C; IR (KBr): 3191, 2223, 2195, 1672, 1594, 1548, 1488, 1435, 1356, 1308, 1223, 1157, 1009, 750 cm^−1^; ^1^H NMR (300 MHz, DMSO-*d*_6_) *δ*: 8.19 (1H, d, ^3^*J*_HH_ = 9.0 Hz, ArH), 7.91–7.96 (1H, m, ArH), 7.78–7.81 (1H, m, ArH), 7.78 (2H, s, NH_2_), 7.67–7.72 (1H, m, ArH), 7.61–7.62 (5H, m, ArH), 2.77–2.84 (2H, m, COCH_2_), 2.17–2.19 (2H, m, CH_2_), 1.94–1.97 (2H, m, CH_2_); ^13^C NMR (75.4 MHz, DMSO-*d*_6_) *δ*: 196.2 (*C*OCH_2_), 169.1 (CONN), 163.1 (CNH_2_), 160.8 (CNN), 160.3 (C=N), 159.1 (C=N–N), 156.3 (=*C*–O), 151.8 (*C*C=N), 156.1, 138.9, 134.2, 131.6, 131.1, 129.3, 128.7, 126.1, 125.7, 125.0 (Ar), 117.6, 115.7, 114.9 (3CN), 111.9 (*C*–CN), 98.3 (*C*–COCH_2_), 90.7 (*C*CON), 56.6 (*C*=CNH_2_), 47.7 (C_spiro_), 36.5 (O=C–*C*H_2_), 27.3 (=C–*C*H_2_), 20.0 (CH_2_); MS (EI, 70 eV): *m*/*z* (%) = 535 (8) [M]^+^, 436 (3), 281 (11), 236 (21), 207 (21), 149 (22), 129 (20), 84 (49), 55 (100).

#### 7′-Amino-2-(4-fluorophenyl)-1′,3′-dimethyl-2′,4,4′-trioxo-1′,2′,3′,4′-tetrahydro-4*H*-spiro[indeno[1,2-*e*]pyrido[1,2-*b*][1,2,4]triazine-7,5′-pyrano[2,3-*d*]pyrimidine]-1,3,6′-tricarbonitrile (6c)

Orange powder, 86%, mp: 253–255 °C; IR (KBr): 3166, 2224, 1685, 1650, 1601, 1547, 1462, 1386, 1237, 1162, 843, 776 cm^−1^; ^1^H NMR (300 MHz, DMSO-*d*_6_) *δ*: 8.21 (1H, d, ^3^*J*_HH_ = 6.5 Hz, ArH), 8.09 (2H, s, NH_2_), 7.93–7.98 (1H, m, ArH), 7.87–7.89 (1H, m, ArH), 7.66–7.75 (3H, m, ArH), 7.47–7.53 (2H, m, ArH), 3.44 (3H, s, NCH_3_), 2.92 (3H, s, NCH_3_); ^13^C NMR (75.4 MHz, DMSO-*d*_6_) *δ*: 162.7 (CONMe), 162.2 (CONN), 161.4 (*d*, ^1^*J*_CF_ = 245.0 Hz), 160.3 (CNH_2_), 159.4 (CO(NMe)_2_), 158.7 (CNMe), 156.2 (CNN), 155.7 (C=N), 152.9 (C=N–N), 150.0 (*C*C=N), 151.93, 139.01, 132.5 (*d*, ^4^*J*_CF_ = 6.5 Hz), 131.35 (*d*, ^3^*J*_CF_ = 8.2 Hz), 130.6, 124.9, 126.5, 117.0, 115.5 (*d*, ^2^*J*_CF_ = 22 Hz, Ar), 116.7, 116.4, 114.8 (3CN), 98.4 (*C*–CN), 90.9 (*C*–CONMe), 86.9 (*C*CON), 56.7 (*C*=CNH_2_), 48.2 (C_spiro_), 30.1 (NCH_3_), 28.1 (NCH_3_); MS (EI, 70 eV): *m*/*z* (%) = 597 (5) [M]^+^, 441 (47), 413 (22), 254 (13), 156 (100), 129 (14), 99 (20), 66 (47).

#### 2-Amino-2′-(4-fluorophenyl)-4′,5-dioxo-5,6,7,8-tetrahydro-4′*H*-spiro[chromene-4,7′-indeno[1,2-*e*]pyrido[1,2-*b*][1,2,4]triazine]-1′,3,3′-tricarbonitrile (6d)

Dark orange powder; 91%, mp: 296–298 °C; IR (KBr): 3198, 2224, 2197, 1674, 1599, 1548, 1463, 1354, 1227, 1161, 845, 772 cm^−1^; ^1^H NMR (300 MHz, DMSO-*d*_6_) *δ*: 8.20 (1H, d, ^3^*J*_HH_ = 6.8 Hz, ArH), 7.91–7.96 (1H, m, ArH), 7.78–7.81 (1H, m, ArH), 7.78 (2H, s, NH_2_), 7.65–7.72 (3H, m, ArH), 7.47–7.53 (2H, m, ArH), 2.77–2.48 (2H, m, COCH_2_), 2.17–2.21 (2H, m, CH_2_), 2.06 (acetone), 1.92–1.97 (2H, m, CH_2_); ^13^C NMR (75.4 MHz, DMSO-*d*_6_) *δ*: 196.2 (*C*OCH_2_), 168.1 (CONN), 165.5 (d, ^1^*J*_CF_ = 247.0 Hz), 163.2 (CNH_2_), 162.2 (CNN), 160.8 (C=N), 159.3 (C=N–N), 159.1 (=*C*–O), 156.2 (*C*C=N), 156.1, 151.80, 139.0, 131.5, 131.4 (d, ^3^*J*_CF_ = 9.0 Hz), 131.1, 126.1, 125.0, 117.61, 116.5 (d, ^2^*J*_CF_ = 22.5 Hz, ArH), 116.4, 115.7, 114.8 (3CN), 111.9 (*C*–CN), 98.4 (*C*–COCH_2_), 90.8 (*C*CON), 56.6 (*C*=CNH_2_), 47.7 (C_spiro_), 36.5 (O=C–*C*H_2_), 31.1 (=C–*C*H_2_), 20.0 (CH_2_). MS (EI, 70 eV): *m*/*z* (%) = 553 (2) [M]^+^, 551 (20), 495 (9), 368 (100), 313 (31), 236 (37), 213 (10), 135 (15), 83 (47), 57 (63).

#### 2-Amino-2′-(4-fluorophenyl)-4′,5-dioxo-4′*H*,5*H*-spiro[indeno[1,2-*b*]pyran-4,7′-indeno[1,2-*e*]pyrido[1,2-*b*][1,2,4]triazine]-1′,3,3′-tricarbonitrile (6e)

Orange powder, 81%, mp: 258–260 °C; IR (KBr): 3195, 2223, 1681, 1634, 1599, 1550, 1459, 1360, 1240, 1162, 847, 770 cm^−1^; ^1^H NMR (300 MHz, DMSO-*d*_6_) *δ*: 8.28 (1H, *d*, ^3^*J*_HH_ = 9 Hz, ArH), 8.19 (2H, s, NH_2_), 7.99–8.03 (2H, m, ArH), 7.77–7.82 (1H, m, ArH), 7.58–7.69 (3H, m, ArH), 7.48–7.54 (3H, m, ArH), 7.40–7.46 (1H, m, ArH), 7.32 (1H, d, ^3^*J*_HH_ = 6 Hz, ArH). MS (EI, 70 eV): *m*/*z* (%) = 587, (0.4) [M]^+^, 521 (6), 441 (13), 269 (15), 208 (20), 146 (100), 104 (62), 66 (75).

#### 7′-Amino-2-(4-chlorophenyl)-1′,3′-dimethyl-2′,4,4′-trioxo-1′,2′,3′,4′-tetrahydro-4*H*-spiro[indeno[1,2-*e*]pyrido[1,2-*b*][1,2,4]triazine-7,5′-pyrano[2,3-*d*]pyrimidine]-1,3,6′-tricarbonitrile (6f)

Orange powder, 85%, mp: 273–275 °C; IR (KBr): 3451, 3325, 2223, 1680, 1650, 1593, 1544, 1462, 1387, 1243, 1188, 1160, 1090, 967, 835, 775 cm^−1^; ^1^H NMR (300 MHz, DMSO-*d*_6_) *δ*: 8.21 (1H, d, ^3^*J*_HH_ = 6 Hz, ArH), 8.09 (2H, s, NH_2_), 7.93–7.98 (1H, m, ArH), 7.87–7.89 (1H, m, ArH), 7.72–7.75 (3H, m, ArH), 7.62–7.64 (2H, m, ArH), 3.44 (3H, s, NCH_3_), 2.91 (3H, s, NCH_3_); ^13^C NMR (75.4 MHz, DMSO-*d*_6_) *δ*: 162.5 (CONMe), 160.6 (CONN), 160.2 (CNH_2_), 159.9 (CO(NMe)_2_), 158.6 (CNMe), 156.3 (CNN), 155.7 (C=N), 153.0 (C=N–N), 151.8 (C-4), 150.0 (*C*C=N), 151.3, 148.7, 138.9, 131.8, 131.4, 125.9, 122.2, 117.1, 116.0 (Ar), 115.1, 112.6, 112.0 (3CN), 97.9 (*C*–CN), 90.9 (*C*–CONMe), 86.9 (*C*CON), 56.49 (*C*=CNH_2_), 48.1 (C_spiro_), 30.1 (NCH_3_), 28.1 (NCH_3_); MS (EI, 70 eV): *m*/*z* (%) = 575 (3) [M], 463 (3), 368 (7), 284 (17), 256 (32), 185 (22), 129 (41), 97 (55), 57 (100).

#### 7′-Amino-2-(4-bromophenyl)-1′,3′-dimethyl-2′,4,4′-trioxo1′,2′,3′,4′-tetrahydro-4*H*-spiro[indeno[1,2-*e*]pyrido[1,2-*b*][1,2,4]triazine-7,5′-pyrano[2,3-*d*]pyrimidine]-1,3,6′-tricarbonitrile (6g)

Orange powder, 89%, mp: 271–273 °C; IR (KBr): 3357, 3198, 2924, 2224, 2198, 1673, 1593, 1461, 1384, 1355, 1221, 1128, 1073, 835, 771, 535 cm^−1^; ^1^H NMR (300 MHz, DMSO-*d*_6_) *δ*: 8.21 (1H, d, ^3^*J*_HH_ = 6 Hz, ArH), 8.09 (2H, s, NH_2_), 7.93–7.98 (1H, m, ArH), 7.86–7.89 (3H, m, ArH), 7.69–7.74 (1H, m, ArH), 7.54–7.57 (2H, m, ArH), 3.44 (3H, s, NCH_3_), 2.91 (3H, s, NCH_3_); ^13^C NMR (75.4 MHz, DMSO-*d*_6_) *δ*: 162.9 (CONMe), 160.8 (CONN), 160.2 (CNH_2_), 159.3 (CO(NMe)_2_), 158.7 (CNMe), 156.1 (CNN), 155.7 (C=N), 153.0 (C=N–N), 152.0 (C-4), 150.0 (*C*C=N), 139.1, 133.4, 132.5, 131.8, 131.4, 130.8, 126.5, 125.0, 124.9 (Ar), 117.0, 115.6, 114.7 (3CN), 98.2 (*C*–CN), 90.7 (*C*–CONMe), 86.8 (*C*CON), 56.3 (*C*=CNH_2_), 48.2 (C_spiro_), 30.1 (NCH_3_), 28.1 (NCH_3_); MS (EI, 70 eV): *m*/*z* (%) = 551 (11), 523 (10), 368 (15), 313 (18), 236 (27), 207 (24), 149 (56), 107 (40), 83 (55), 57 (100).

#### 2-Amino-2′-(4-bromophenyl)-4′,5-dioxo-5,6,7,8-tetrahydro-4′*H*-spiro[chromene-4,7′-indeno[1,2-*e*]pyrido[1,2-*b*][1,2,4]triazine]-1′,3,3′-tricarbonitrile (6h)

Dark orange powder; 80%, mp: 250–252 °C; ^1^H NMR (300 MHz, DMSO-*d*_6_) *δ*: 8.20 (1H, d, ^3^*J*_HH_ = 6.0 Hz, ArH), 7.94–7.96 (1H, m, ArH), 7.86–7.91 (2H, m, ArH), 7.78–7.81 (1H, m, ArH), 7.78 (2H, s, NH_2_), 7.68–7.73 (1H, m, ArH), 7.54–7.57 (2H, d, ArH), 2.78–2.82 (2H, m, COCH_2_), 2.19–2.17 (2H, m, CH_2_), 2.07 (acetone), 1.93–1.97 (2H, m, CH_2_); ^13^C NMR (75.4 MHz, DMSO-*d*_6_) *δ*: 196.2 (*C*OCH_2_), 168.1 (CONN), 163.3 (CNH_2_), 160.9 (CNN), 159.3 (C=N), 159.2 (C=N–N), 156.3 (=*C*–O), 156.2 (C-4), 151.8 (*C*C=N), 152.7, 139.0, 133.4, 132.4, 131.5, 131.1, 130.8, 126.1, 125.0 (Ar), 117.6, 115.6, 114.8 (3CN), 111.9, 98.2 (*C*–COCH_2_), 90.6 (*C*CON), 56.5 (=*C*–CN), 47.7 (C_spiro_), 36.5 (O=C–*C*H_2_), 31.1 (=C–*C*H_2_), 20.0 (CH_2_).

#### 7′-Amino-2-(4-methoxyphenyl)-1′,3′-dimethyl-2′,4,4′-trioxo-1′,2′,3′,4′-tetrahydro-4*H*-spiro[indeno[1,2-*e*]pyrido[1,2-*b*][1,2,4]triazine-7,5′-pyrano[2,3-*d*]pyrimidine]-1,3,6′-tricarbonitrile (6i)

Orange powder, 86%, mp: 252–254 °C; IR (KBr): 3665, 3635, 3322, 3161, 2922, 2355, 2328, 2220, 1720, 1676, 1641, 1546, 1461, 1387, 1296, 1175, 835 cm^−1^; ^1^H NMR (300 MHz, DMSO-*d*_6_) *δ*: 8.19 (1H, d, ^3^*J*_HH_ = 9 Hz, ArH), 8.08 (2H, s, NH_2_), 7.86–7.97 (2H, m, ArH), 7.69–7.74 (1H, m, ArH), 7.58 (1H, d, ^3^*J*_HH_ = 9 Hz, ArH), 7.18 (1H, d, ^3^*J*_HH_ = 9 Hz, ArH), 3.86 (3H, s, OCH_3_), 3.44 (3H, s, NCH_3_), 2.91 (3H, s, NCH_3_); ^13^C NMR (75.4 MHz, DMSO-*d*_6_) *δ*: 162.5 (CONMe), 161.7 (CONN), 160.6 (CNH_2_), 160.2 (CO(NMe)_2_), 160.0 (CNMe), 158.6 (CNN), 156.3 (C=N), 155.6 (C=N–N), 152.9 (C-4), 150.0 (*C*C=N), 151.9, 138.8, 131.8, 131.4, 130.7, 130.3, 126.5, 126.0, 124.8 (Ar), 117.0, 116.0, 114.7 (3CN), 98.0 (*C*–CN), 90.8 (*C*–CONMe), 86.9 (*C*CON), 56.8 (*C*=CNH_2_), 55.9 (OCH_3_), 48.2 (C_spiro_), 30.1 (NCH_3_), 28.1 (NCH_3_).

#### 2-Amino-2′-(4-methoxyphenyl)-4′,5-dioxo-5,6,7,8-tetrahydro-4′*H*-spiro[chromene-4,7′-indeno[1,2-*e*]pyrido[1,2-*b*][1,2,4]triazine]-1′,3,3′-tricarbonitrile (6j)

Orange powder; 88%, mp: 250–252 °C; ^1^H NMR (300 MHz, DMSO-*d*_6_) *δ*: 8.19 (1H, d, ^3^*J*_HH_ = 6.5 Hz, ArH), 7.91–7.96 (1H, m, ArH), 7.78–7.80 (1H, m, ArH), 7.78 (2H, s, NH_2_), 7.67–7.72 (1H, m, ArH), 7.57 (2H, ^3^*J*_HH_ = 9.0 Hz, ArH), 7.18 (2H, d, ^3^*J*_HH_ = 9 Hz, ArH), 3.86 (3H, s, OMe), 2.78–2.82 (2H, m, COCH_2_), 2.17–2.21 (2H, m, CH_2_), 2.07 (acetone), 1.93–1.97 (2H, m, CH_2_); ^13^C NMR (75.4 MHz, DMSO-*d*_6_) *δ*: 196.2 (*C*OCH_2_), 168.0 (CONN), 163.0 (CNH_2_), 161.7 (CNN), 160.7 (C=N), 160.0 (C=N–N), 159.1 (=*C*–O), 156.4 (C-4), 151.7 (*C*C=N), 156.1, 138.8, 131.6, 131.1, 130.7, 126.0, 124.9, 117.6, 116.0 (Ar), 115.1, 114.7, 114.3 (3CN), 111.9 (*C*–CN), 98.0 (*C*–COCH_2_), 90.8 (*C*CON), 56.7 (*C*=CNH_2_), 55.9 (OCH_3_), 47.6 (C_spiro_), 36.5 (O=C–*C*H_2_), 31.1 (=C–*C*H_2_), 20.0 (CH_2_).

#### 7′-Amino-2-(3,4-dimethoxyphenyl)-1′,3′-dimethyl-2′,4,4′-trioxo-1′,2′,3′,4′-tetrahydro-4*H*-spiro[indeno[1,2-*e*]pyrido[1,2-*b*][1,2,4]triazine-7,5′-pyrano[2,3-*d*]pyrimidine]-1,3,6′-tricarbonitrile (6k)

Orange powder, 82%, mp: 268–270 °C; IR (KBr): 3339, 2226, 1683, 1641, 1598, 1464, 1388, 1264, 1206, 1145, 1022, 857, 767 cm^−1^; ^1^H NMR (300 MHz, DMSO-*d*_6_) *δ*: 8.19 (1H, *d*, ^3^*J*_HH_ = 6.0 Hz, ArH), 8.08 (2H, s, NH_2_), 7.86–7.97 (2H, m, ArH), 7.70–7.74 (1H, m, ArH), 7.21–7.25 (3H, m, ArH), 3.86 (3H, s, OCH_3_), 3.80 (3H, s, OCH_3_), 3.44 (3H, s, NCH_3_), 2.91 (3H, s, NCH_3_); ^13^C NMR (75.4 MHz, DMSO-*d*_6_) *δ*: 162.5 (CONMe), 160.6 (CONN), 160.2 (CNH_2_), 160.0 (CO(NMe)_2_), 158.6 (CNMe), 156.3 (CNN), 155.6 (C=N), 152.9 (C=N–N), 150.0 (*C*C=N), 151.8, 151.3, 148.8, 138.8, 131.9, 131.4, 126.5, 125.9, 124.8, 122.2, 116.0, 112.7 (Ar), 117.0, 115.0, 112.1 (3CN), 97.9 (*C*–CN), 90.9 (*C*–CONMe), 86.9 (*C*CON), 56.9 (*C*=CNH_2_), 56.1 (OCH_3_), 48.2 (C_spiro_), 30.1 (NCH_3_), 28.1 (NCH_3_).

#### 2-Amino-2′-(4-(dimethylamino)phenyl)-4′,5-dioxo-5,6,7,8-tetrahydro-4′*H*-spiro[chromene-4,7′-indeno[1,2-*e*]pyrido[1,2-*b*][1,2,4]triazine]-1′,3,3′-tricarbonitrile (6l)

Brown powder; 81%, mp: 250–252 °C; IR (KBr): 3387, 3311, 3206, 2922, 2216, 2191, 1674, 1601, 1524, 1457, 1367, 1307, 1157, 1070, 949, 821, 770 cm^−1^; ^1^H NMR (300 MHz, DMSO-*d*_6_) *δ*: 8.17 (1H, *d*, ^3^*J*_HH_ = 6.5 Hz, ArH), 7.89–7.94 (1H, m, ArH), 7.75–7.79 (1H, m, ArH), 7.75 (2H, s, NH_2_), 7.67–7.72 (1H, m, ArH), 7.50 (2H, *d*, ^3^*J*_HH_ = 8.0 Hz, ArH), 6.88 (2H, *d*, ^3^*J*_HH_ = 8.0 Hz, ArH), 3.03 (6H, s, NMe_2_), 2.78–2.81 (2H, m, COCH_2_), 2.17–2.21 (2H, m, CH_2_), 1.91–1.95 (2H, m, CH_2_); ^13^C NMR (75.4 MHz, DMSO-*d*_6_) *δ*: 196.1 (*C*OCH_2_), 167.9 (CONN), 162.6 (CNH_2_), 160.6 (CNN), 160.0 (C=N), 159.1 (C=N–N), 156.6 (=*C*–O), 156.1 (C-4), 151.7 (*C*C=N), 152.3, 138.7, 131.7, 131.0, 130.7, 126.0, 124.8, 119.9, 117.6 (Ar), 116.6, 115.5, 111.9 (3CN), 111.7 (*C*–CN), 96.7 (*C*–COCH_2_), 90.3 (*C*CON), 56.7 (*C*=CNH_2_), 47.6 (C_spiro_), 36.6 (NMe_2_), 36.5 (O=C–*C*H_2_), 27.3 (=C–*C*H_2_), 20.0 (CH_2_).

#### 13-Methyl-14-(methylthio)-15-nitro-4,7-dioxo-2-phenyl-4,12-dihydro-6*H*,7*H*-11*b*,6*a*-(epiminoetheno)indeno[1,2-*e*]pyrido[1,2-*b*][1,2,4]triazine-1,3-dicarbonitrile (8a)

Creamy powder; 91%, mp: 262–263 °C; IR (KBr): 3441, 3170, 2224, 1729, 1674, 1544, 1388, 1340, 1248, 1064, 981, 804, 743 cm^−1^; ^1^H NMR (300 MHz, DMSO-*d*_6_) *δ*: 9.64 (1H, br s, NH), 8.59 (1H, *d*, ^3^*J*_HH_ = 6.8 Hz, ArH), 8.25 (1H, s, NH), 7.96–8.01 (2H, m, ArH), 7.71–7.76 (1H, m, ArH), 7.49–7.57 (5H, m, ArH), 3.40 (3H, s, NCH_3_), 2.61 (3H, s, SCH_3_); ^13^C NMR (75.4 MHz, DMSO-*d*_6_) *δ*: 191.3 (C=O), 164.3 (=CNNH), 160.2 (C–SMe), 157.5 (O=C–N), 155.6 (=C-3), 149.5, 137.7, 134.4, 133.9, 131.1, 131.1, 129.1, 129.1, 128.6, 125.5 (ArH), 124.3 (C–NO_2_), 116.1, 114.7, (2CN), 92.1 (NH–*C*–CO), 87.9 (*C*–NMe), 78.5 (*C*–CN), 65.8 (*C*–CN), 31.4 (NCH_3_), 17.7 (SCH_3_); MS (EI, 70 eV): *m*/*z* (%) = 523 (4) [M]^+^, 477 (65), 429 (100), 375 (23), 288 (84), 195 (43), 148 (49), 84 (61), 55 (73).

#### 2-(4-Bromophenyl)-13-methyl-14-(methylthio)-15-nitro-4,7-dioxo-4,12-dihydro-6*H*,7*H*-11*b*,6*a*-(epiminoetheno)indeno[1,2-*e*]pyrido[1,2-*b*][1,2,4]triazine-1,3-dicarbonitrile (8b)

Light orange powder; 87%, mp: 267–269 °C; IR (KBr): 3444, 3252, 2223, 1729, 1661, 1589, 1470, 1329, 1249, 1179, 1068, 901, 840, 759 cm^−1^; ^1^H NMR (300 MHz, DMSO-*d*_6_) *δ*: 9.76 (1H, br s, NH), 8.60 (1H, *d*, ^3^*J*_HH_ = 9 Hz, ArH), 8.27 (1H, s, NH), 7.96–8.01 (3H, m, ArH), 7.75–7.80 (3H, m, ArH), 7.45–7.48 (1H, m, ArH), 3.39 (3H, s, NCH_3_), 2.61 (3H, s, SCH_3_). ^13^C NMR (75.4 MHz, DMSO-*d*_6_) *δ*: 191.3 (C=O), 164.4 (=CNNH), 159.2 (C–SMe), 157.4 (O=C–N), 155.7 (=C-3), 149.4, 137.7, 133.9, 133.6, 132.3, 131.1, 130.8, 129.3, 125.5, 124.8 (ArH), 124.3 (C–NO_2_), 116.0, 114.6 (2CN), 92.0 (NH–*C*–CO), 87.9 (*C*–NMe), 78.4 (*C*–CN), 65.8 (*C*–CN), 31.5 (NCH_3_), 17.7 (SCH_3_); MS (EI, 70 eV): *m*/*z* (%) = 557 (7), 509 (12), 427 (3), 376 (2), 288 (19), 195 (12), 148 (64), 101 (76), 77 (24), 55 (100).

#### 2-(4-Chlorophenyl)-13-methyl-14-(methylthio)-15-nitro-4,7-dioxo-4,12-dihydro-6*H*,7*H*-11*b*,6*a*-(epiminoetheno)indeno[1,2-*e*]pyrido[1,2-*b*][1,2,4]triazine-1,3-dicarbonitrile (8c)

Light orange powder; 85%, mp: 265–267 °C; IR (KBr): 3238, 2221, 1651, 1565, 1469, 1336, 1235, 1143, 1093, 971, 843, 766 cm^−1^; ^1^H NMR (300 MHz, DMSO-*d*_6_) *δ*: 9.92 (1H, br s, NH), 8.42 (1H, s, NH), 7.98 (1H, *d*, ^3^*J*_HH_ = 6 Hz, ArH), 7.81–7.88 (2H, m, ArH), 7.69–7.74 (1H, m, ArH), 7.63–7.66 (4H, m, ArH), 3.56 (3H, s, NCH_3_), 2.61 (3H, s, SCH_3_). ^13^C NMR (75.4 MHz, DMSO-*d*_6_) *δ*: 191.1 (C=O), 164.2 (=CNNH), 159.7 (C–SMe), 155.7 (O=C–N), 155.2 (=C-3), 150.0, 142.4, 135.8, 135.3, 133.4, 132.5, 130.6, 129.3, 125.7, 123.5 (ArH), 123.1 (C–NO_2_), 116.2, 115.2, (2CN), 98.3 (NH–*C*–CO), 89.8 (*C*–NMe), 74.9 (*C*–CN), 61.6 (*C*–CN), 31.7 (NCH_3_), 17.6 (SCH_3_).

#### 2-(4-Fluorophenyl)-13-methyl-14-(methylthio)-15-nitro-4,7-dioxo-4,12-dihydro-6*H*,7*H*-11*b*,6*a*-(epiminoetheno)indeno[1,2-*e*]pyrido[1,2-*b*][1,2,4]triazine-1,3-dicarbonitrile (8d)

Light orange powder; 79%, mp: 266–268 °C; ^1^H NMR (300 MHz, DMSO-*d*_6_) *δ*: 9.89 (1H, br s, NH), 8.42 (1H, s, NH), 7.98 (1H, *d*, ^3^*J*_HH_ = 6.8 Hz, ArH), 7.83–7.86 (2H, m, ArH), 7.63–7.68 (3H, m, ArH), 7.38–7.44 (3H, m, ArH), 3.56 (3H, s, NCH_3_), 2.61 (3H, s, SCH_3_). ^13^C NMR (75.4 MHz, DMSO-*d*_6_) *δ*: 191.6 (C=O), 165.3 (=CNNH), 164.1 (C–F, *d*, ^1^*J*_CF_ = 246.0 Hz), 159.9 (C–SMe), 155.7 (O=C–N), 155.2 (=C-3), 150.0, 142.4, 141.0, 138.5, 132.5, 131.5, 131.3 (*d*, ^3^*J*_CF_ = 8.2 Hz), 125.6 (ArH), 123.5 (C–NO_2_), 116.7 (*d*, ^2^*J*_CF_ = 22.5 Hz, Ar), 116.4, 116.1 (2CN), 98.4 (NH–*C*–CO), 91.8 (*C*–NMe), 75.1 (*C*–CN), 61.7 (*C*–CN), 31.7 (NCH_3_), 17.6 (SCH_3_).

## Conflicts of interest

The authors declare no competing financial interest.

## Supplementary Material

RA-013-D3RA06248A-s001

## References

[cit1] Kotb E. R., Anwar M. M., Syam Y. M., Bagato O., Abdel Moaz S. (2015). Int. J. Pharm. Technol..

[cit2] Cascioferro S., Parrino B., Spano V., Carbone A., Montalbano A., Barraja P., Diana P., Cirrincione G. (2017). Eur. J. Med. Chem..

[cit3] Ibrahim M. A., Abdel-Rahman R. M., Abdel-Halim A. M., Ibrahim S. S., Allimony H. A. (2009). J. Braz. Chem. Soc..

[cit4] Mullick P., Khan S. A., Begum T., Verma S., Kaushik D., Alam O. (2009). Acta Pol. Pharm. Drug Res..

[cit5] Arshad M., Bhat A. R., Hoi K. K., Choi I., Athar F. (2017). Chin. Chem. Lett..

[cit6] Serrar H., Marmouzi I., Benzekri Z., Boukhris S., Hassikou A., Faouzi M. E., Souizi A. (2017). Lett. Org. Chem..

[cit7] Kumar R., Sirohi T. S., Singh H., Yadav R., Roy R. K., Chaudhary A., Pandeya S. N. (2014). Mini-Rev. Med. Chem..

[cit8] Khanzadeh M., Dehghanipour M., Darehkordi A., Rahmani F. (2018). Can. J. Phys..

[cit9] ELaoufir Y., Bourazmi H., Serrar H., Zarrok H., Zarrouk A., Hammouti B., Guenbour A., Boukhriss S., Oudda H. (2014). Pharm. Lett..

[cit10] Phucho T., Nongpiur A., Tumtin S., Nongrum R., Myrboh B., Nonghlaw R. L. (2008). Arkivoc.

[cit11] Liu Y., Guo X., Tang D., Wang J., Wu P., Han J., Chen B. (2017). Chin. J. Chem..

[cit12] Ibrahim H. M., Behbehani H. (2021). ACS Omega.

[cit13] Ibrahim M. A., El-Gohary N. M. (2014). Heterocycles.

[cit14] Darehkordi A., Hosseini M., Rahmani F. (2019). J. Heterocycl. Chem..

[cit15] Darehkordi A., Salehi V., Rahmani F., Karimipour M. (2018). Chem. Heterocycl. Compd..

[cit16] Tahmasby M., Darehkordi A., Mohammadi M., Nejadkhorasani F. (2021). J. Mol. Struct..

[cit17] Trabocchi A. , Diversity-Oriented Synthesis: Basics and Applications in Organic Synthesis, Drug Discovery, and Chemical Biology, Wiley and Sons, 2013

[cit18] Schreiber L. (2000). Science.

[cit19] Lenci E., Trabocchi A. (2022). Eur. J. Org Chem..

[cit20] Ding A., Meazza M., Guo H., Yang J. W., Rios R. (2018). Chem. Soc. Rev..

[cit21] Suthar M., Kumbhani J., Bhatt K. D. (2021). Orient. J. Chem..

[cit22] Saigal, Irfan M., Khan P., Abid M., Khan M. M. (2019). ACS Omega.

[cit23] Akbari A., Azami-Sardooei Z., Hosseini-Nia A. (2013). J. Korean Chem. Soc..

[cit24] Rodrigues M. O., Eberlin M. N., Neto B. A. D. (2021). Chem. Rec..

[cit25] Alvim H. G. O., da Silva Júnior E. N., Neto B. A. D. (2014). RSC Adv..

[cit26] Tashrifi Z., Mohammadi-Khanaposhtani M., Hamedifar H., Larijani B., Ansari S., Mahdavi M. (2020). Mol. Diversity.

[cit27] Borah B., Dwivedi K. D., Chowhan L. R. (2022). Polycyclic Aromat. Compd..

[cit28] Guo R. Y., An Z. M., Mo L. P., Wang R. Z., Li H. X., Wang S. X., Zhang Z. H. (2013). ACS Comb. Sci..

[cit29] He Y., Guo H., Tian J. (2011). J. Chem. Res..

[cit30] Saluja P., Aggarwal K., Khurana J. M. (2013). Synth. Commun..

[cit31] Khanna P., Khanna L., Thomas S. J., Asiri A. M., Panda S. S. (2018). Curr. Org. Chem..

[cit32] Chen F., Zheng J., Huang M., Li Y. (2015). Res. Chem. Intermed..

[cit33] Bayat M., Hosseini H. (2017). New J. Chem..

[cit34] Sadeghian Z., Bayat M., Safari F. (2021). Med. Chem. Res..

[cit35] Safari F., Hosseini H., Bayat M., Ranjbar A. (2019). RSC Adv..

[cit36] Sadeghian Z., Bayat M., Safari F. (2022). J. Mol. Struct..

[cit37] Sadeghian Z., Bayat M., Safari F. (2022). Med. Chem. Res..

[cit38] Ezzatzadeh E., Soleimani-Amiri S., Hossaini Z., Khandan Barani K. (2022). Front. Chem..

[cit39] Dilmaç A. M., Wezeman T., Bär R. M., Bräse S. (2020). Nat. Prod. Rep..

[cit40] Chen N., Xia S., Zou M., Shao X. (2015). Res. Chem. Intermed..

[cit41] Yadav M. B., Bhosle S. R., Jeong Y. T. (2022). Tetrahedron Lett..

[cit42] Kato K., Tanaka S., Seto N., Wada K., Gon M., Fa S., Ohtani S., Tanaka K., Ogoshi T. (2023). Chem. Commun..

[cit43] Yavari I., Khajeh-Khezri A., Halvagar M. R. (2018). Arabian J. Chem..

[cit44] Yavari I., Malekafzali A., Skoulika S. (2014). Tetrahedron Lett..

[cit45] Khan S., Rahman H., Khan M. M. (2019). RSC Adv..

[cit46] Abedinifar F., Larijani B., Mahdavi M. (2022). RSC Adv..

[cit47] Hosseini H., Bayat M. (2018). RSC Adv..

[cit48] Shokoohian M., Hazeri N., Maghsoodlou M. T., Lashkari M. (2022). Polycyclic Aromat. Compd..

