# Supplementary material for: Efficient synthesis of new indenopyridotriazine [4.3.3]propellanes and spiroindenopyridotriazine-4H-pyran derivatives
Source: RSC Adv. 2023 Oct 27;13(45):31488–96. doi: 10.1039/d3ra06248a (PMC10604634; doi:10.1039/d3ra06248a)
Supplement: RA-013-D3RA06248A-s001 [file RA-013-D3RA06248A-s001.pdf]

Electronic Supplementary Material (ESI) for RSC Advances.
This journal is © The Royal Society of Chemistry 2023

Supporting Information

Efficient synthesis of new indenopyridotriazine [4.3.3]propellanes and spiroindenopyridotriazine-4*H*-pyran derivatives

Monireh Rezaei,[a] and Mohammad Bayat [a,*]

Department of science, Imam Khomeini International University, P.O. Box 14115-175, Qazvin, Iran

Table of Contents

Title	Page
Title, author's name, address and table of contents	1
General remarks	2
**Figure 1**. Structure of all products **6a-l**	3
**Figure 2**. Structure of all products **8a-d**	3
^{1}H and ^{13}C NMR and IR spectrums of **3a**	4-6
^{1}H and ^{13}C NMR and IR and Mass spectrums of **6a**	7-11
^{1}H and ^{13}C NMR and IR and Mass spectrums of **6b**	12-15
^{1}H and ^{13}C NMR and IR and Mass spectrums of **6c**	16-20
^{1}H and ^{13}C NMR and IR and Mass spectrums of **6d**	21-24
^{1}H NMR and IR and Mass spectrums of **6e**	25-27
^{1}H and ^{13}C NMR and IR and Mass spectrums of **6f**	28-31
^{1}H and ^{13}C NMR and IR and Mass spectrums of **6g**	32-35
^{1}H and ^{13}C NMR spectrums of **6h**	36-37
^{1}H and ^{13}C NMR spectrums of **6i**	38-40
^{1}H and ^{13}C NMR spectrums of **6j**	41-42
^{1}H and ^{13}C NMR and IR spectrums of **6k**	43-45
^{1}H and ^{13}C NMR and IR spectrums of **6l**	46-48
^{1}H and ^{13}C NMR and IR and Mass spectrums of **8a**	49-53
^{1}H and ^{13}C NMR and IR and Mass spectrums of **8b**	54-57
^{1}H and ^{13}C NMR and IR spectrums of **8c**	58-60
^{1}H and ^{13}C NMR spectrums of **8d**	61-62

Experimental Section

General remarks:

All commercially available reagents and other solvents were purchased from Aldrich and Merck Chemical Co. and used without further purification. The NMR spectra were recorded with a Bruker DRX-300 AVANCE instrument (300 MHz for ^{1}H and 75.4 MHz for ^{13}C) with DMSO-d_6 as solvent. Chemical shifts are given in ppm (*d*) relative to the internal TMS, and the coupling constant (*J*) reported in hertz (Hz). Melting points were measured with an electrotherma1 9100 apparatus. Mass spectra were recorded with an Agilent 5975C VL MSD with a Triple-Axis detector operating at an ionization potential of 70 eV. IR spectra were measured with a Bruker Tensor 27 spectrometer.

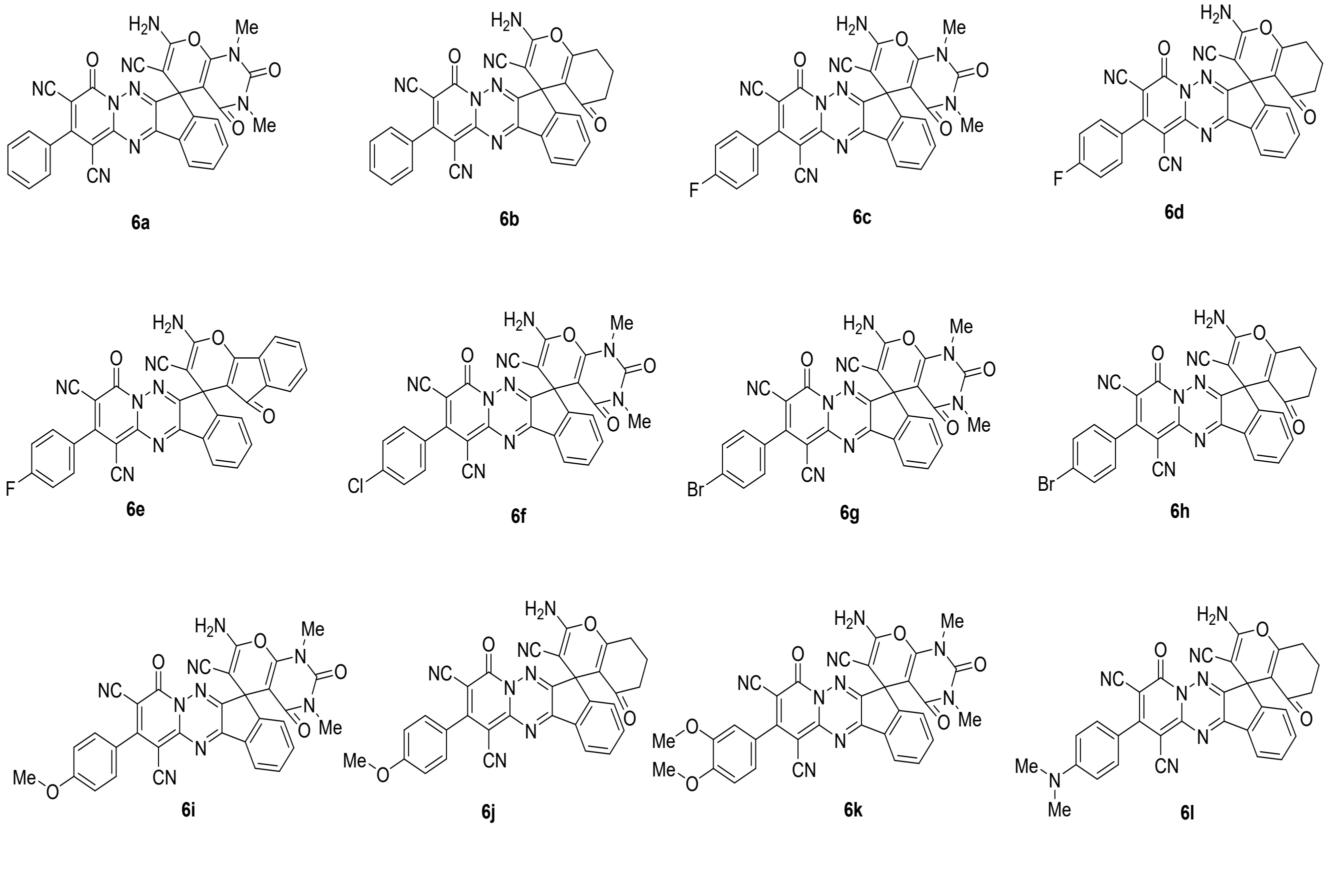

Figure 1. Structure of all products **6a-l**

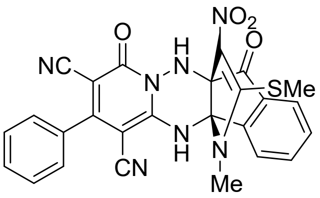

8a

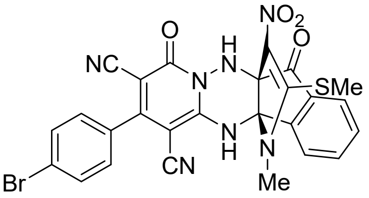

8b

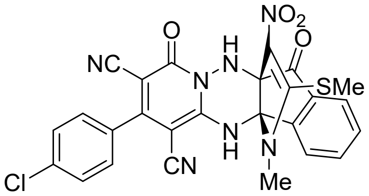

8c

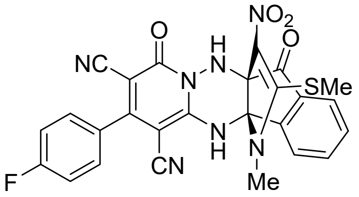

8d

Figure 2. Structure of all products **8a-d**

The structures all of the products **6a-l** and **8a-d** were deduced from their IR, ^{1}H NMR, ^{13}C NMR and Mass spectra (see the Supporting Information).

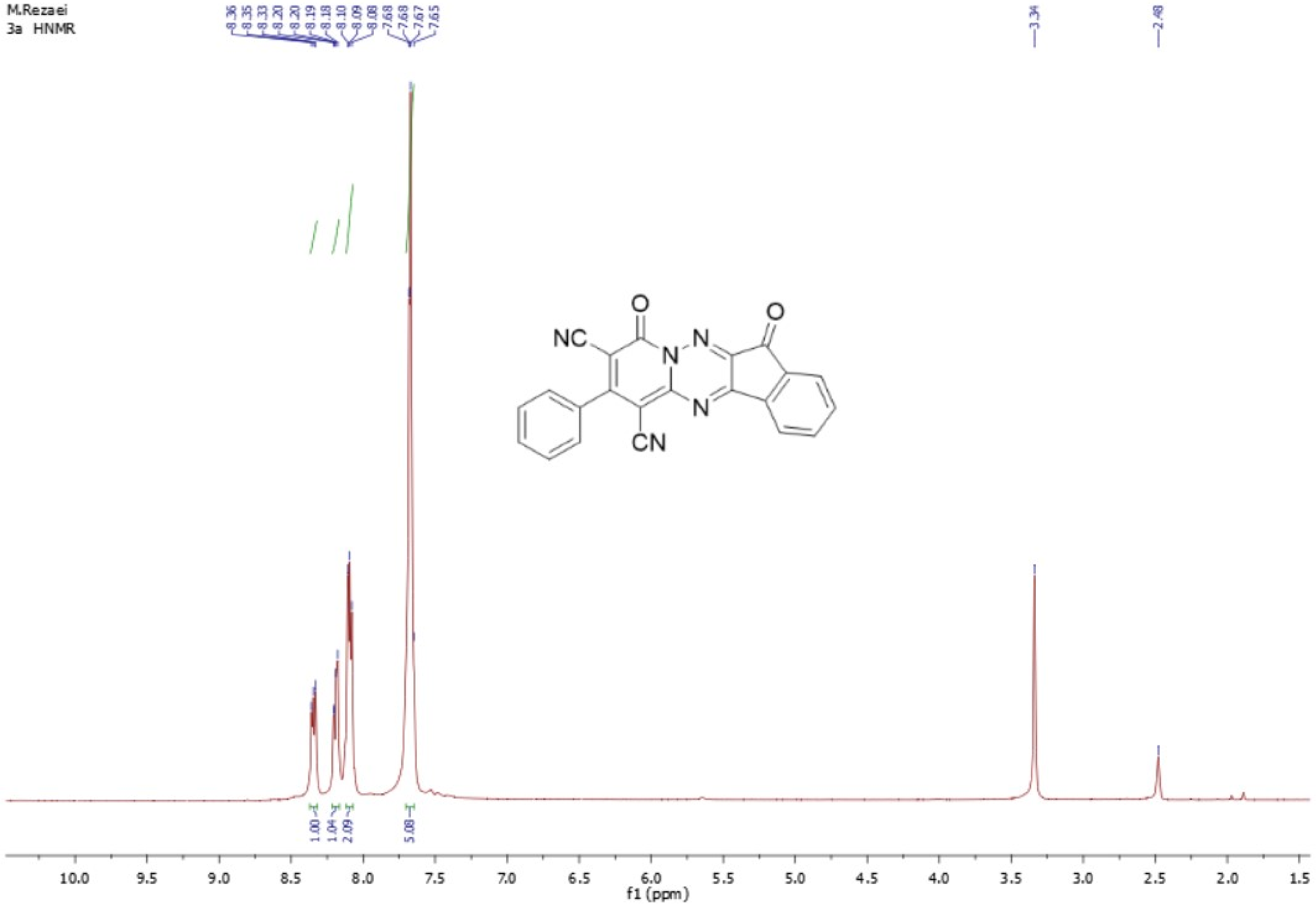

M.Rezaei
3a HNMR
8.36 8.35 8.33 8.20 8.19 8.18 8.10 8.09 8.08 7.68 7.68 7.67 7.65
3.34
2.48
1.00
1.04
2.09
5.08
NC
CN
O
N
10.0 9.5 9.0 8.5 8.0 7.5 7.0 6.5 6.0 5.5 5.0 4.5 4.0 3.5 3.0 2.5 2.0 1.5
f1 (ppm)

^{1}H NMR of 3a

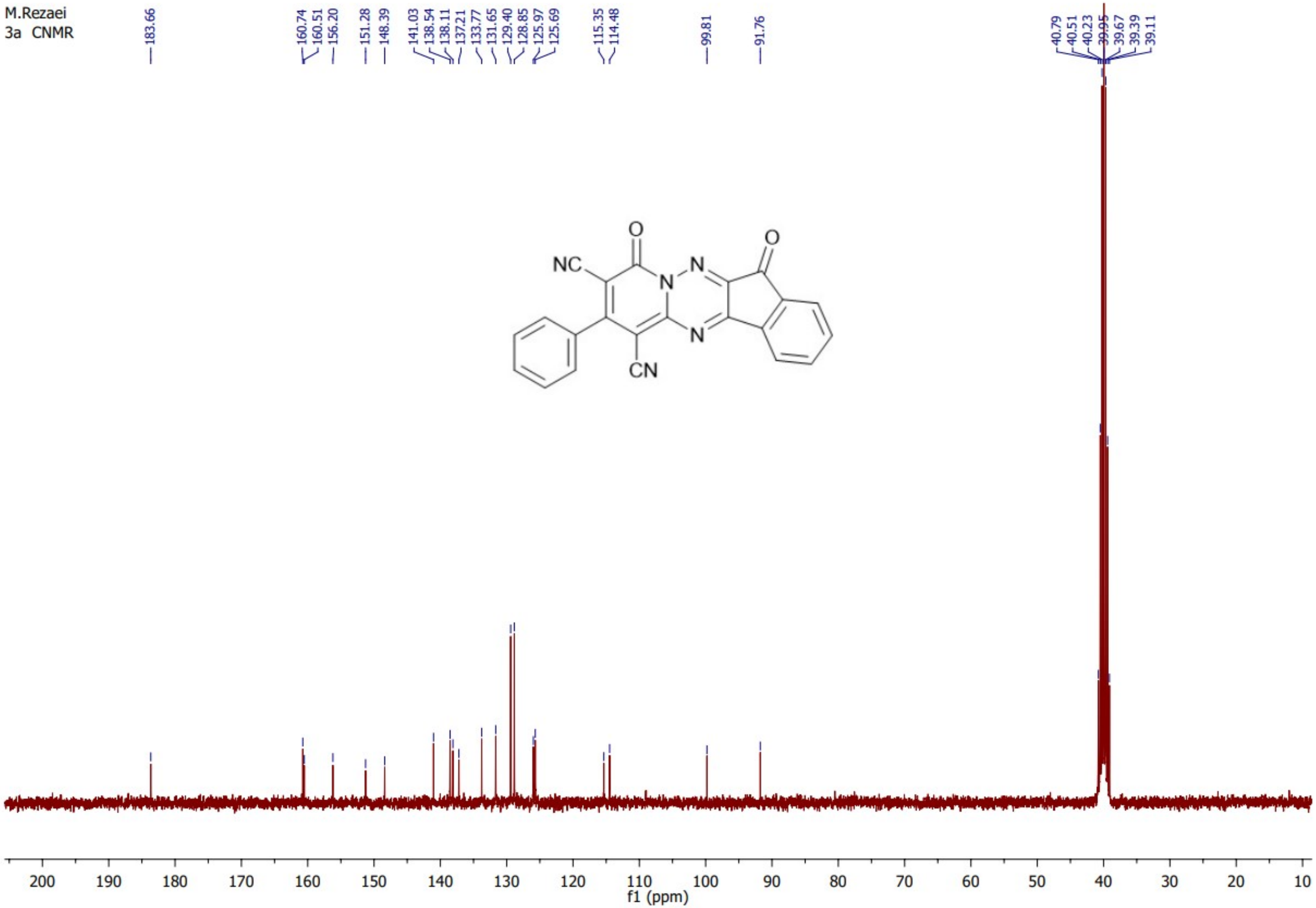

M.Rezaei
3a CNMR
183.66
160.74
160.51
156.20
151.28
148.39
141.03
138.54
138.11
137.21
133.77
131.65
129.40
128.85
125.97
125.69
115.35
114.48
99.81
91.76
40.79
40.51
40.23
39.95
39.67
39.39
39.11
NC
O
N
N
N
O
CN
200
190
180
170
160
150
140
130
120
110
100
90
80
70
60
50
40
30
20
10
f1 (ppm)

^{13}C NMR of 3a

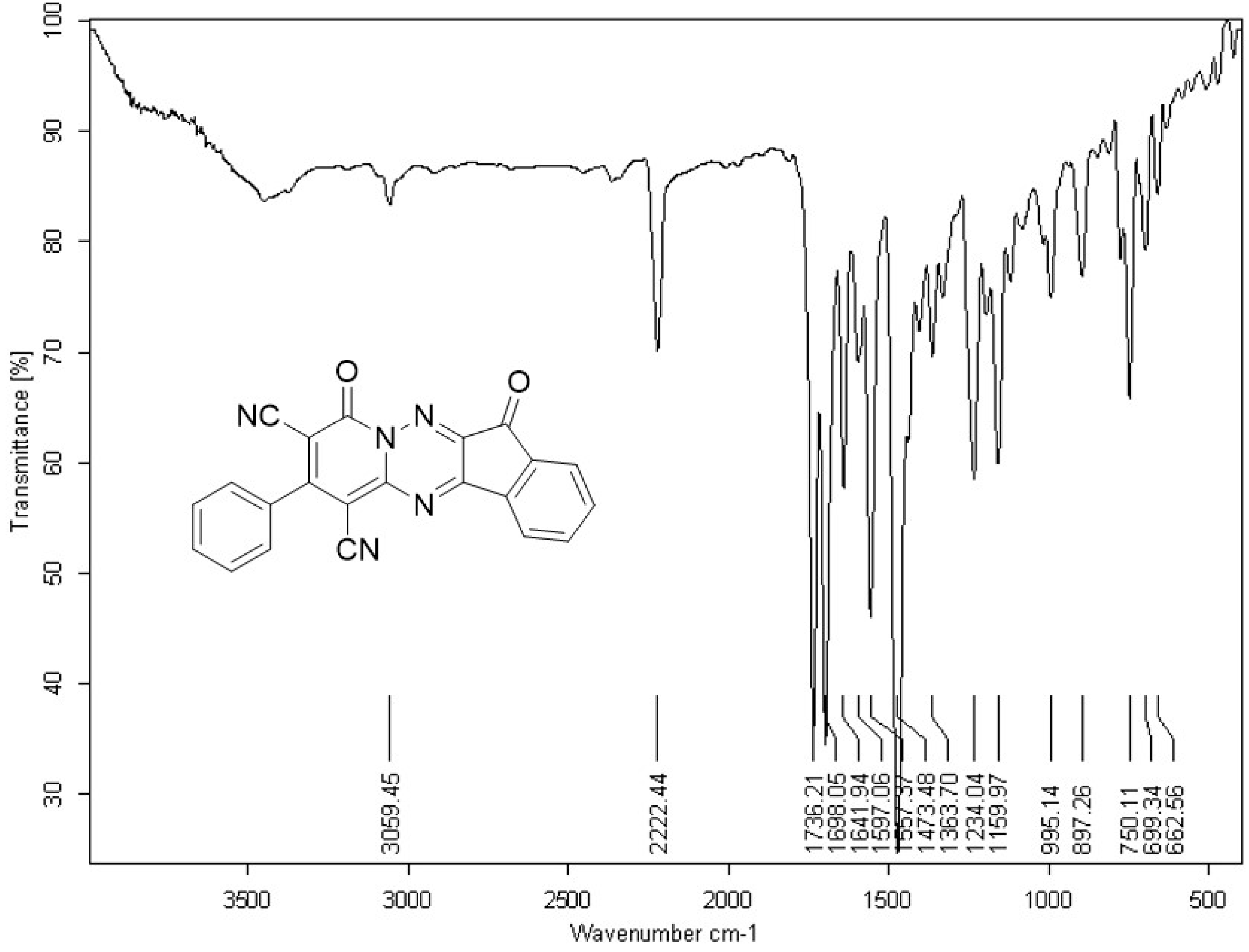

100
90
80
70
60
50
40
30
Transmittance [%]
3059.45
2222.44
1736.21
1698.05
1641.94
1597.06
1557.37
1473.48
1363.70
1234.04
1159.97
995.14
897.26
750.11
699.34
662.56
3500
3000
2500
2000
1500
1000
500
Wavenumber cm-1
NC
O
N
N
O
N
CN

IR of 3a

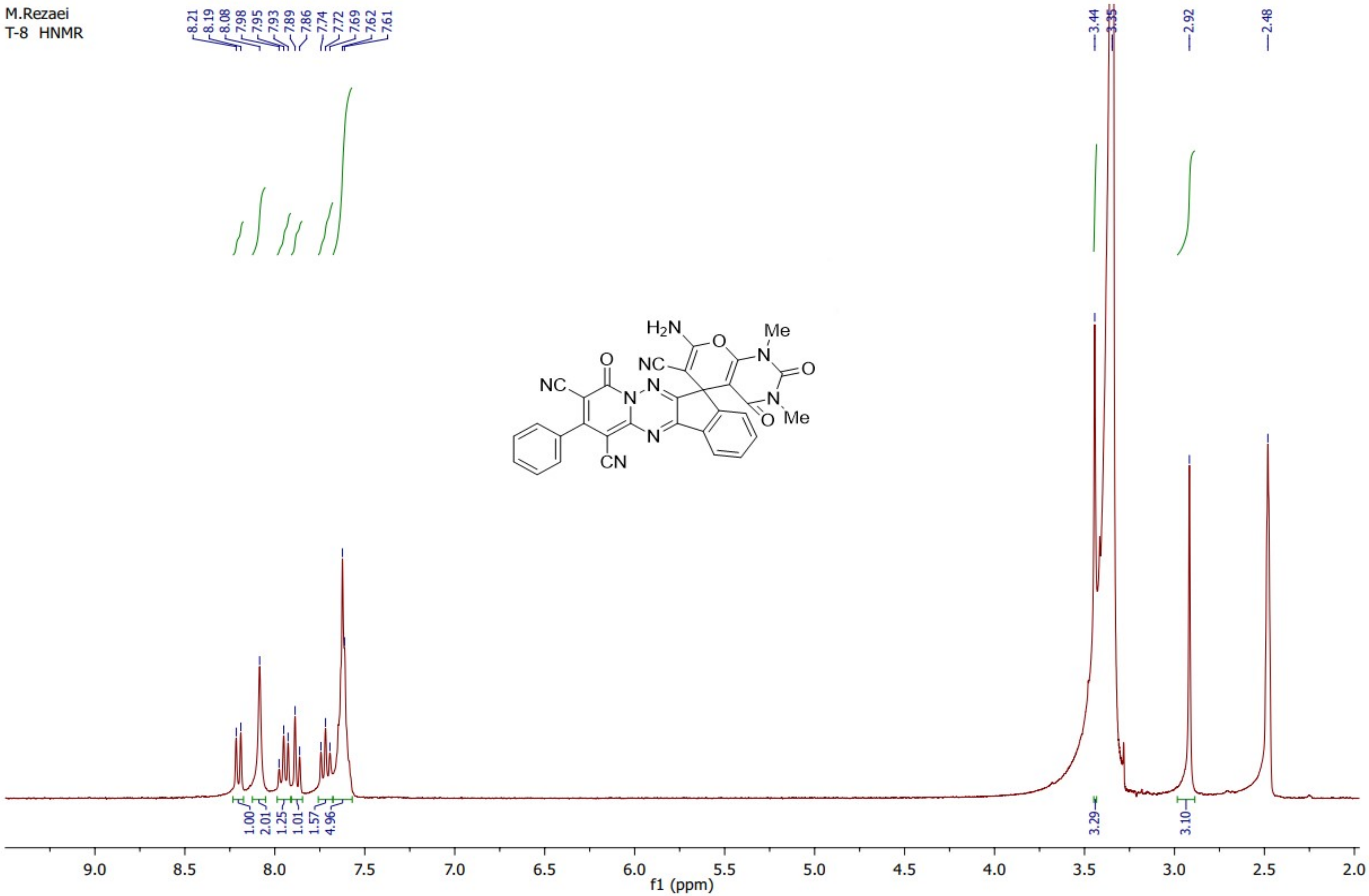
M.Rezaei
T-8 HNMR
8.21
8.19
8.08
7.98
7.95
7.93
7.89
7.86
7.74
7.72
7.69
7.62
7.61
3.44
3.35
2.92
2.48
1.00
2.01
1.25
1.01
1.57
4.96
3.29
3.10
H2N
O
Me
N
O
NC
O
NC
N
N
N
N
O
Me
N
CN
9.0
8.5
8.0
7.5
7.0
6.5
6.0
5.5
5.0
4.5
4.0
3.5
3.0
2.5
2.0
f1 (ppm)

^{1}H NMR of 6a

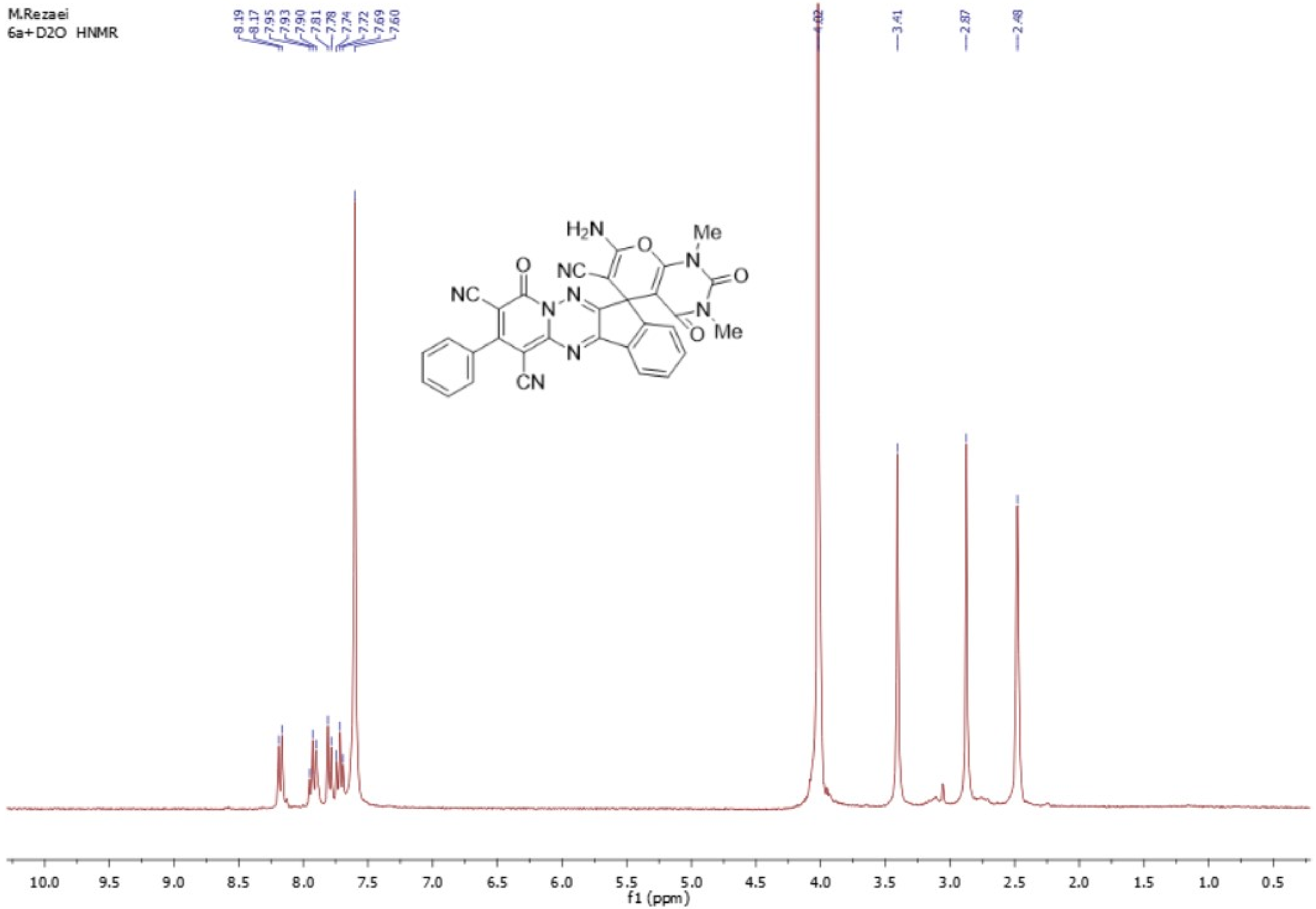

^{1}H NMR of 6a (D_2O exchangeable)

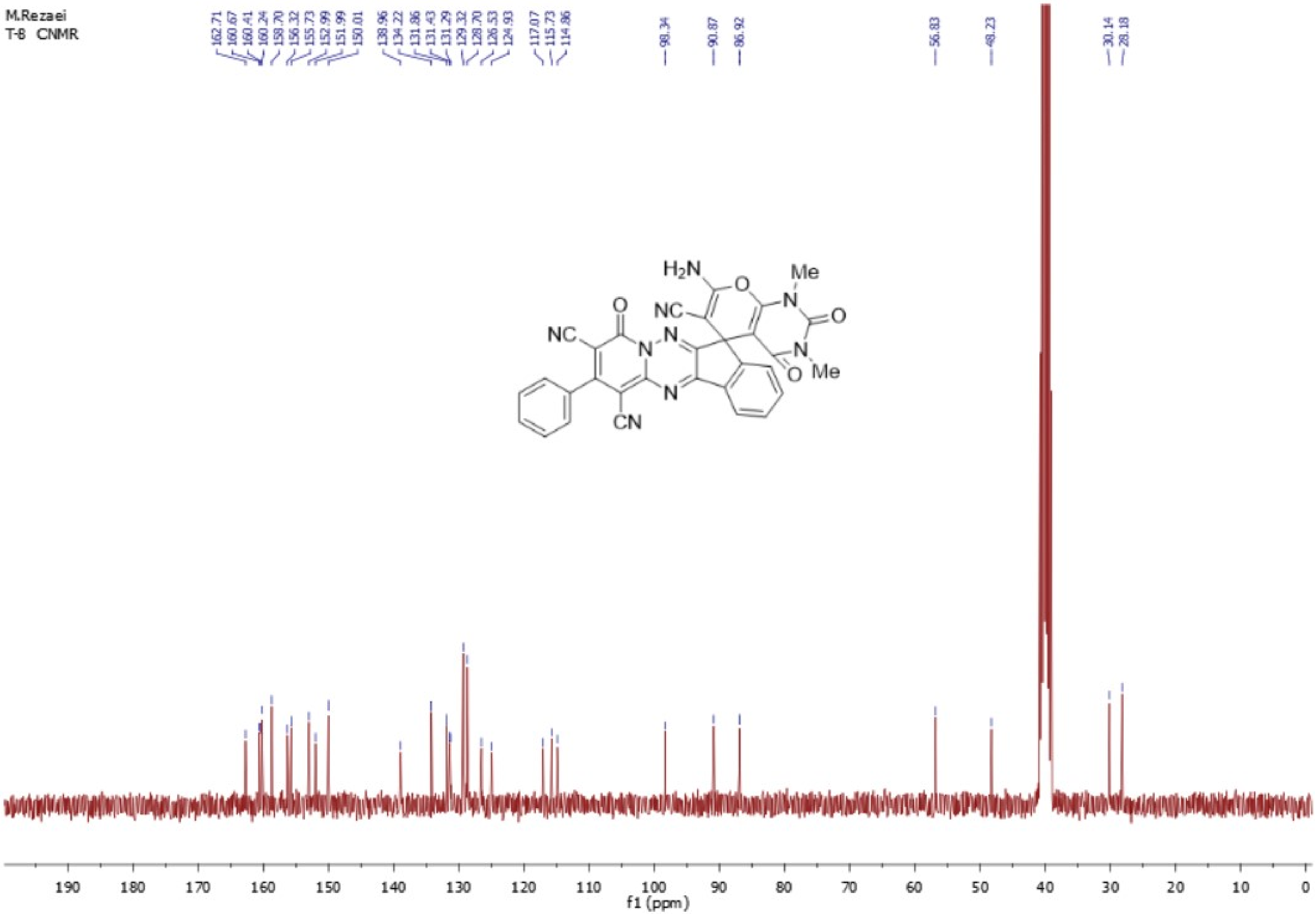
M.Rezaei
T-8 CNMR
162.71
160.67
160.41
160.24
158.70
156.32
155.73
152.99
151.99
150.01
138.96
134.22
131.86
131.43
131.29
129.32
128.70
126.53
124.93
117.07
115.73
114.86
98.34
90.87
86.92
56.83
48.23
30.14
28.18
H2N
O
Me
N
NC
O
NC
N
N
N
O
N
N
O
Me
CN
190 180 170 160 150 140 130 120 110 100 90 80 70 60 50 40 30 20 10 0
f1 (ppm)

^{13}C NMR of 6a

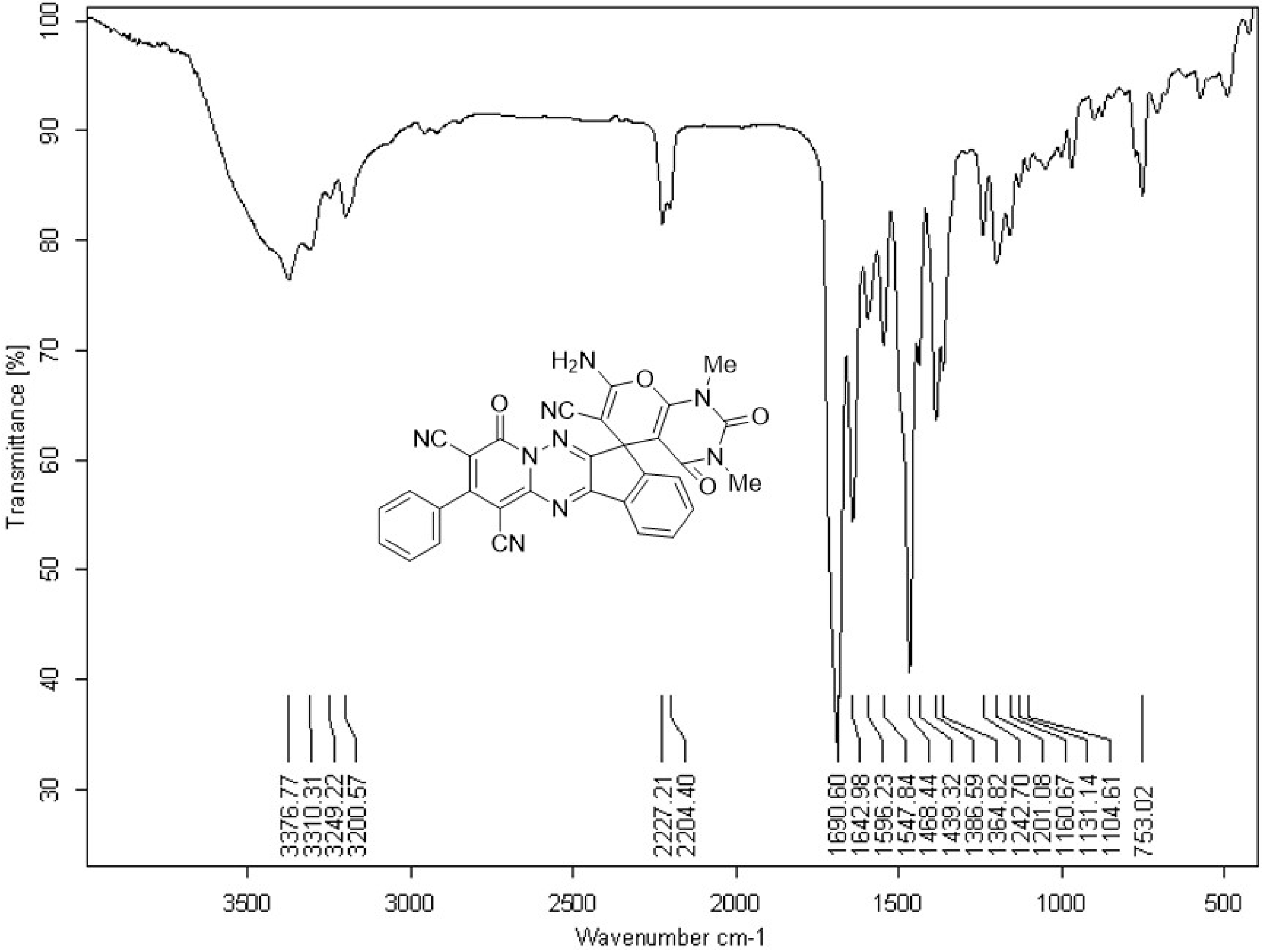
Transmittance [%]
Wavenumber cm-1
100
90
80
70
60
50
40
30
3500
3000
2500
2000
1500
1000
500
3376.77
3310.31
3249.22
3200.57
2227.21
2204.40
1690.60
1642.98
1596.23
1547.84
1468.44
1439.32
1386.59
1364.82
1242.70
1201.08
1160.67
1131.14
1104.61
753.02
H2N
O
Me
N
O
NC
O
NC
N
N
N
O
Me
N
CN

IR of 6a

File :C:\MSDCHEM\3\DATA\Snapshot\140110151.D
Operator :
Acquired : 2 Jan 2007 00:54 using AcqMethod test.M
Instrument : MSD
Sample Name: T8
Misc Info :
Vial Number: 1

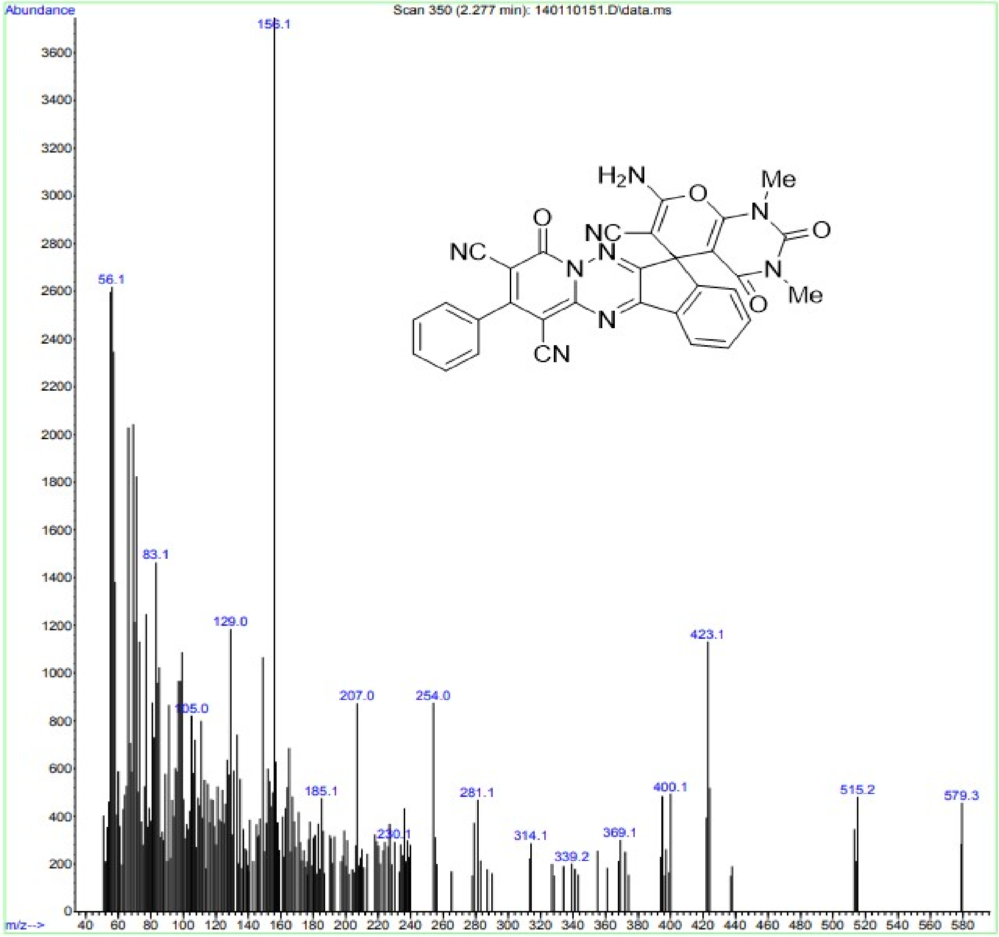

MS of 6a

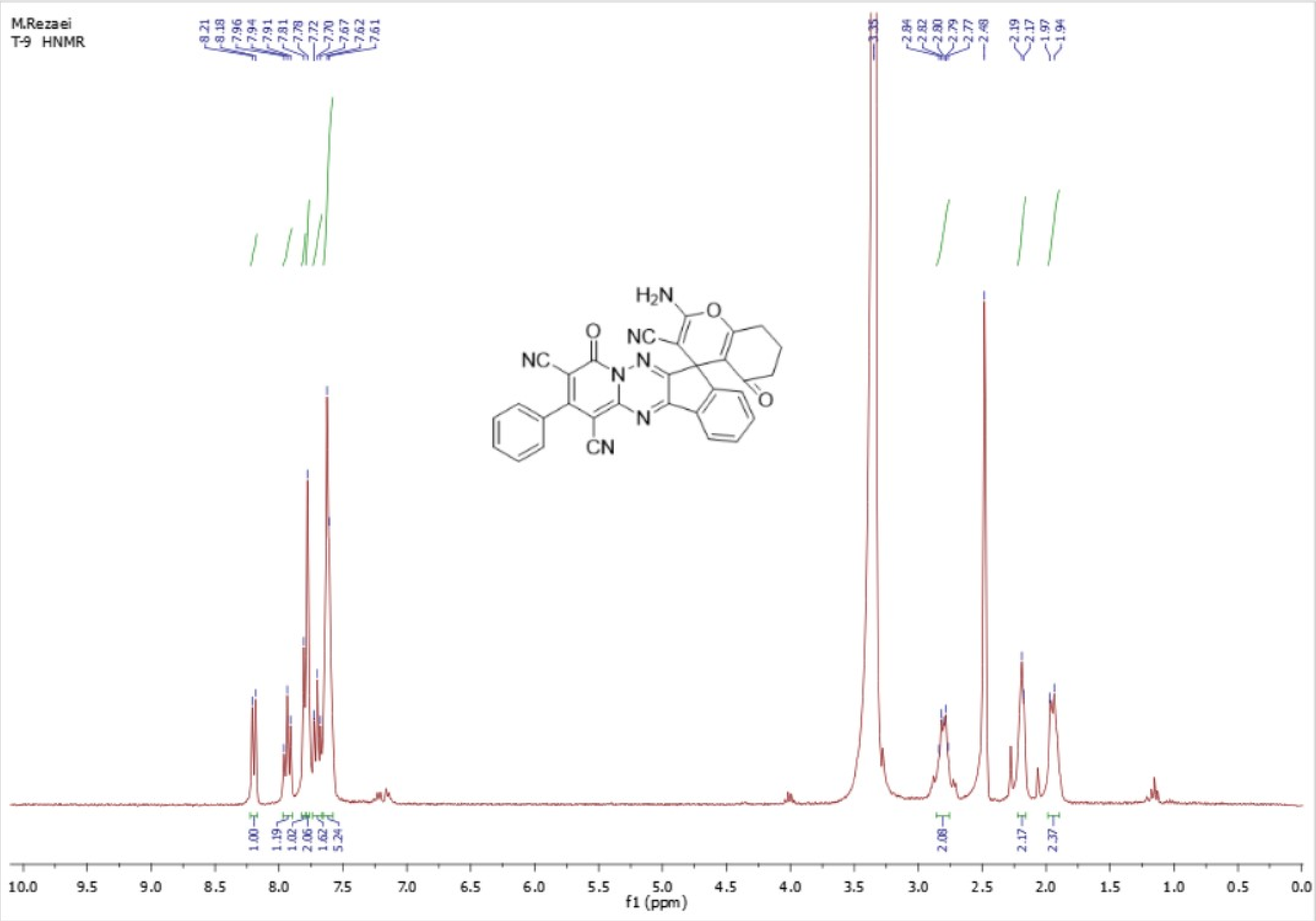
M.Rezaei
T-9 HNMR
8.21
8.18
7.96
7.94
7.91
7.81
7.78
7.72
7.70
7.67
7.62
7.61
3.35
2.84
2.82
2.80
2.79
2.77
2.48
2.19
2.17
1.97
1.94
H2N
O
NC
O
NC
N
N
N
O
N
CN
1.00
1.19
1.02
2.06
1.62
5.24
2.08
2.17
2.37
10.0
9.5
9.0
8.5
8.0
7.5
7.0
6.5
6.0
5.5
5.0
4.5
4.0
3.5
3.0
2.5
2.0
1.5
1.0
0.5
0.0
f1 (ppm)

^{1}H NMR of 6b

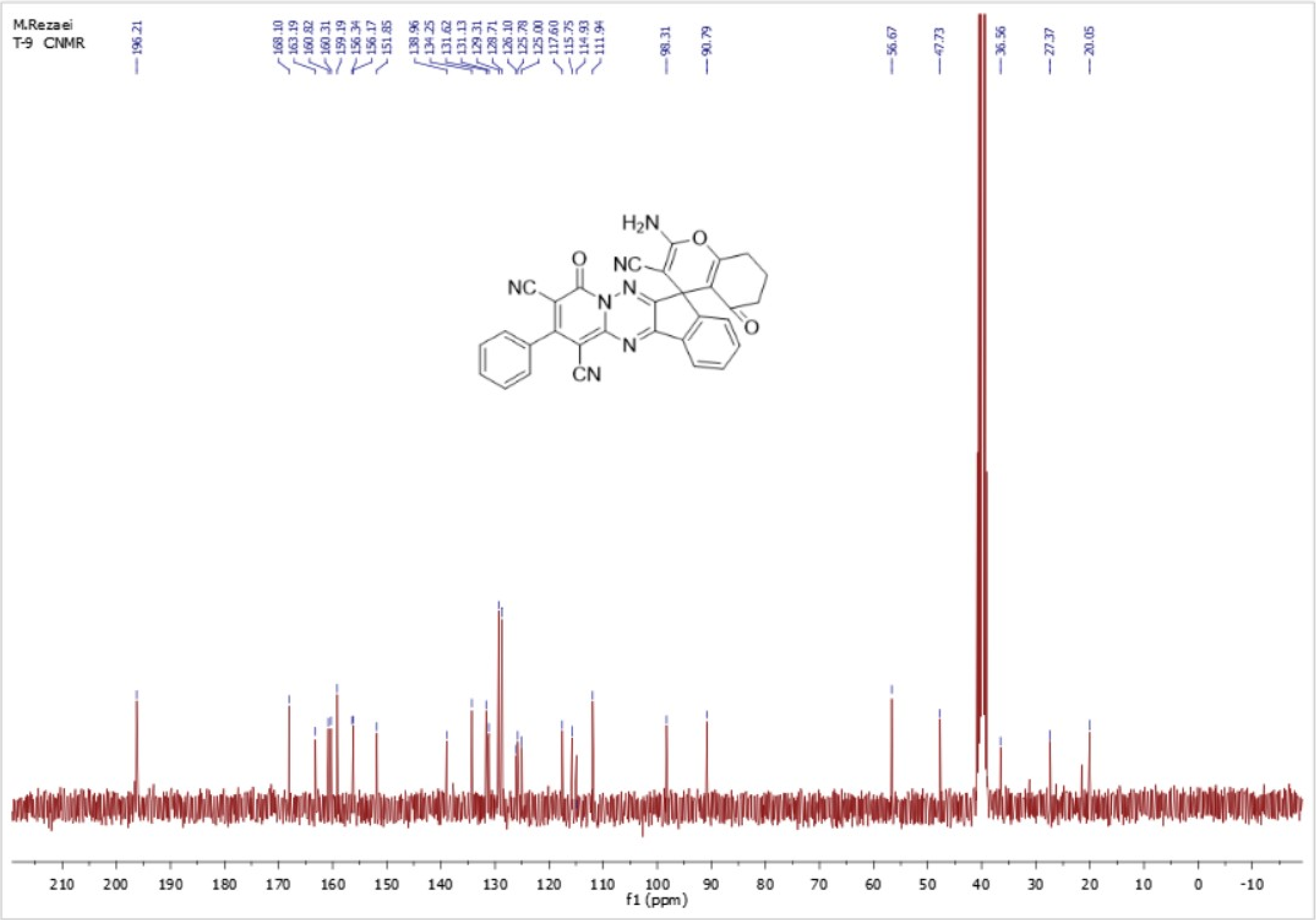
M.Rezaei
T-9 CNMR
196.21
168.10
163.19
160.82
160.31
159.19
156.34
156.17
151.85
138.96
134.25
131.62
131.13
129.31
128.71
126.10
125.78
125.00
117.60
115.75
114.93
111.94
98.31
90.79
56.67
47.73
36.56
27.37
20.05
f1 (ppm)

^{13}C NMR of 6b

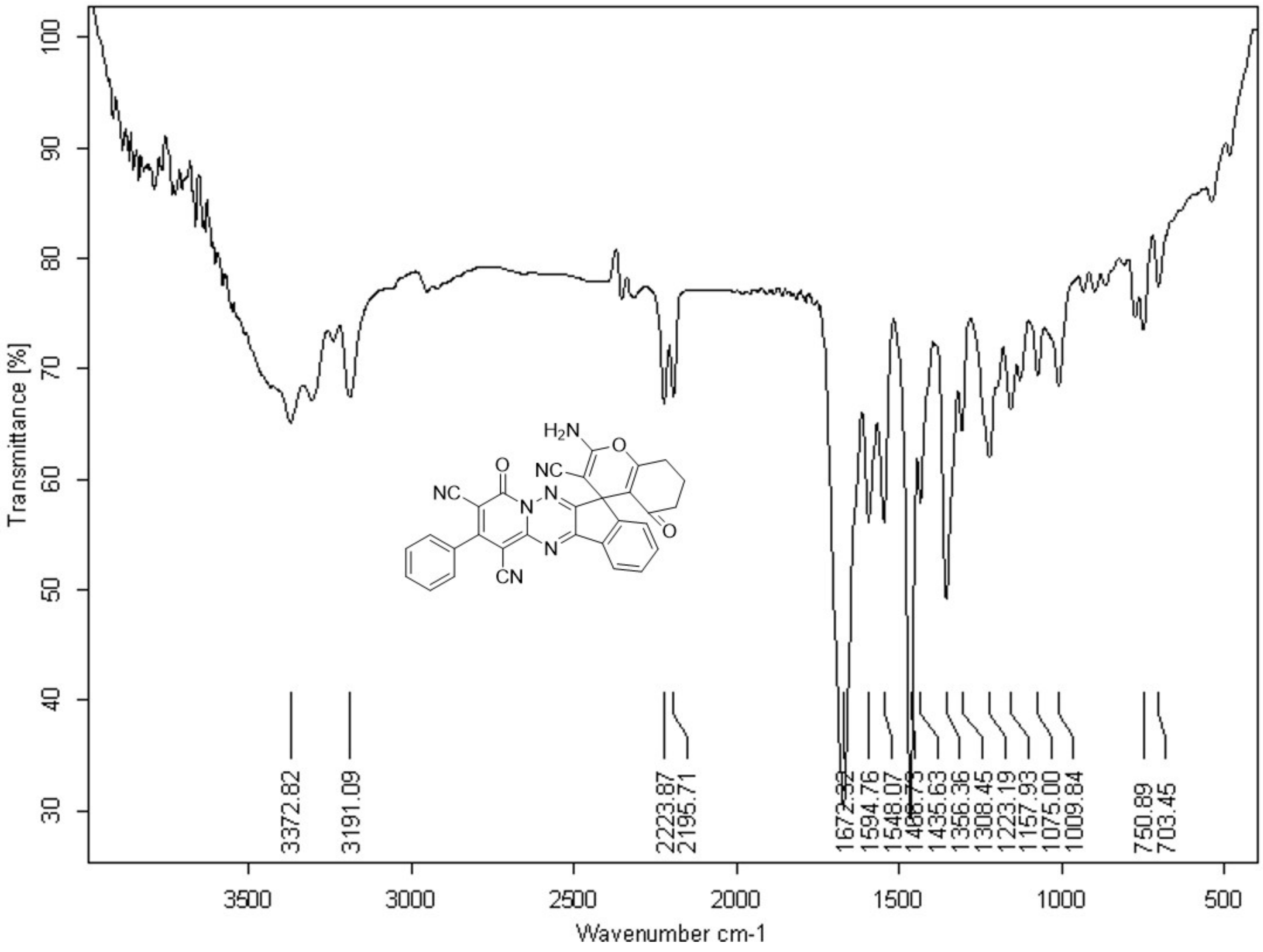
Transmittance [%]
Wavenumber cm-1
3372.82
3191.09
2223.87
2195.71
1672.92
1594.76
1548.07
1466.73
1435.63
1356.36
1308.45
1223.19
1157.93
1075.00
1009.84
750.89
703.45
H2N
NC
O
NC
N
CN

IR of 6b

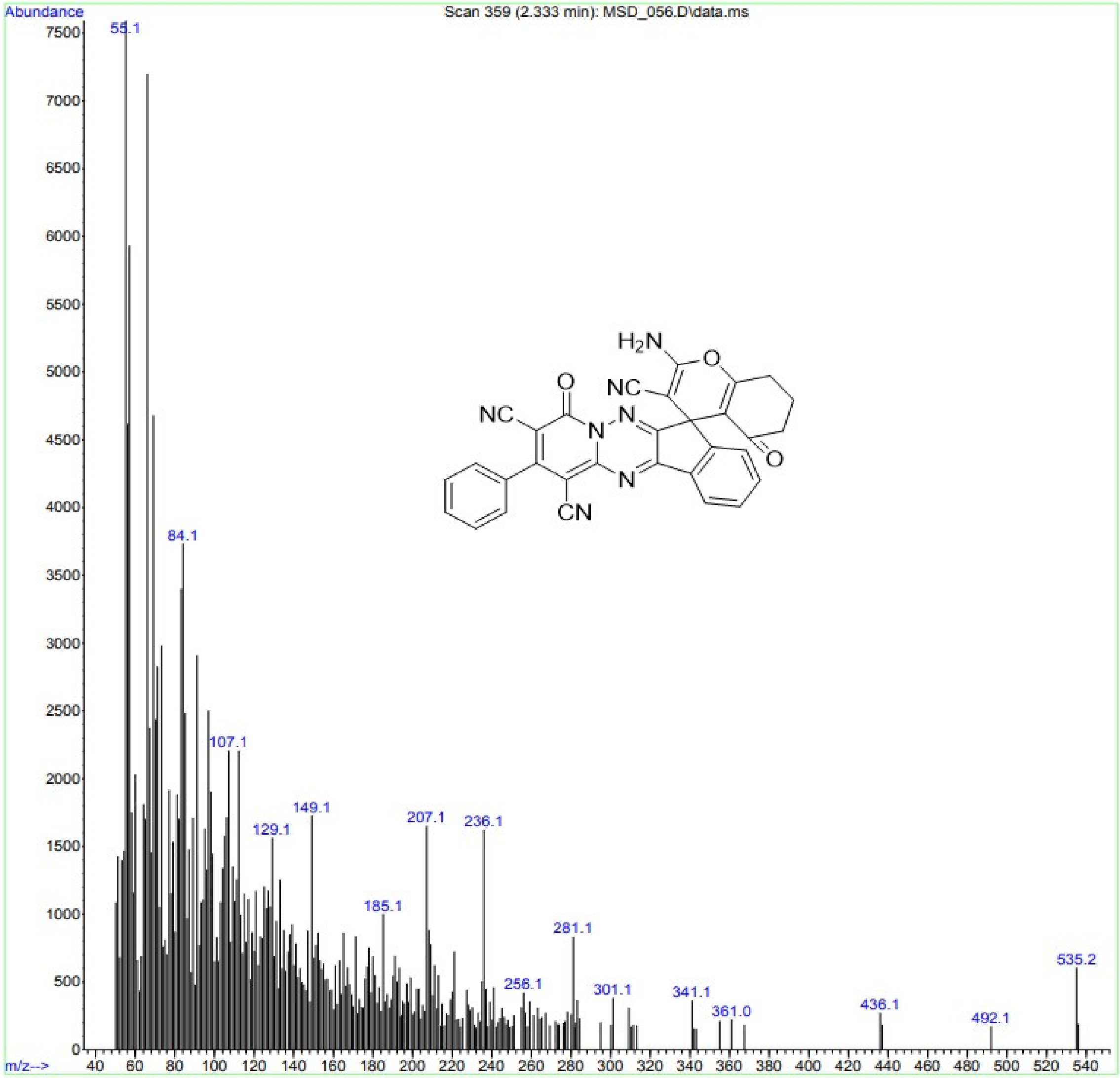

Abundance
Scan 359 (2.333 min): MSD_056.D\data.ms
7500
7000
6500
6000
5500
5000
4500
4000
3500
3000
2500
2000
1500
1000
500
0
55.1
84.1
107.1
129.1
149.1
185.1
207.1
236.1
256.1
281.1
301.1
341.1
361.0
436.1
492.1
535.2
m/z-->
40 60 80 100 120 140 160 180 200 220 240 260 280 300 320 340 360 380 400 420 440 460 480 500 520 540
H2N
O
NC
NC
O
N
N
N
CN
O

MS of 6b

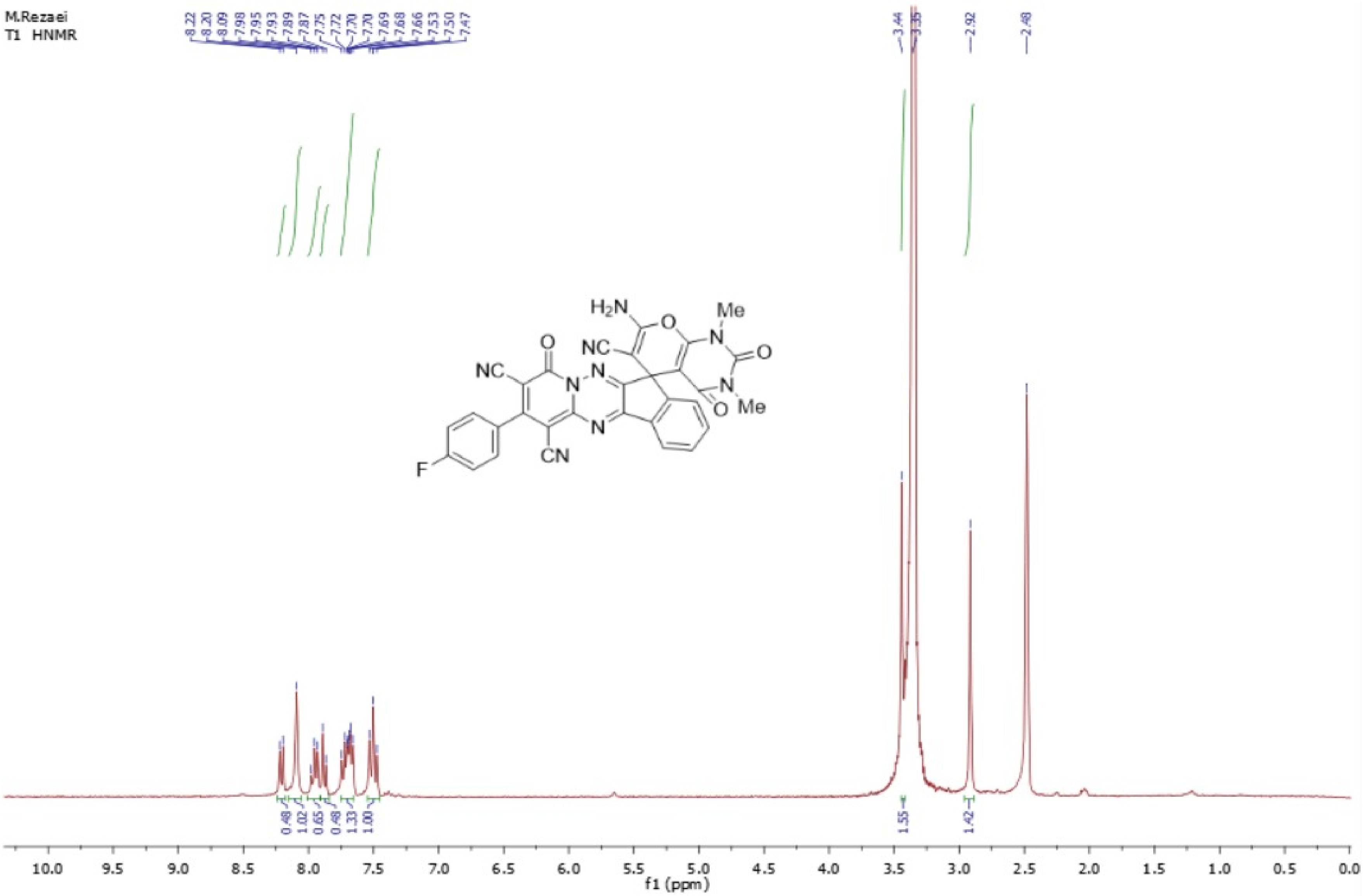
M.Rezaei
T1 HNMR
8.22 8.20 8.09 7.98 7.95 7.93 7.89 7.87 7.75 7.72 7.70 7.70 7.69 7.68 7.66 7.53 7.50 7.47
3.44
3.35
2.92
2.48
H2N
Me
O
NC
N
O
NC
N
N
N
O
N
N
O
Me
F
CN
0.48 1.02 0.65 0.48 1.33 1.00
1.55
1.42
10.0 9.5 9.0 8.5 8.0 7.5 7.0 6.5 6.0 5.5 5.0 4.5 4.0 3.5 3.0 2.5 2.0 1.5 1.0 0.5 0.0
f1 (ppm)

^1H NMR of 6c

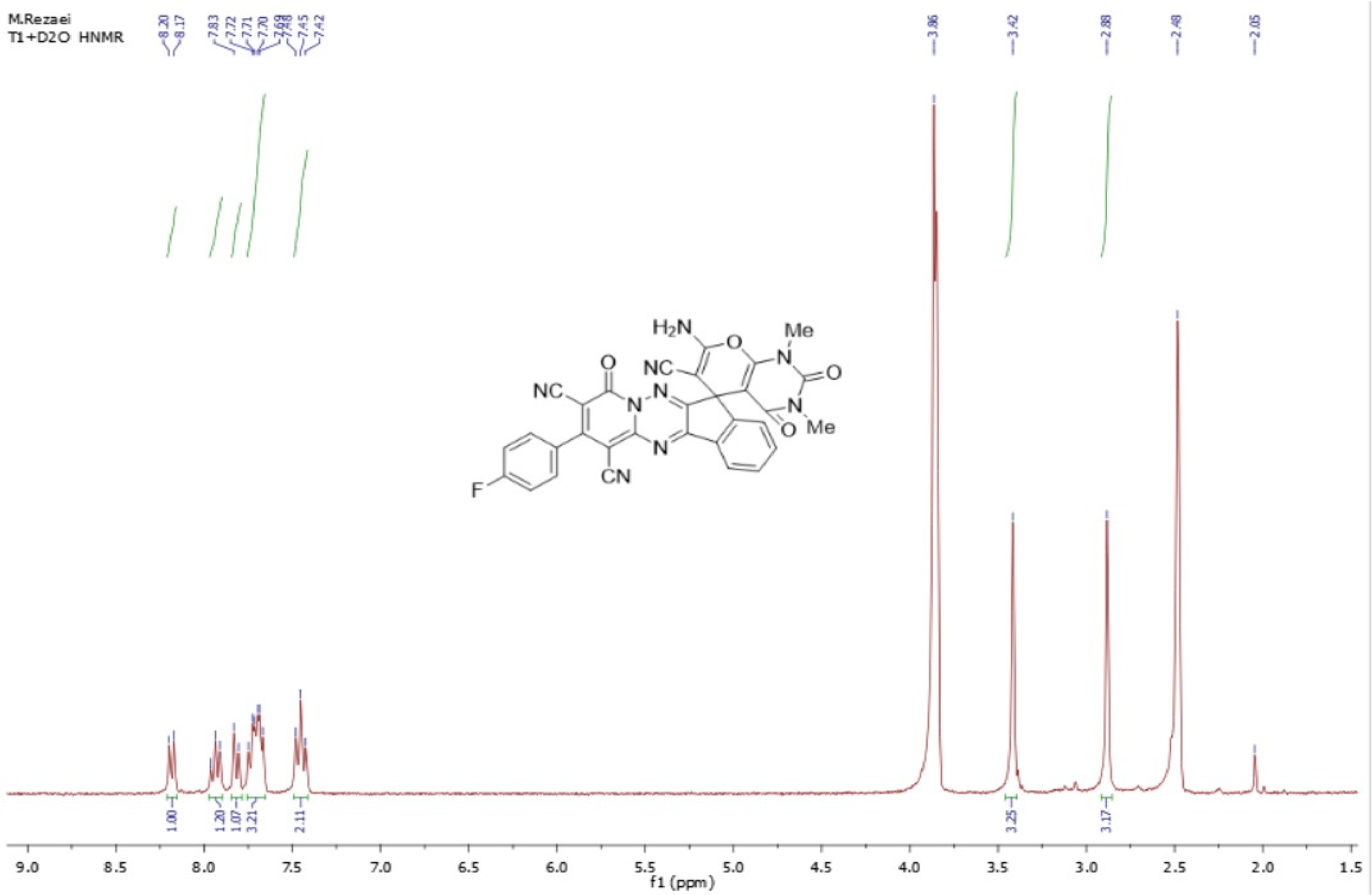

^{1}H NMR of 6c (D$_2$O exchangeable)

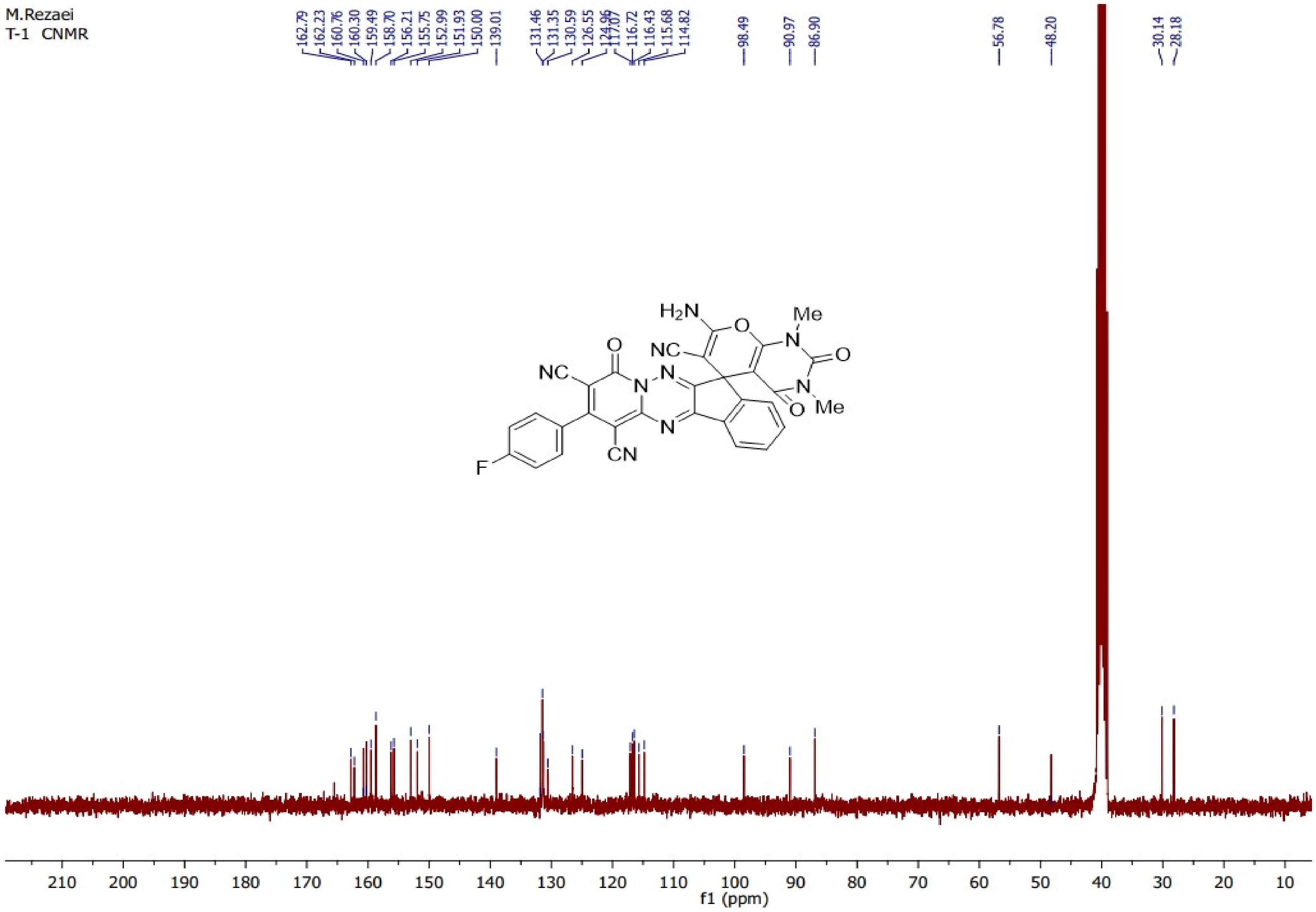
M.Rezaei
T-1 CNMR
162.79
162.23
160.76
160.30
159.49
158.70
156.21
155.75
152.99
151.93
150.00
139.01
131.46
131.35
130.59
126.55
124.96
117.07
116.72
116.43
115.68
114.82
98.49
90.97
86.90
56.78
48.20
30.14
28.18
H2N
O
Me
N
O
NC
O
NC
N
N
N
N
O
Me
N
CN
F
210
200
190
180
170
160
150
140
130
120
110
100
90
80
70
60
50
40
30
20
10
f1 (ppm)

^{13}C NMR of 6c

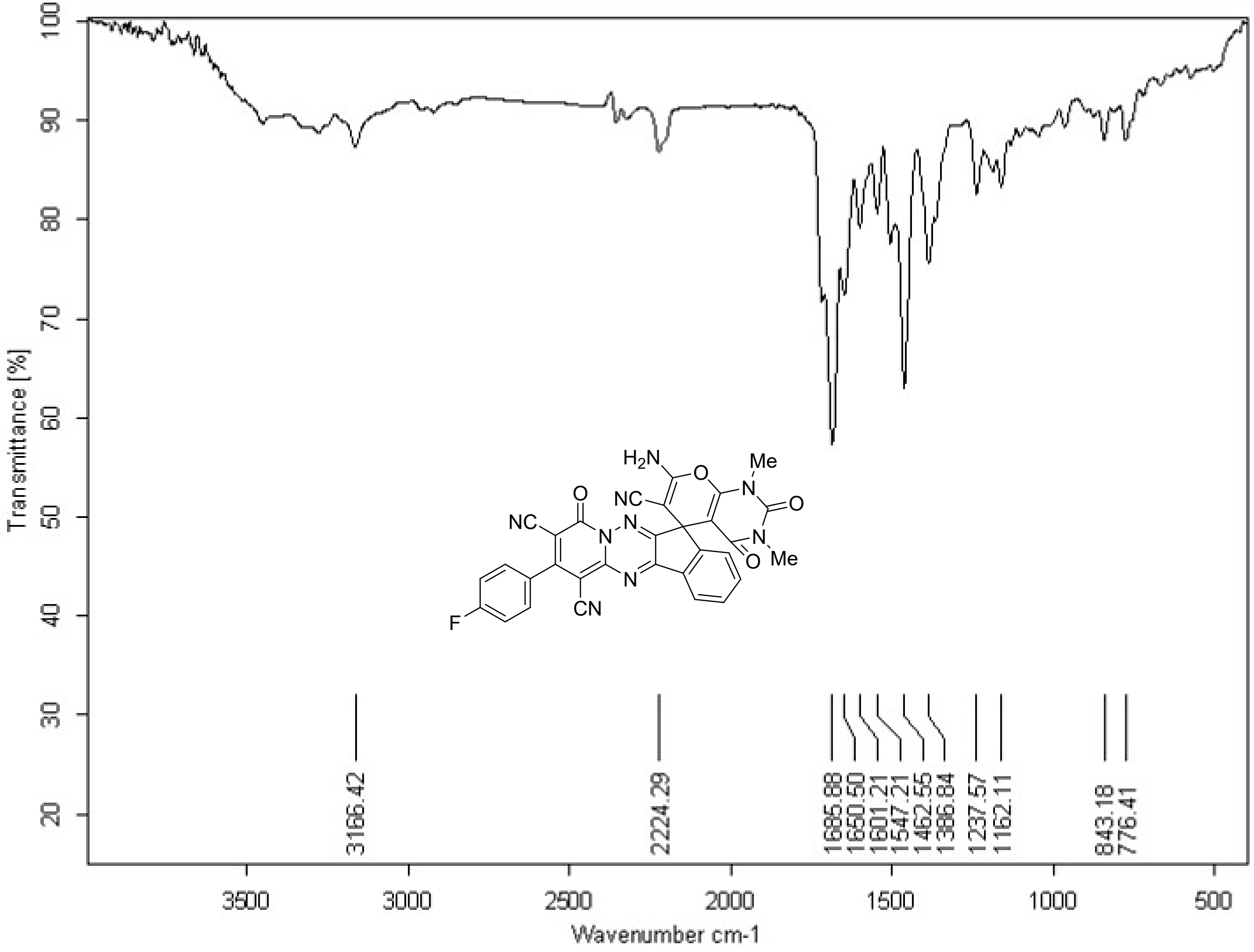

100
90
80
70
60
50
40
30
20
Transmittance [%]
3166.42
2224.29
1685.88
1650.50
1601.21
1547.21
1462.55
1386.84
1237.57
1162.11
843.18
776.41
3500
3000
2500
2000
1500
1000
500
Wavenumber cm-1

IR of 6c

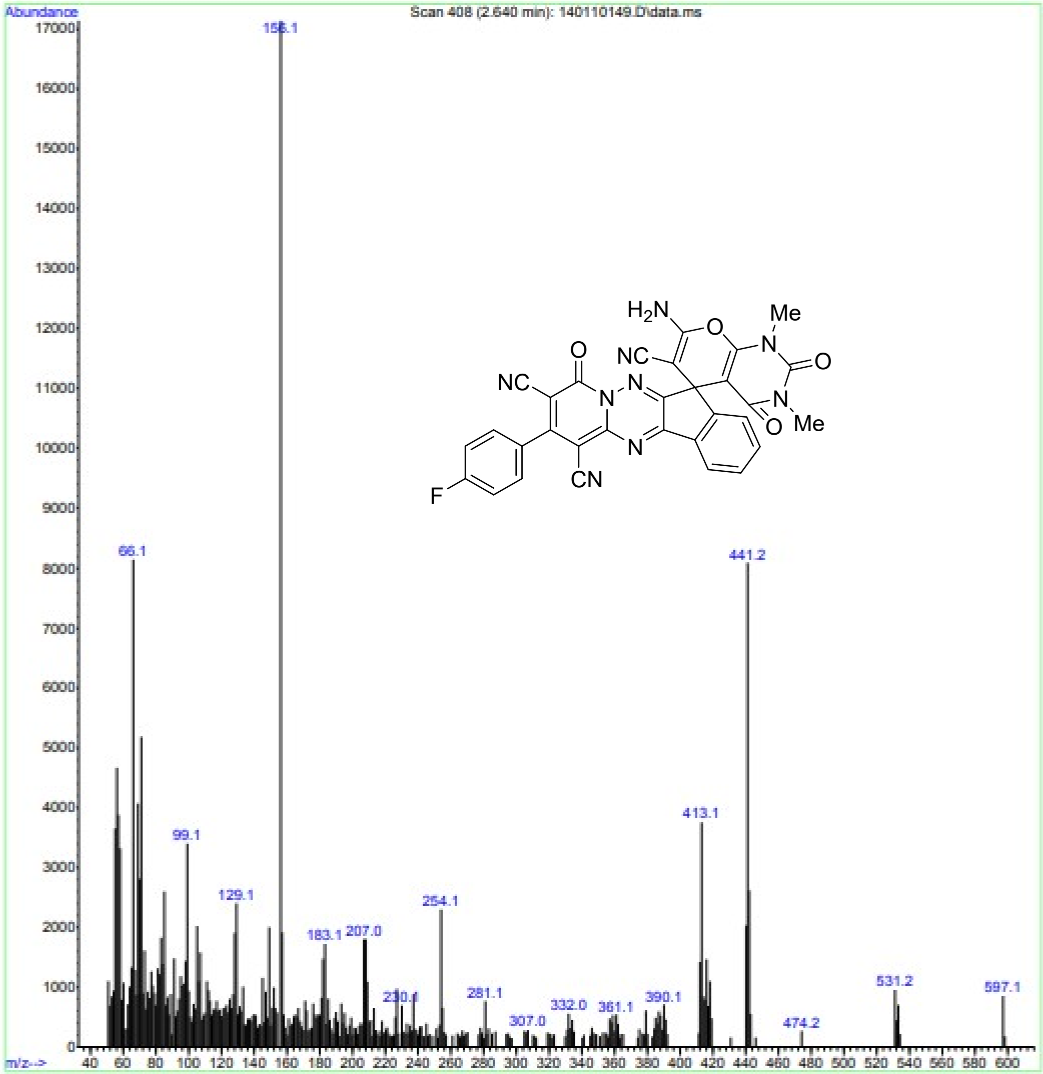
Abundance
Scan 408 (2.640 min): 140110149.D\data.ms
156.1
66.1
99.1
129.1
183.1
207.0
254.1
281.1
307.0
332.0
361.1
390.1
413.1
441.2
474.2
531.2
597.1
m/z-->
H2N
O
Me
N
NC
O
NC
O
N
N
N
N
N
O
Me
N
CN
F

MS of 6c

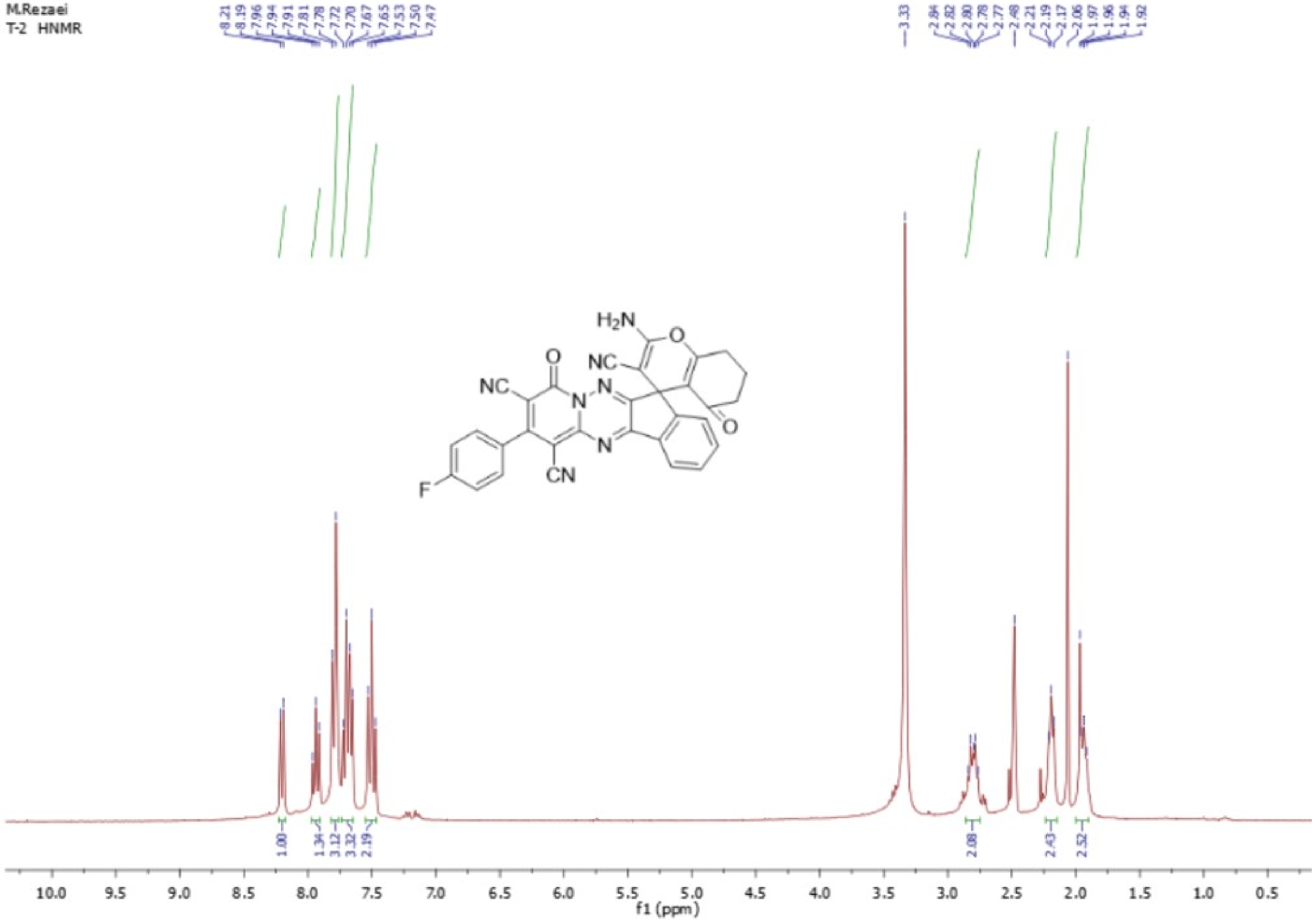
M.Rezaei
T-2 HNMR
8.21
8.19
7.96
7.94
7.91
7.81
7.78
7.72
7.70
7.67
7.65
7.53
7.50
7.47
3.33
2.84
2.82
2.80
2.78
2.77
2.48
2.21
2.19
2.17
2.06
1.97
1.96
1.94
1.92
H2N
NC
O
NC
N
N
N
N
CN
F
O
O
1.00
1.34
3.12
3.32
2.19
2.08
2.43
2.52
10.0
9.5
9.0
8.5
8.0
7.5
7.0
6.5
6.0
5.5
5.0
4.5
4.0
3.5
3.0
2.5
2.0
1.5
1.0
0.5
f1 (ppm)

^{1}H NMR of 6d

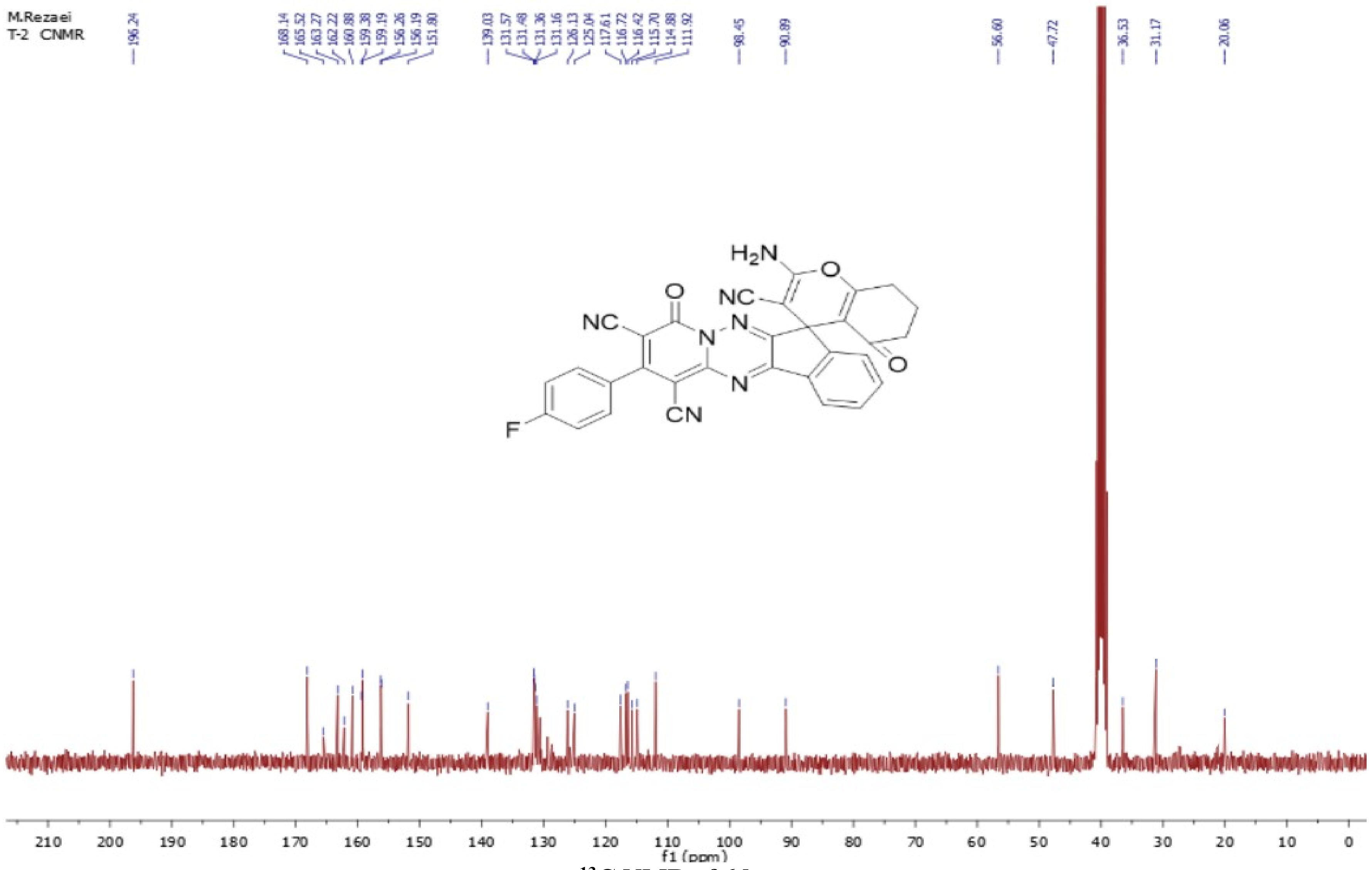
M.Rezaei
T-2 CNMR
196.24
168.14
165.52
163.27
162.22
160.88
159.38
159.19
156.26
156.19
151.80
139.03
131.57
131.48
131.36
131.16
126.13
125.04
117.61
116.72
116.42
115.70
114.88
111.92
98.45
90.89
56.60
47.72
36.53
31.17
20.06
H2N
O
NC
O
NC
N
N
N
O
CN
F
210 200 190 180 170 160 150 140 130 120 110 100 90 80 70 60 50 40 30 20 10 0
f1 (ppm)

^{13}C NMR of 6d

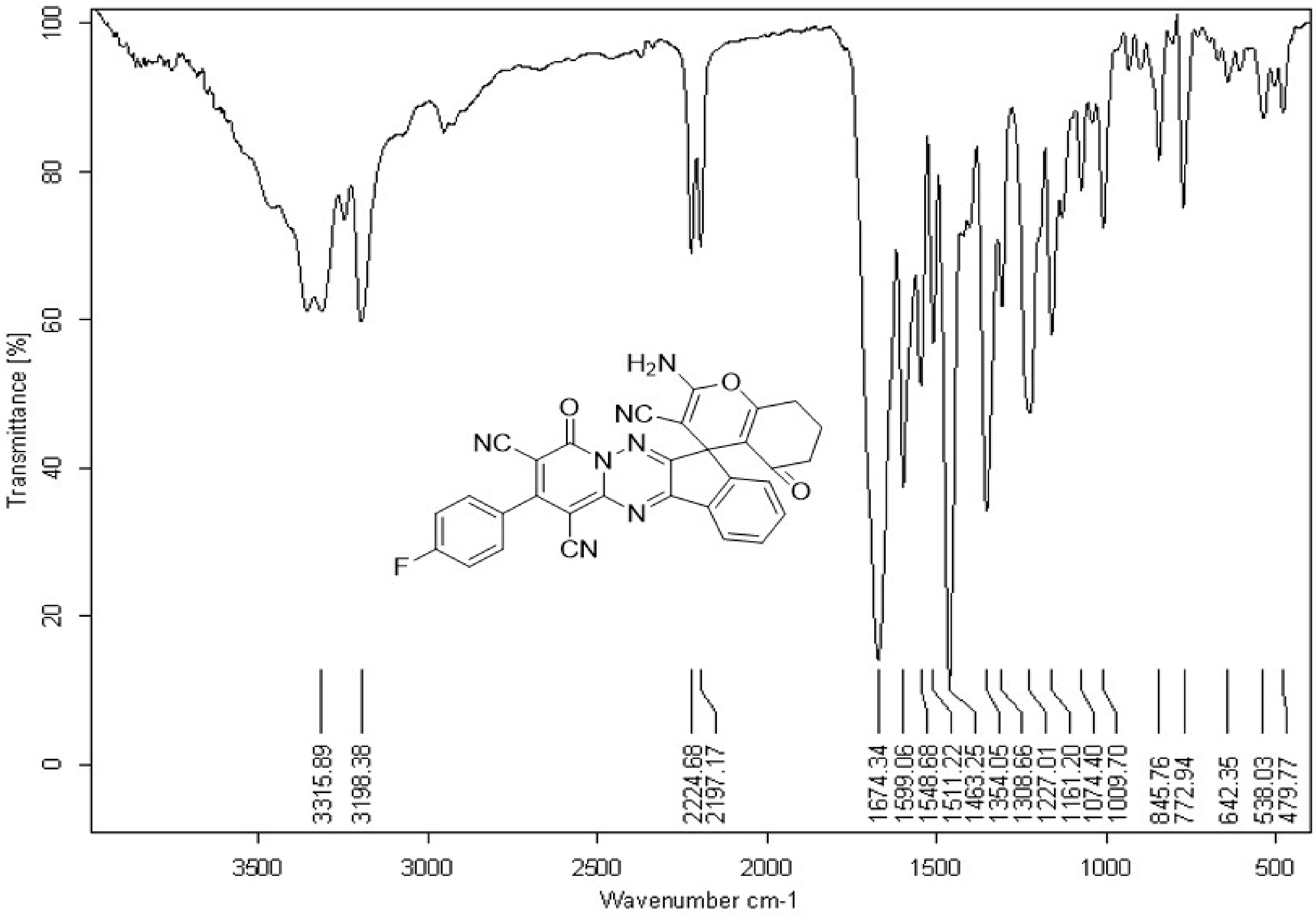

Transmittance [%]
Wavenumber cm-1
100
80
60
40
20
0
3500
3000
2500
2000
1500
1000
500
3315.89
3198.38
2224.68
2197.17
1674.34
1599.06
1548.68
1511.22
1463.25
1354.05
1308.66
1227.01
1161.20
1074.40
1009.70
845.76
772.94
642.35
538.03
479.77
H2N
O
NC
O
NC
N
N
N
O
F
CN

IR of 6d

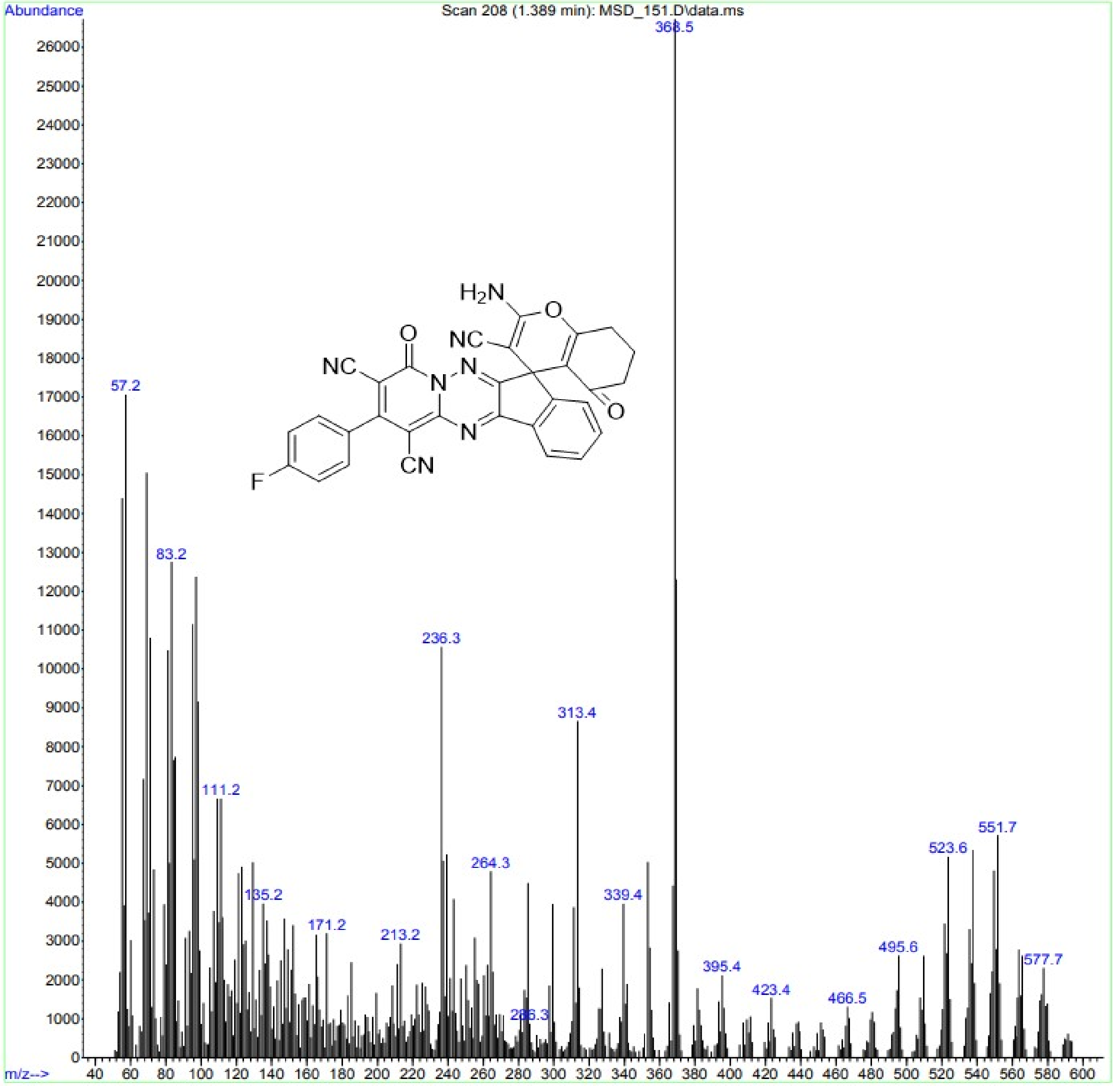
Abundance
Scan 208 (1.389 min): MSD_151.D\data.ms
368.5
57.2
83.2
111.2
135.2
171.2
213.2
236.3
264.3
286.3
313.4
339.4
395.4
423.4
466.5
495.6
523.6
551.7
577.7
m/z-->
H2N
O
NC
NC
N
CN
F

MS of 6d

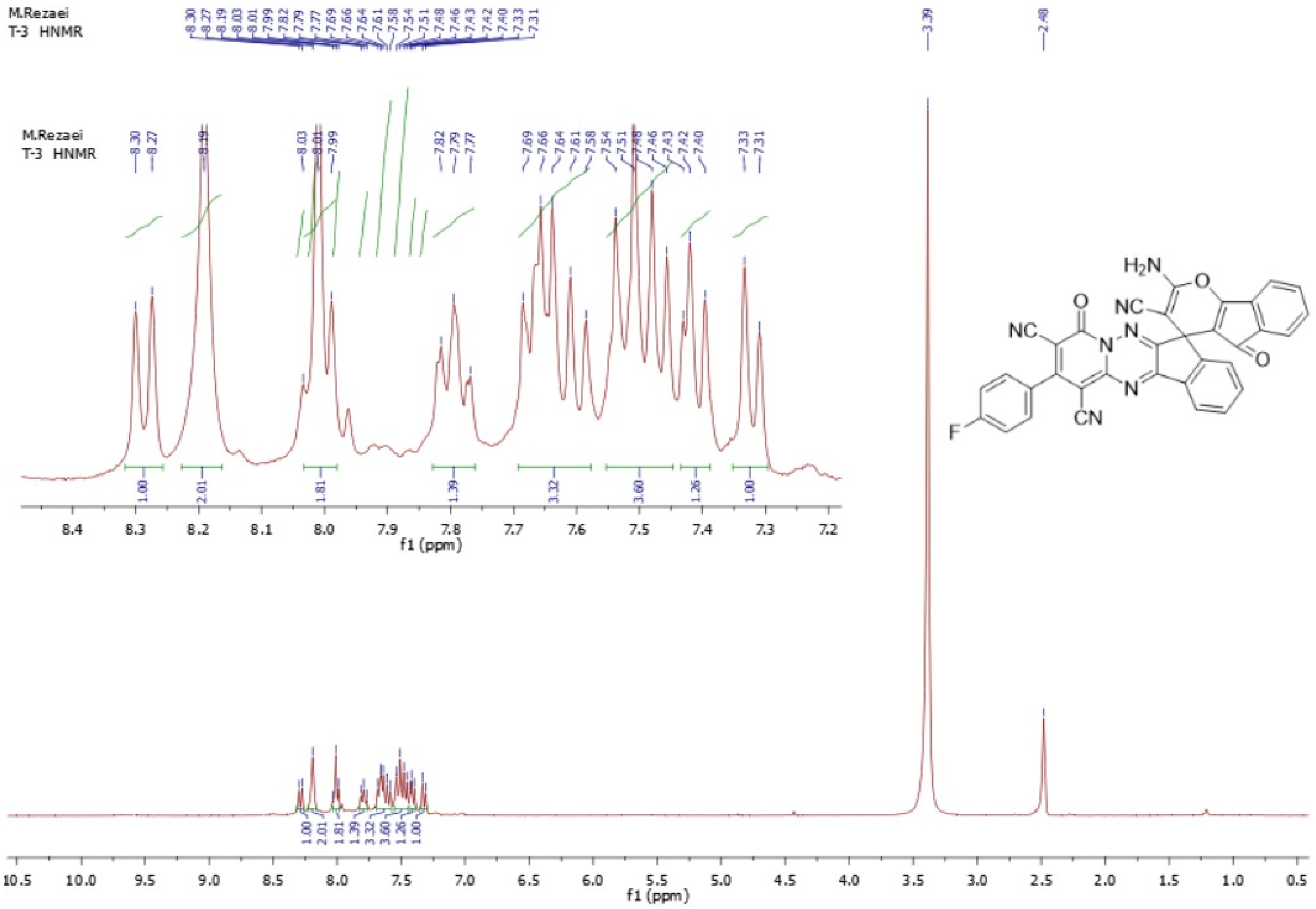
M.Rezaei
T-3 HNMR
8.30 8.27 8.19 8.03 8.01 7.99 7.82 7.79 7.77 7.69 7.66 7.64 7.61 7.58 7.54 7.51 7.48 7.46 7.43 7.42 7.40 7.33 7.31
3.39
2.48
1.00 2.01 1.81 1.39 3.32 3.60 1.26 1.00
f1 (ppm)
H2N
O
NC
N
F
CN

^{1}H NMR of 6e

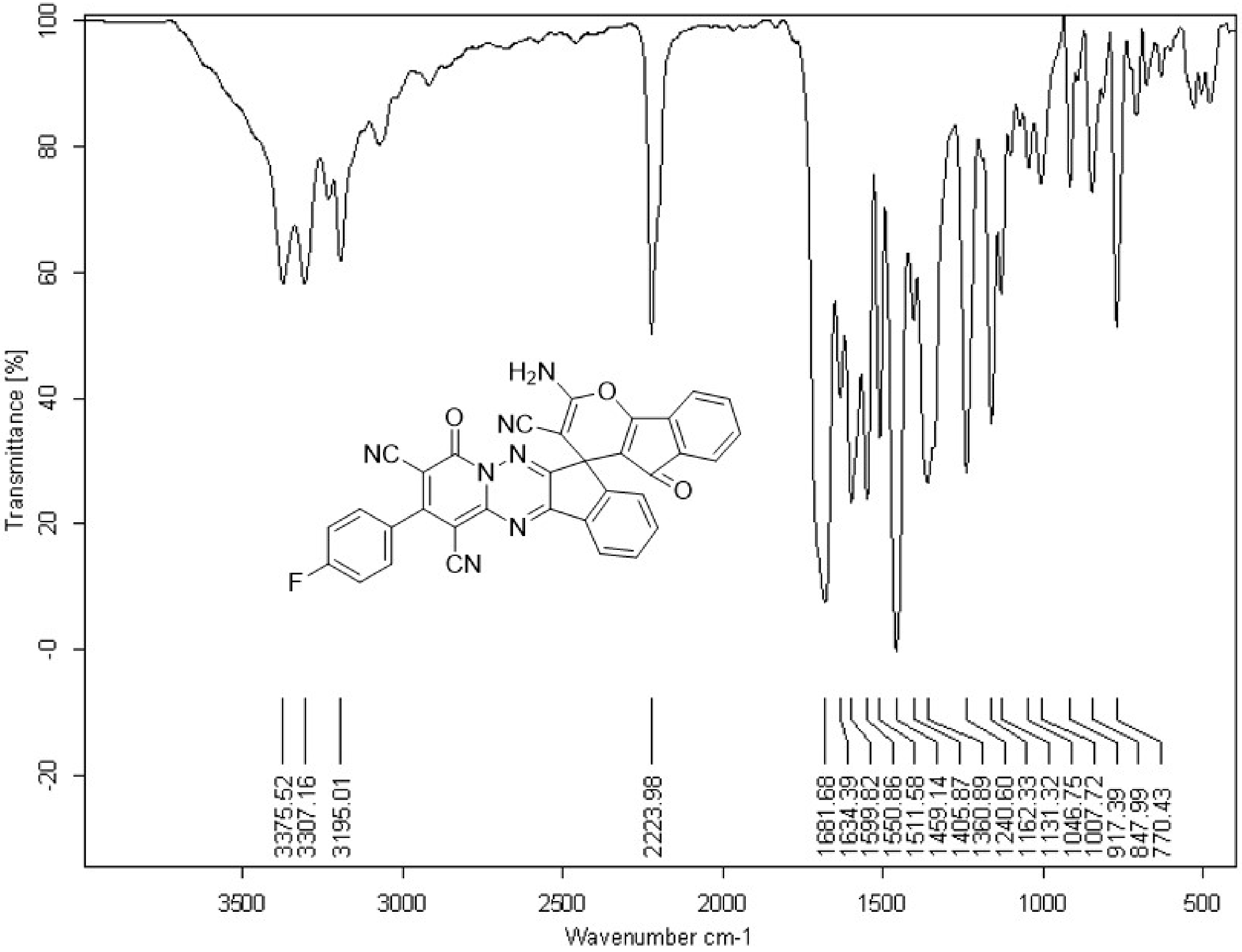
Transmittance [%]
Wavenumber cm-1
100
80
60
40
20
-0
-20
3500
3000
2500
2000
1500
1000
500
3375.52
3307.16
3195.01
2223.98
1681.68
1634.39
1599.82
1550.86
1511.58
1459.14
1405.87
1360.89
1240.60
1162.33
1131.32
1046.75
1007.72
917.39
847.99
770.43
H2N
O
NC
O
NC
N
N
O
N
N
F
CN

IR of 6e

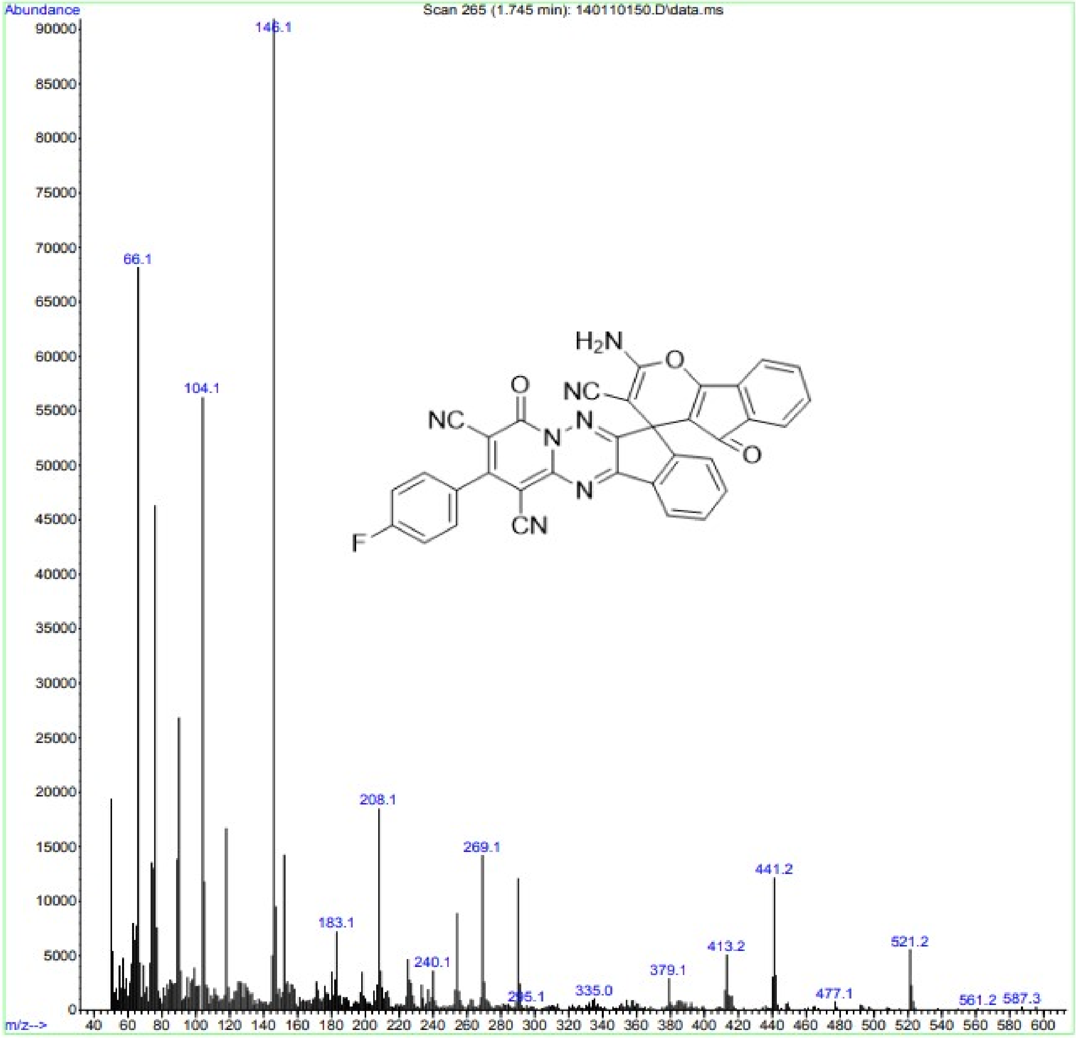
Abundance
Scan 265 (1.745 min): 140110150.D\data.ms
90000
85000
80000
75000
70000
65000
60000
55000
50000
45000
40000
35000
30000
25000
20000
15000
10000
5000
0
m/z-->
40 60 80 100 120 140 160 180 200 220 240 260 280 300 320 340 360 380 400 420 440 460 480 500 520 540 560 580 600
146.1
66.1
104.1
208.1
269.1
183.1
240.1
295.1
335.0
379.1
413.2
441.2
477.1
521.2
561.2
587.3
H2N
O
NC
NC
O
N
N
N
N
O
F
CN

MS of 6e

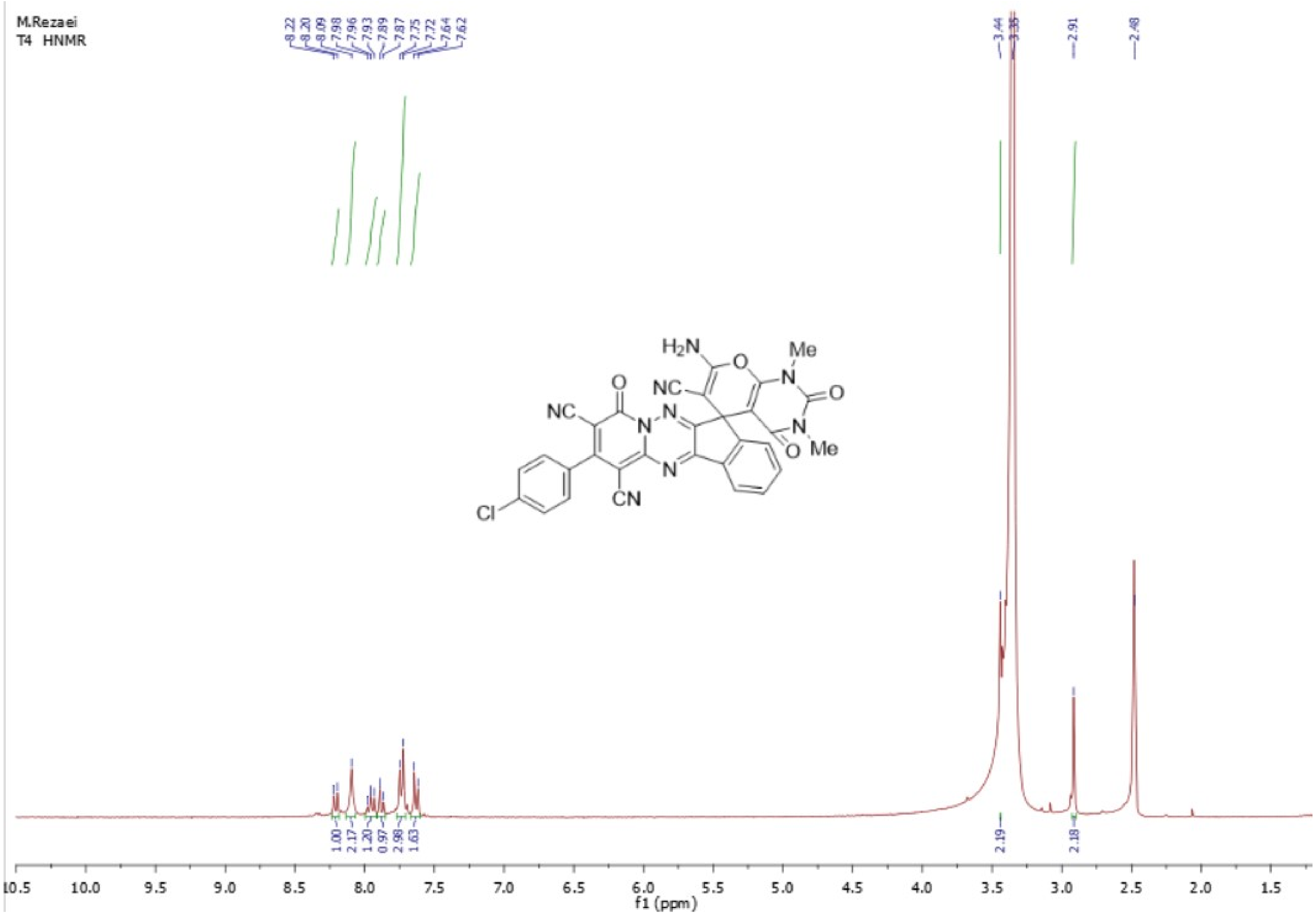
M.Rezaei
T4 HNMR
8.22
8.20
8.09
7.98
7.96
7.93
7.89
7.87
7.75
7.72
7.64
7.62
3.44
3.38
2.91
2.48
H2N
O
Me
NC
N
Cl
CN
1.00
2.17
1.20
0.97
2.98
1.63
2.19
2.18
f1 (ppm)

^{1}H NMR of 6f

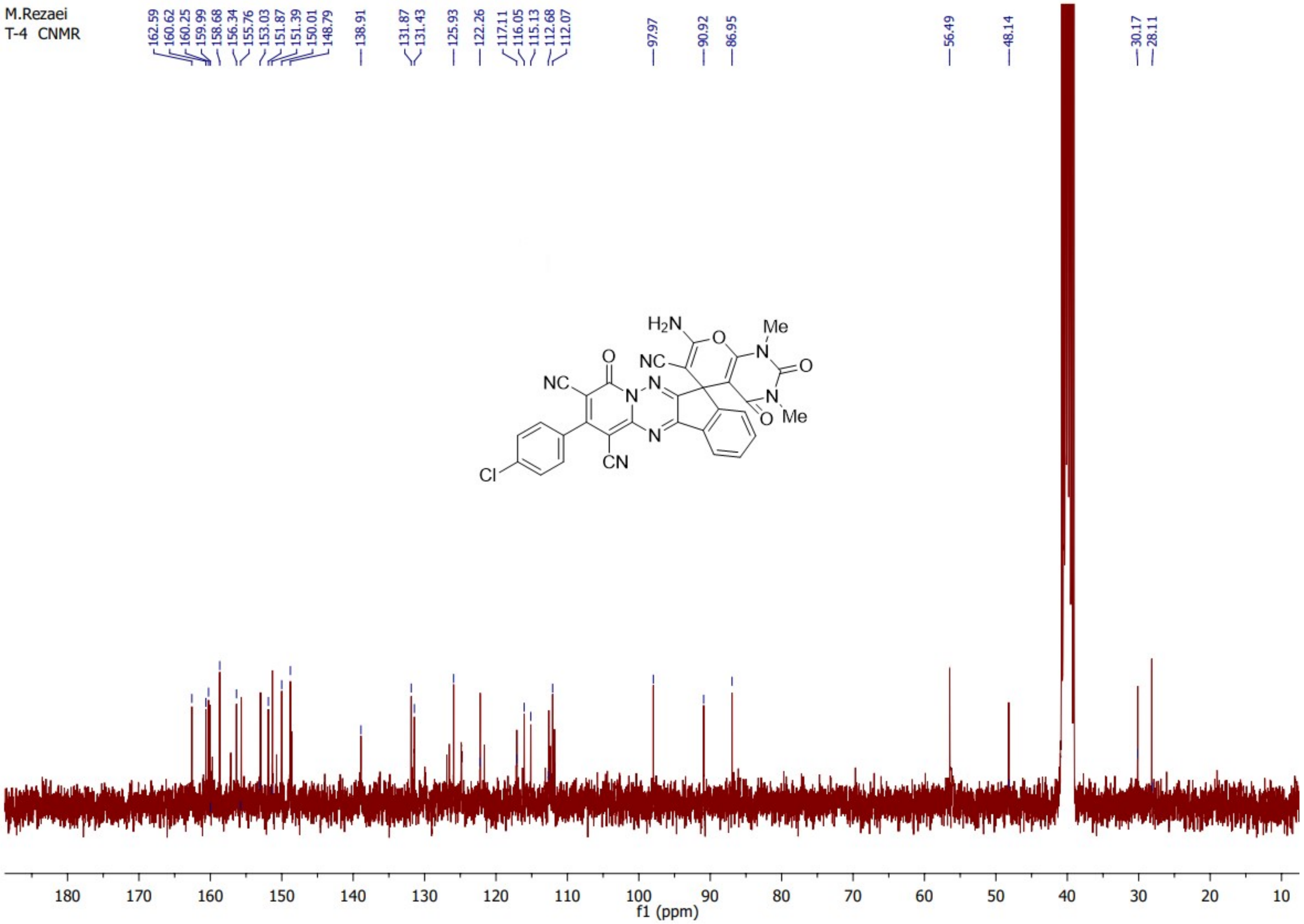
M.Rezaei
T-4 CNMR
162.59
160.62
160.25
159.99
158.68
156.34
155.76
153.03
151.87
151.39
150.01
148.79
138.91
131.87
131.43
125.93
122.26
117.11
116.05
115.13
112.68
112.07
97.97
90.92
86.95
56.49
48.14
30.17
28.11
f1 (ppm)

^{13}C NMR of 6f

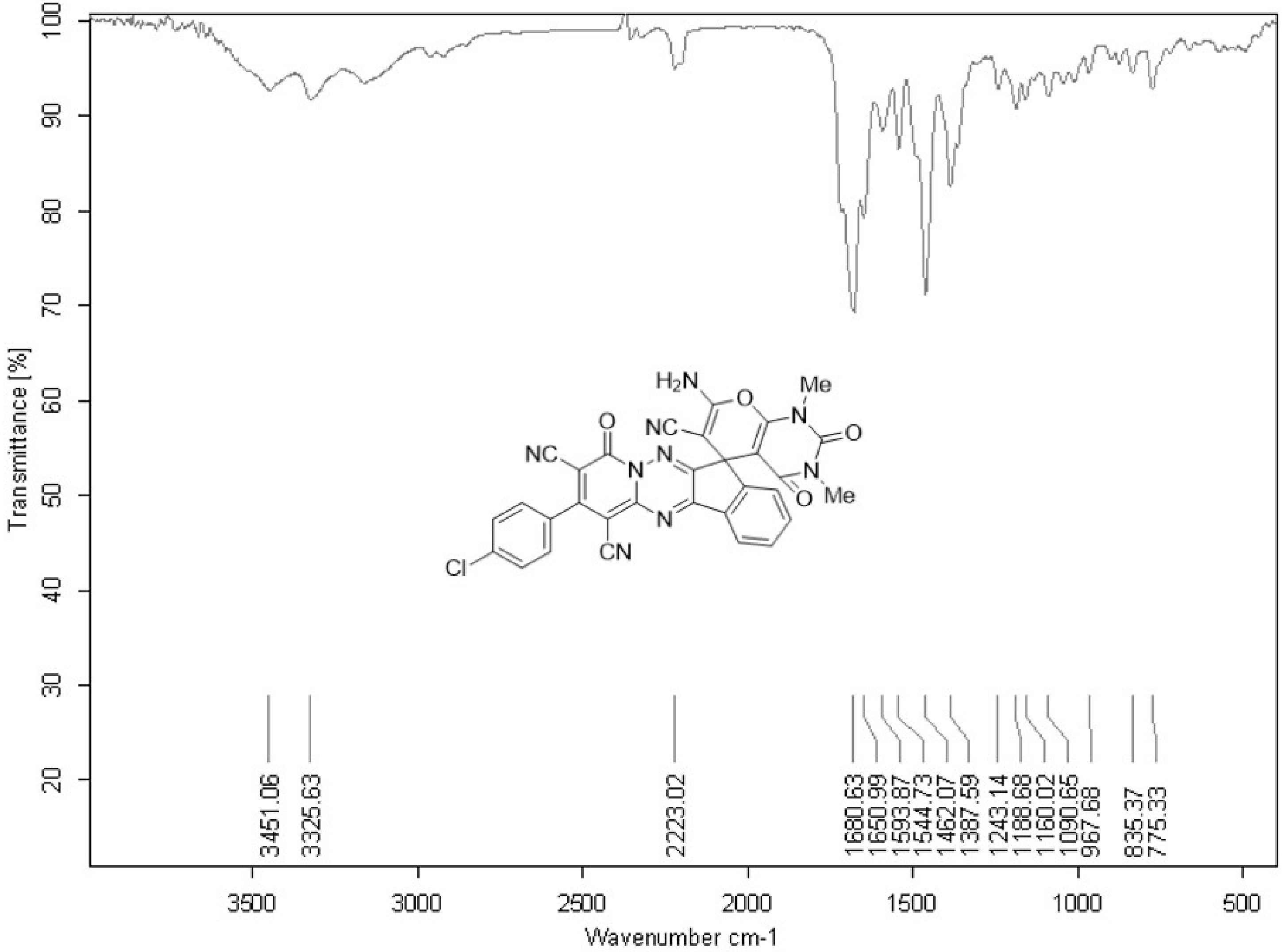
100
90
80
70
60
50
40
30
20
Transmittance [%]
H2N
O
Me
N
NC
O
O
NC
N
N
N
O
N
Me
O
N
Cl
CN
3451.06
3325.63
2223.02
1680.63
1650.99
1593.87
1544.73
1462.07
1387.59
1243.14
1188.68
1160.02
1090.65
967.68
835.37
775.33
3500
3000
2500
2000
1500
1000
500
Wavenumber cm-1

IR of 6f

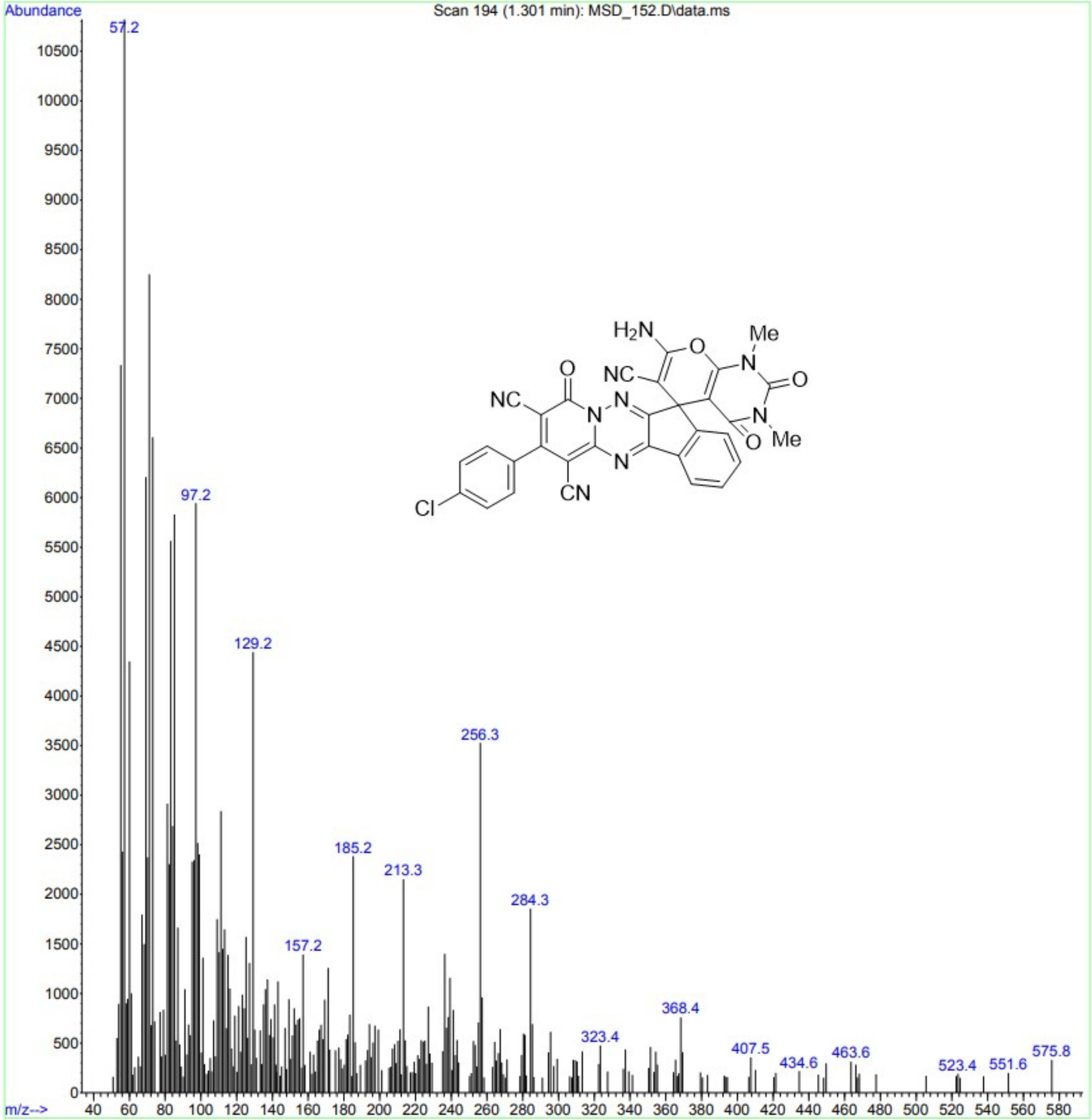
Abundance
Scan 194 (1.301 min): MSD_152.D\data.ms
57.2
97.2
129.2
157.2
185.2
213.3
256.3
284.3
323.4
368.4
407.5
434.6
463.6
523.4
551.6
575.8
m/z-->
H2N
NC
O
Me
N
NC
N
O
O
Cl
CN

MS of 6f

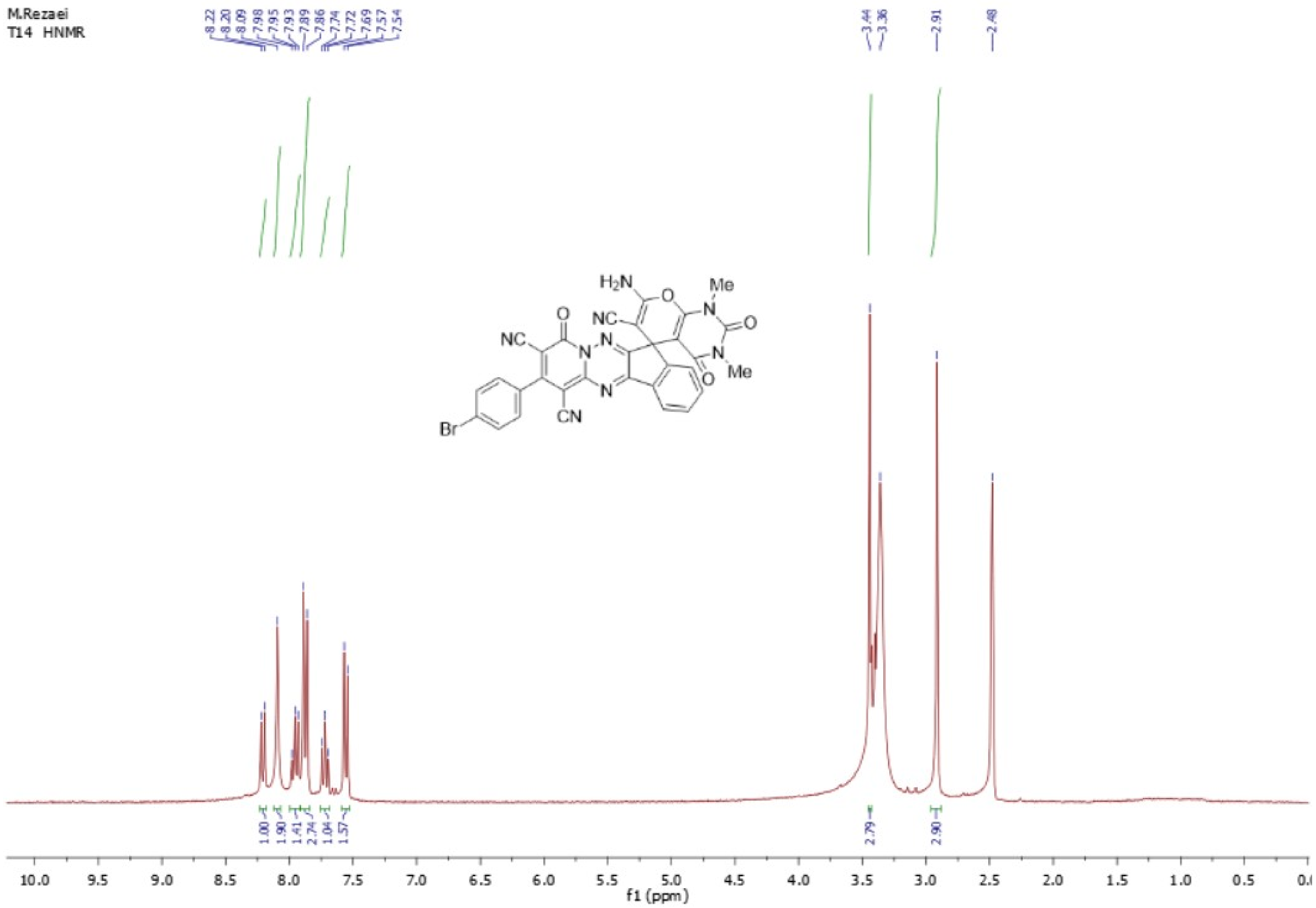

M.Rezaei
T14 HNMR
8.22
8.20
8.09
7.98
7.95
7.93
7.89
7.86
7.74
7.72
7.69
7.57
7.54
3.44
3.36
2.91
2.48
1.00
1.90
1.41
2.74
1.04
1.57
2.79
2.90
f1 (ppm)

^{1}H NMR of 6g

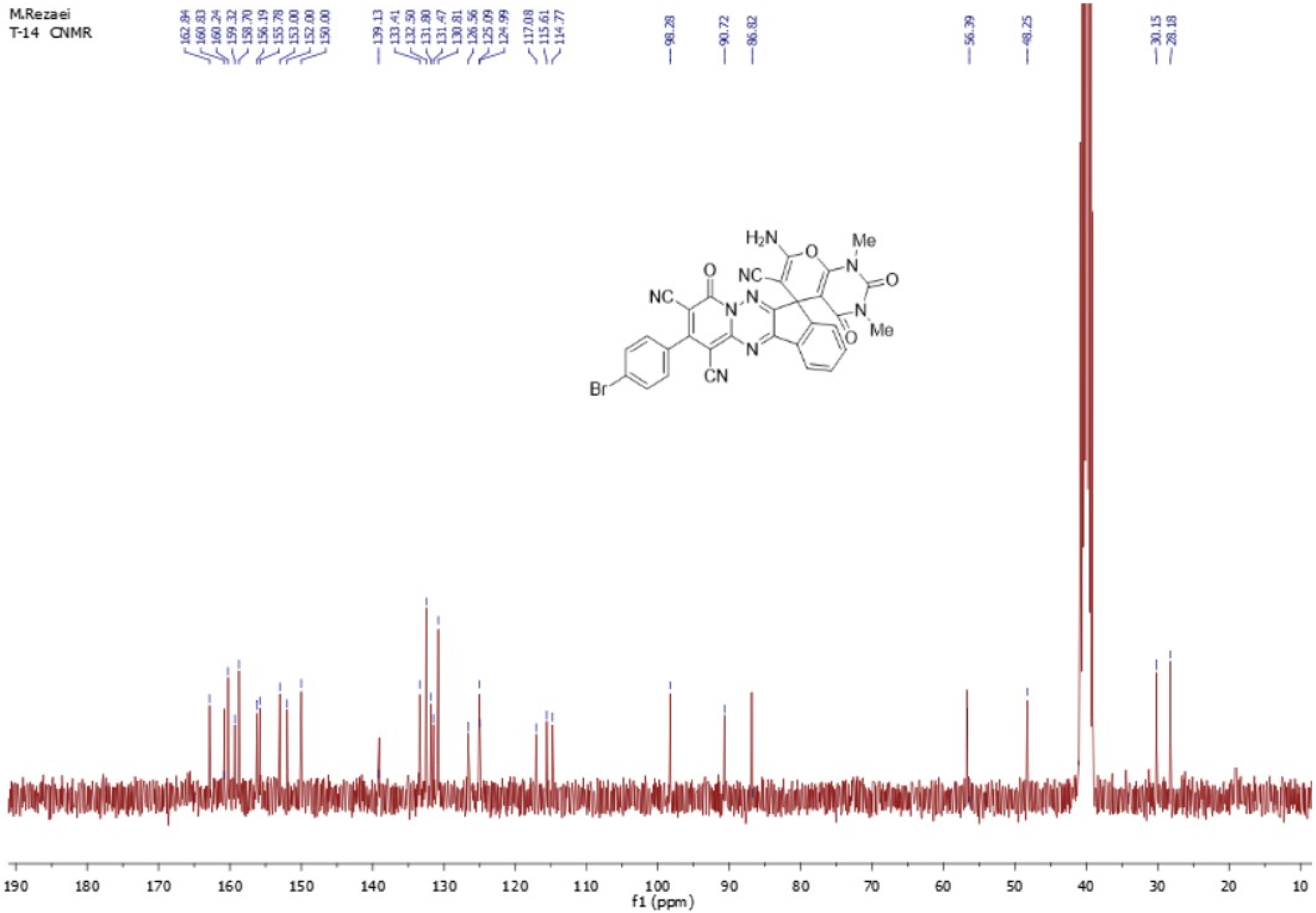
M.Rezaei
T-14 CNMR
162.84
160.83
160.24
159.32
158.70
156.19
155.78
153.00
152.00
150.00
139.13
133.41
132.50
131.80
131.47
130.81
126.56
125.09
124.99
117.08
115.61
114.77
98.28
90.72
86.82
56.39
48.25
30.15
28.18
H_2N
O
Me
N
O
NC
NC
N
N
O
N
Me
N
N
O
Br
CN
190
180
170
160
150
140
130
120
110
100
90
80
70
60
50
40
30
20
10
f1 (ppm)

^{13}C NMR of 6g

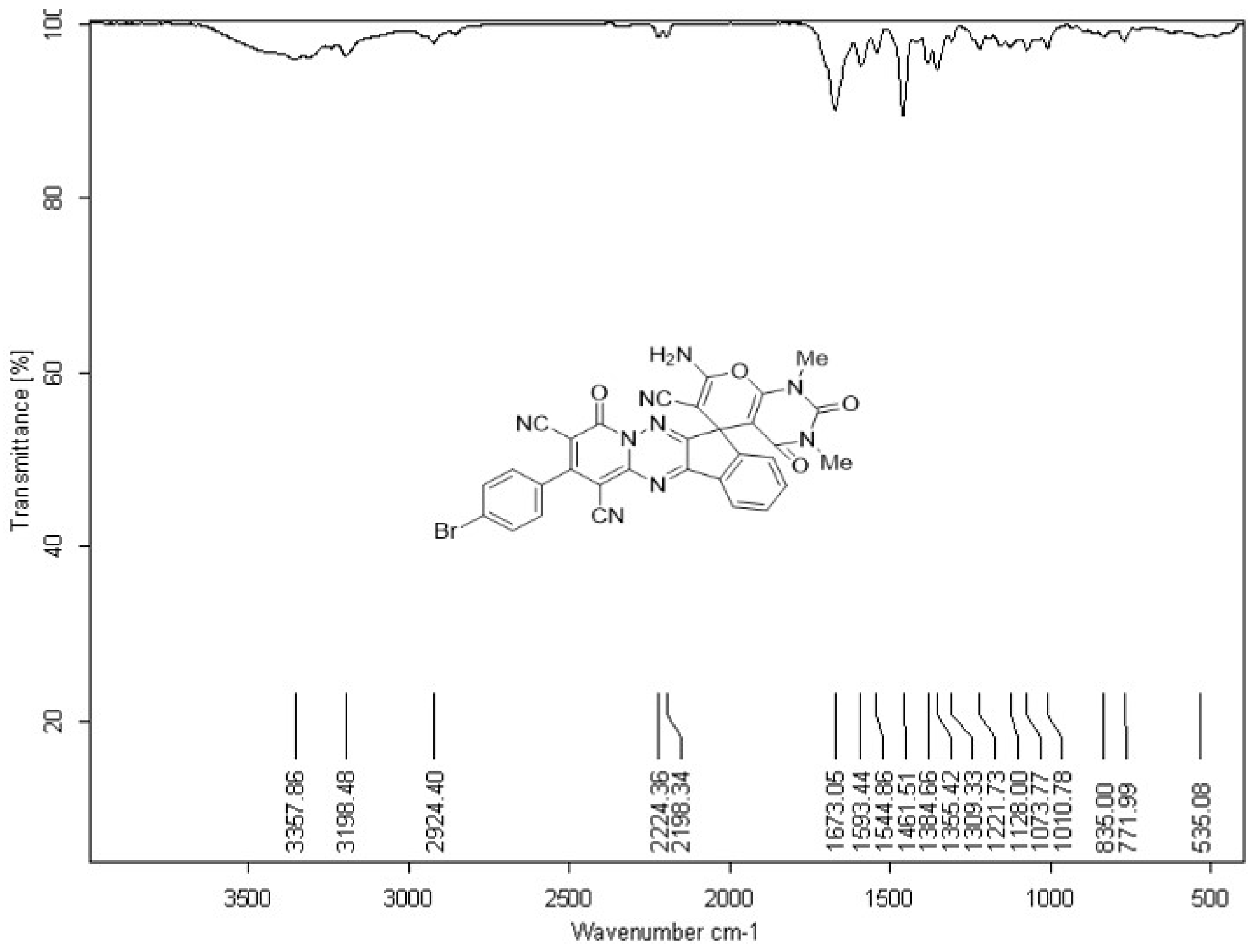
Transmittance [%]
Wavenumber cm-1
3357.86
3198.48
2924.40
2224.36
2198.34
1673.05
1593.44
1544.86
1461.51
1384.66
1355.42
1309.33
1221.73
1128.00
1073.77
1010.78
835.00
771.99
535.08

IR of 6g

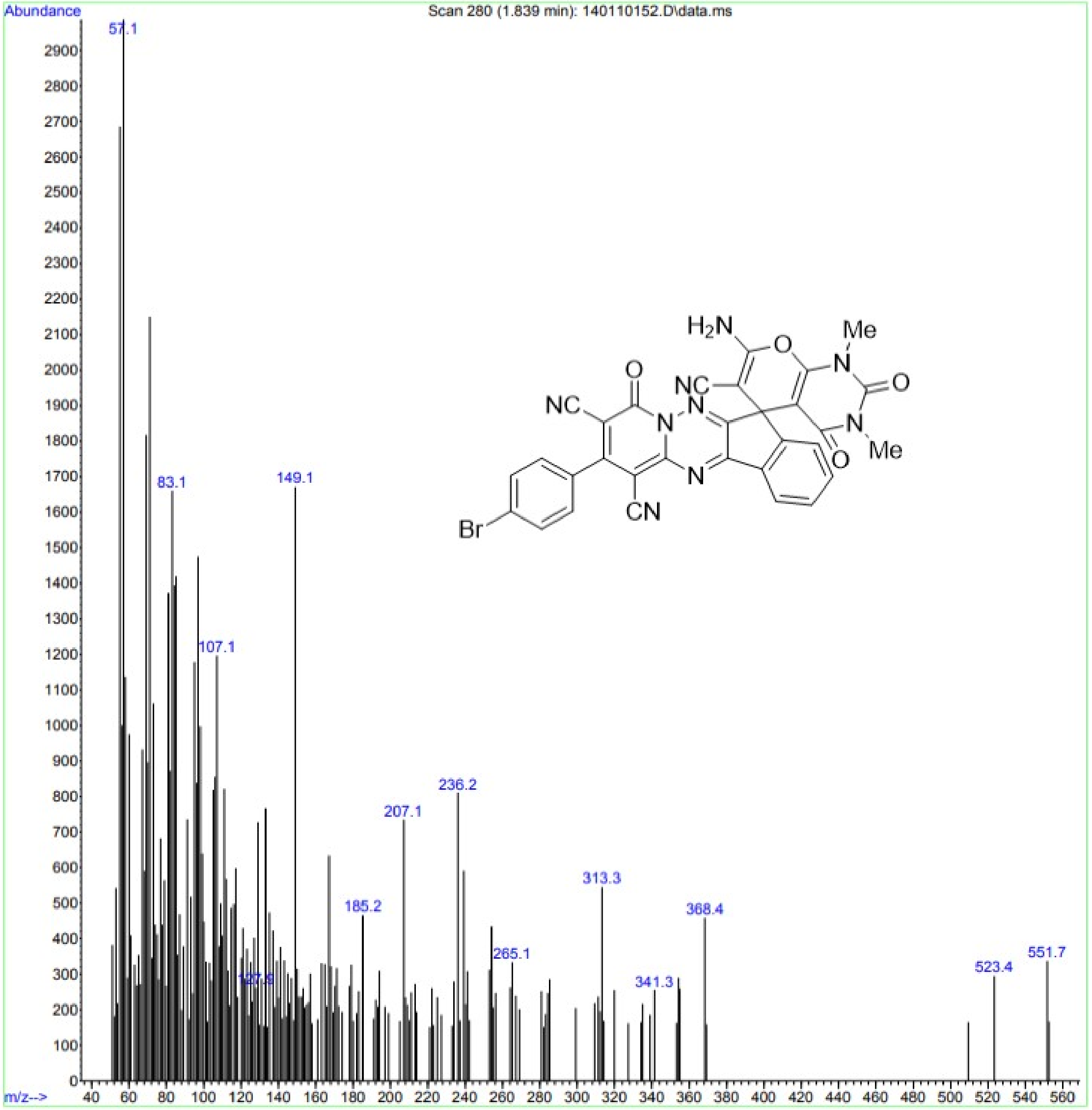
Abundance
Scan 280 (1.839 min): 140110152.D\data.ms
57.1
83.1
149.1
107.1
207.1
236.2
185.2
313.3
368.4
265.1
341.3
523.4
551.7
m/z-->
H2N
Me
O
NC
N
NC
CN
Br

MS of 6g

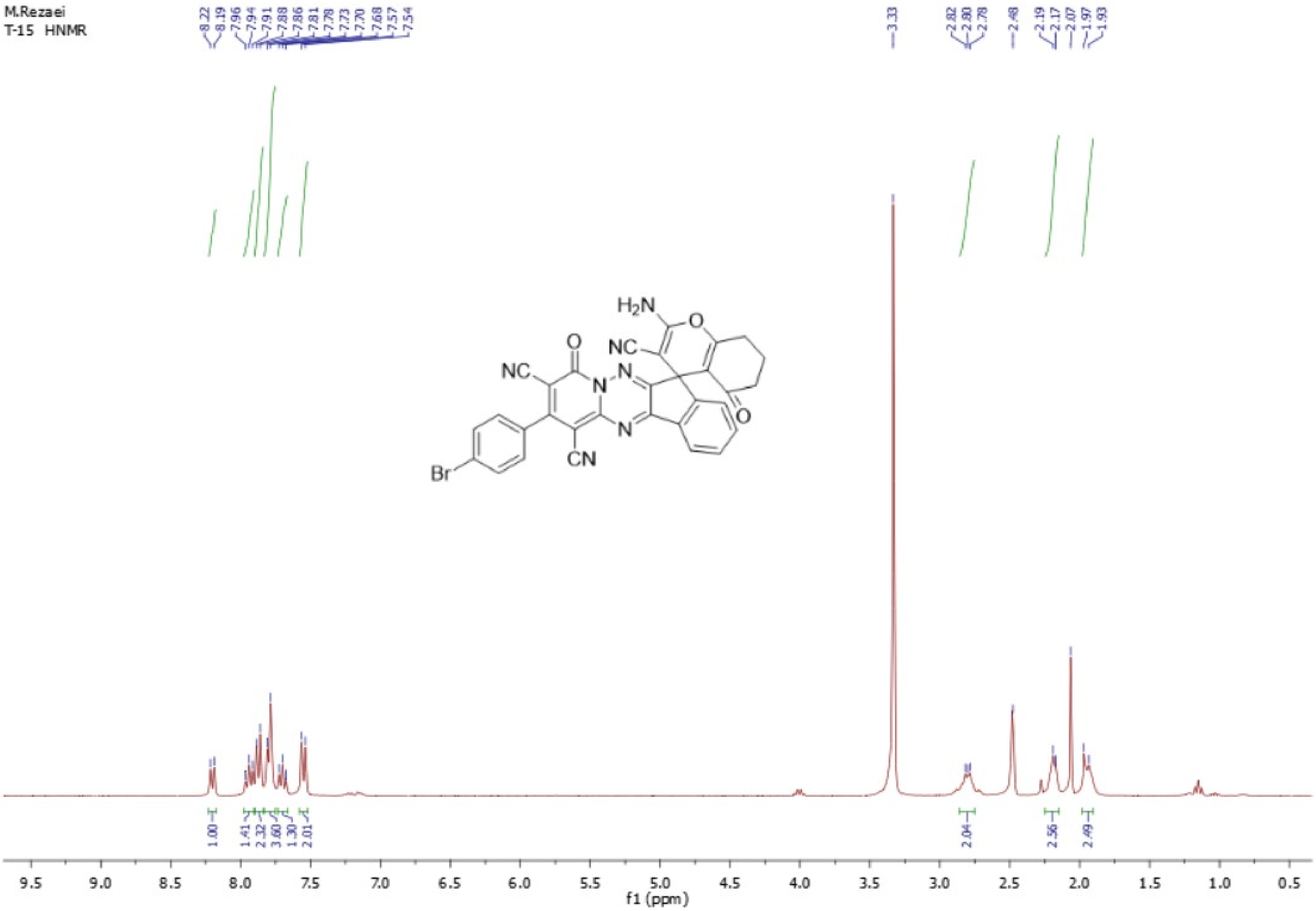
M.Rezaei
T-15 HNMR
8.22
8.19
7.96
7.94
7.91
7.88
7.86
7.81
7.78
7.73
7.70
7.68
7.57
7.54
3.33
2.82
2.80
2.78
2.48
2.19
2.17
2.07
1.97
1.93
H_2N
O
NC
O
NC
N
N
N
N
CN
Br
O
1.00
1.41
2.32
3.60
1.30
2.01
2.04
2.56
2.49
9.5
9.0
8.5
8.0
7.5
7.0
6.5
6.0
5.5
5.0
4.5
4.0
3.5
3.0
2.5
2.0
1.5
1.0
0.5
f1 (ppm)

^{1}H NMR of 6h

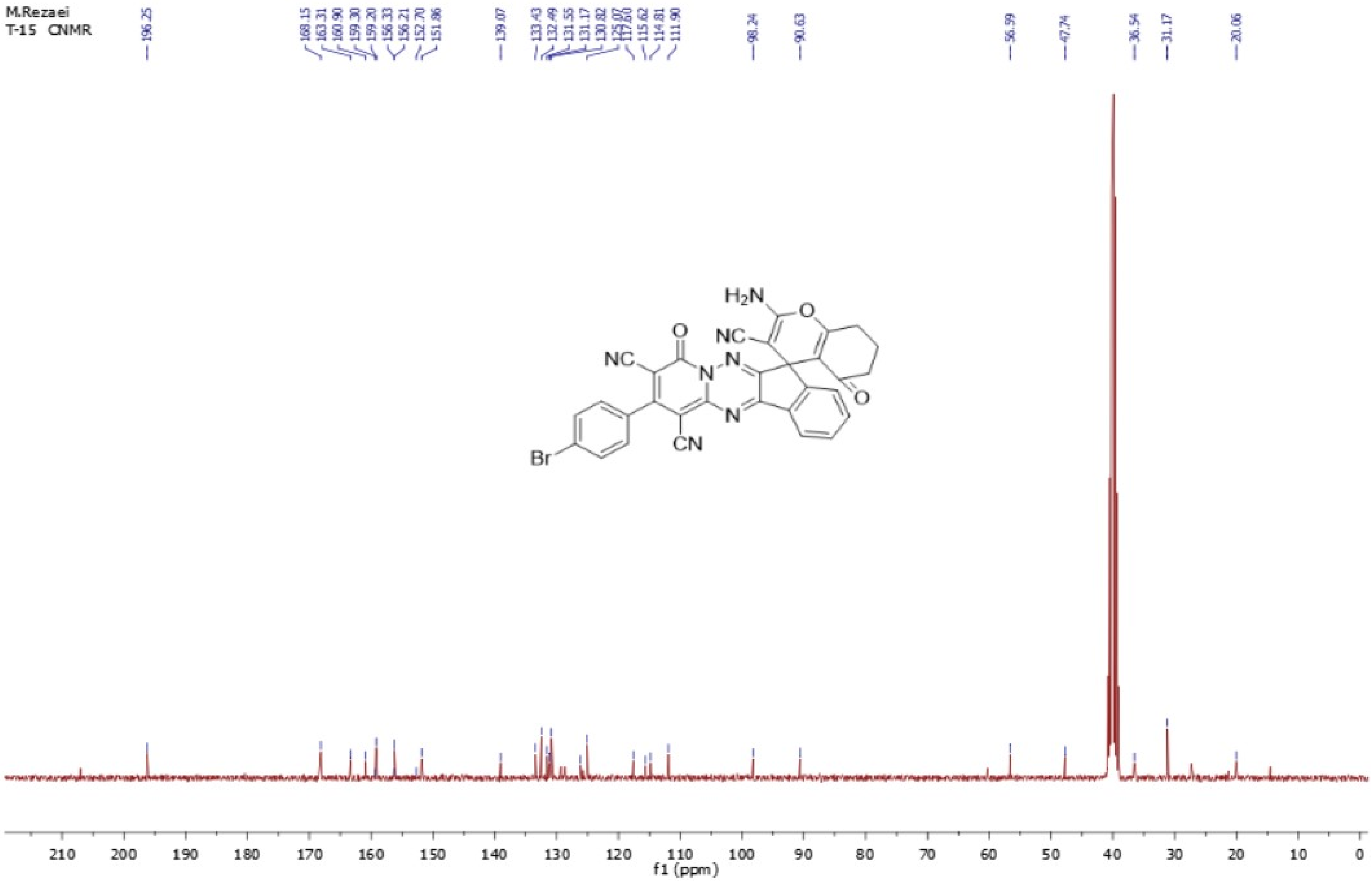
M.Rezaei
T-15 CNMR
196.25
168.15
163.31
160.90
159.30
159.20
156.33
156.21
152.70
151.86
139.07
133.43
132.49
131.55
131.17
130.82
115.62
114.81
111.90
98.24
90.63
56.99
47.74
36.54
31.17
20.06
H2N
O
NC
NC
N
N
N
CN
Br
210 200 190 180 170 160 150 140 130 120 110 100 90 80 70 60 50 40 30 20 10 0
f1 (ppm)

^{13}C NMR of 6h

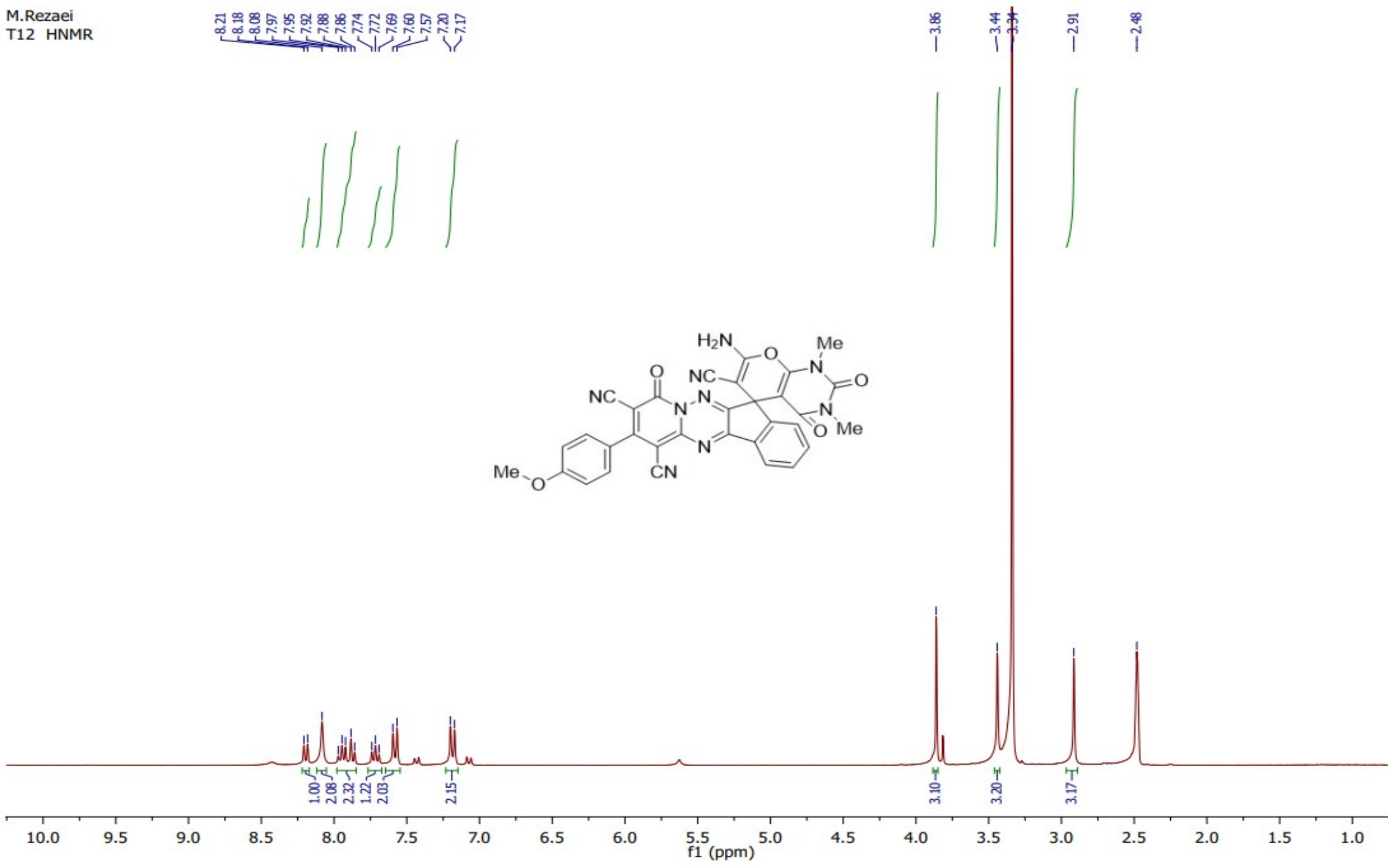
M.Rezaei
T12 HNMR
8.21
8.18
8.08
7.97
7.95
7.92
7.88
7.86
7.74
7.72
7.69
7.60
7.57
7.20
7.17
3.86
3.44
3.34
2.91
2.48
1.00
2.08
2.32
1.22
2.03
2.15
3.10
3.20
3.17
10.0
9.5
9.0
8.5
8.0
7.5
7.0
6.5
6.0
5.5
5.0
4.5
4.0
3.5
3.0
2.5
2.0
1.5
1.0
f1 (ppm)
H2N
O
Me
N
NC
O
NC
N
N
N
O
N
Me
N
O
Me
O
CN

^{1}H NMR of 6i

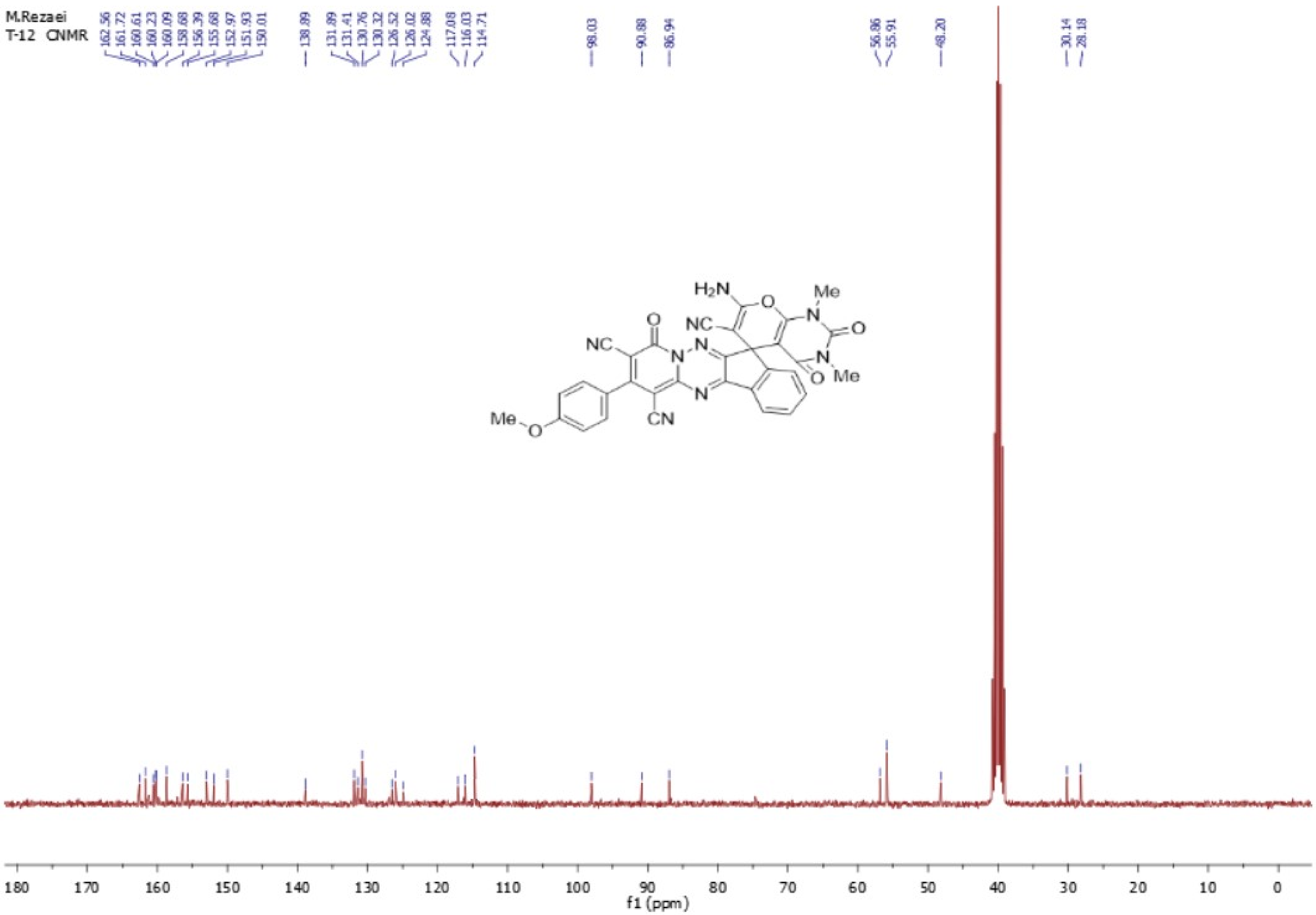
M.Rezaei
T-12 CNMR
162.56
161.72
160.61
160.23
160.09
158.68
156.39
155.68
152.97
151.93
150.01
138.89
131.89
131.41
130.76
130.32
126.52
126.02
124.88
117.08
116.03
114.71
98.03
90.88
86.94
56.86
55.91
48.20
30.14
28.18
H2N
O
Me
NC
N
O
NC
O
N
N
N
N
Me
Me
O
CN
180
170
160
150
140
130
120
110
100
90
80
70
60
50
40
30
20
10
0
f1 (ppm)

^{13}C NMR of 6i

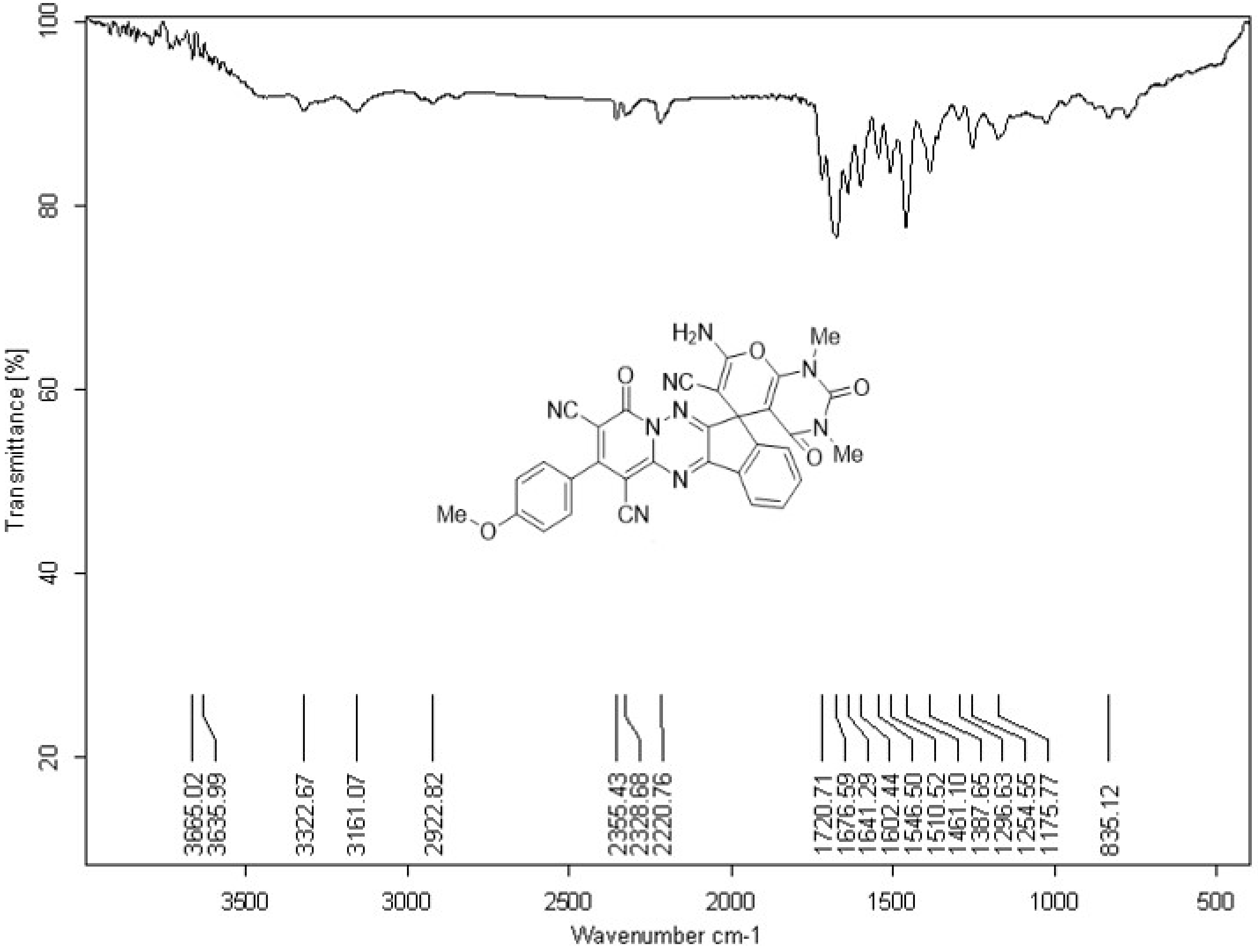
100
80
60
40
20
Transmittance [%]
3665.02
3635.99
3322.67
3161.07
2922.82
2355.43
2328.68
2220.76
1720.71
1676.59
1641.29
1602.44
1546.50
1510.52
1461.10
1387.65
1296.63
1254.55
1175.77
835.12
3500
3000
2500
2000
1500
1000
500
Wavenumber cm-1
H2N
O
Me
N
NC
O
O
NC
N
N
N
O
Me
NC
Me
O
CN

IR of 6i

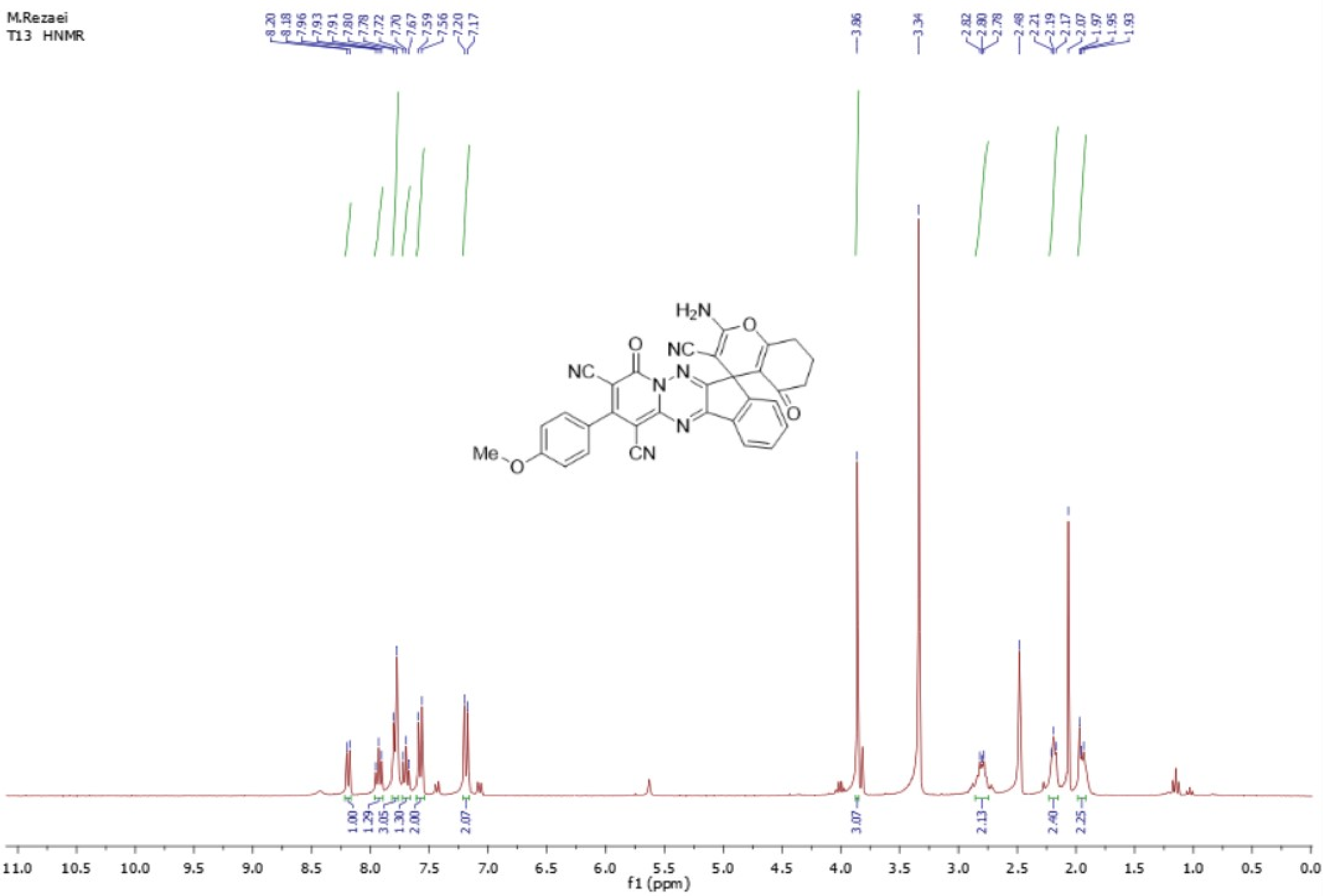
M.Rezaei
T13 HNMR
8.20
8.18
7.96
7.93
7.91
7.80
7.78
7.72
7.70
7.67
7.59
7.56
7.20
7.17
3.86
3.34
2.82
2.80
2.78
2.48
2.21
2.19
2.17
2.07
1.97
1.95
1.93
1.00
1.29
3.05
1.30
2.00
2.07
3.07
2.13
2.40
2.25
f1 (ppm)
H2N
O
NC
NC
N
N
N
O
CN
Me
O

^{1}H NMR of 6j

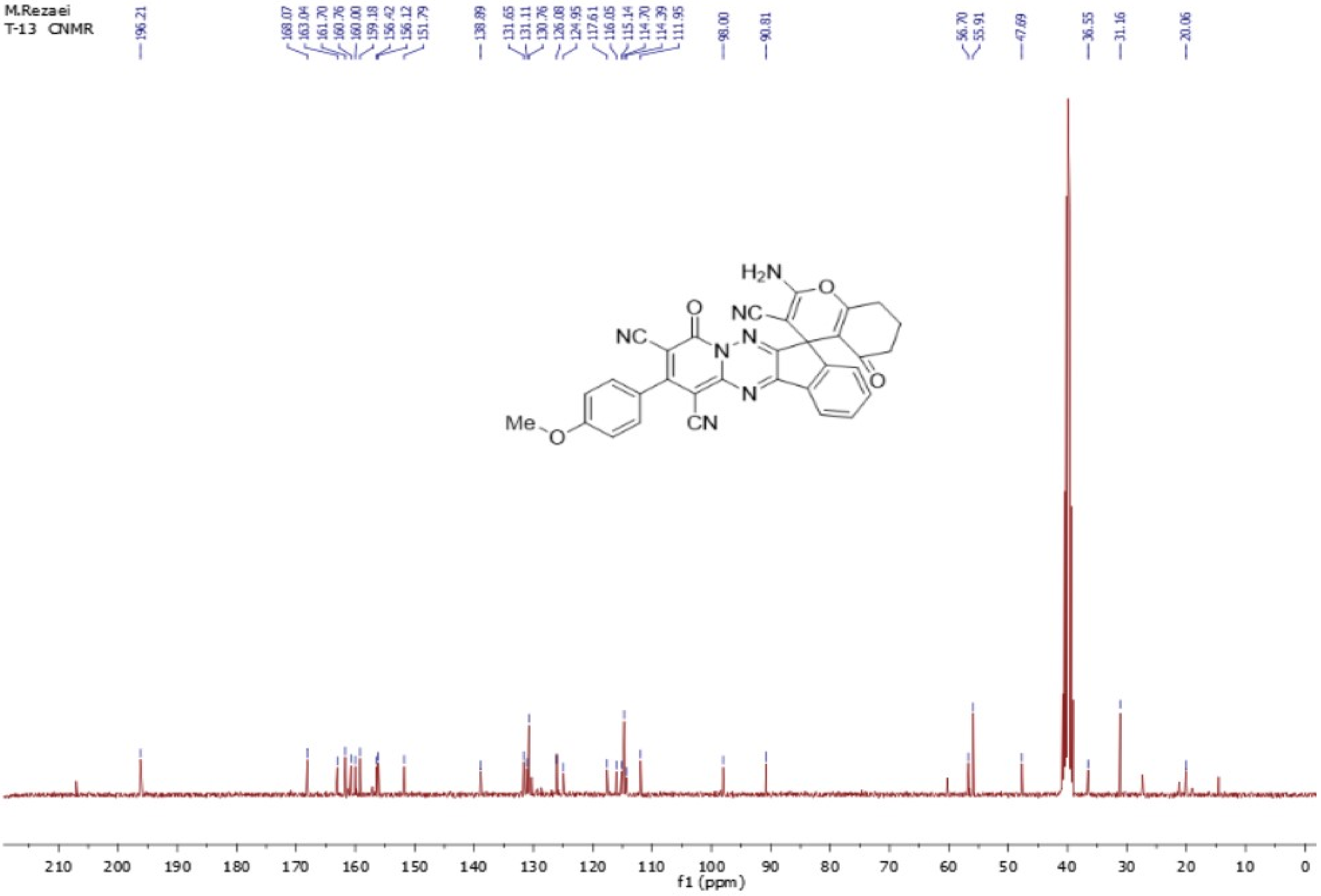
M.Rezaei
T-13 CNMR
196.21
168.07
163.04
161.70
160.76
160.00
159.18
156.42
156.12
151.79
138.89
131.65
131.11
130.76
126.08
124.95
117.61
116.05
115.14
114.70
114.39
111.95
98.00
90.81
56.70
55.91
47.69
36.55
31.16
20.06
f1 (ppm)

^{13}C NMR of 6j

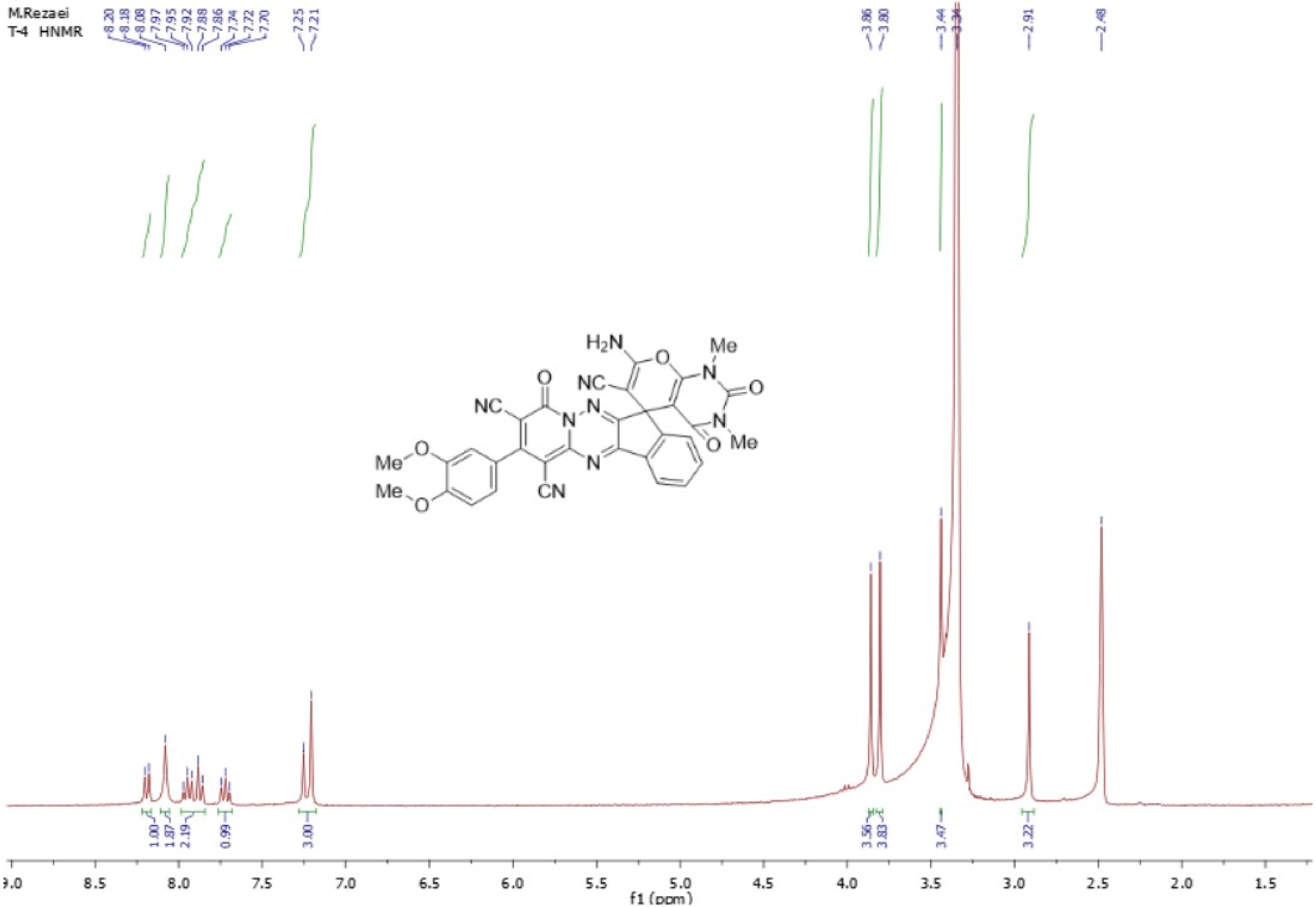
M.Rezaei
T-4 HNMR
8.20
8.18
8.08
7.97
7.95
7.92
7.88
7.86
7.74
7.72
7.70
7.25
7.21
3.86
3.80
3.44
3.34
2.91
2.48
1.00
1.87
2.19
0.99
3.00
3.56
3.83
3.47
3.22
f1 (ppm)
H2N
Me
NC
NC
CN
Me
Me
Me

^{1}H NMR of 6k

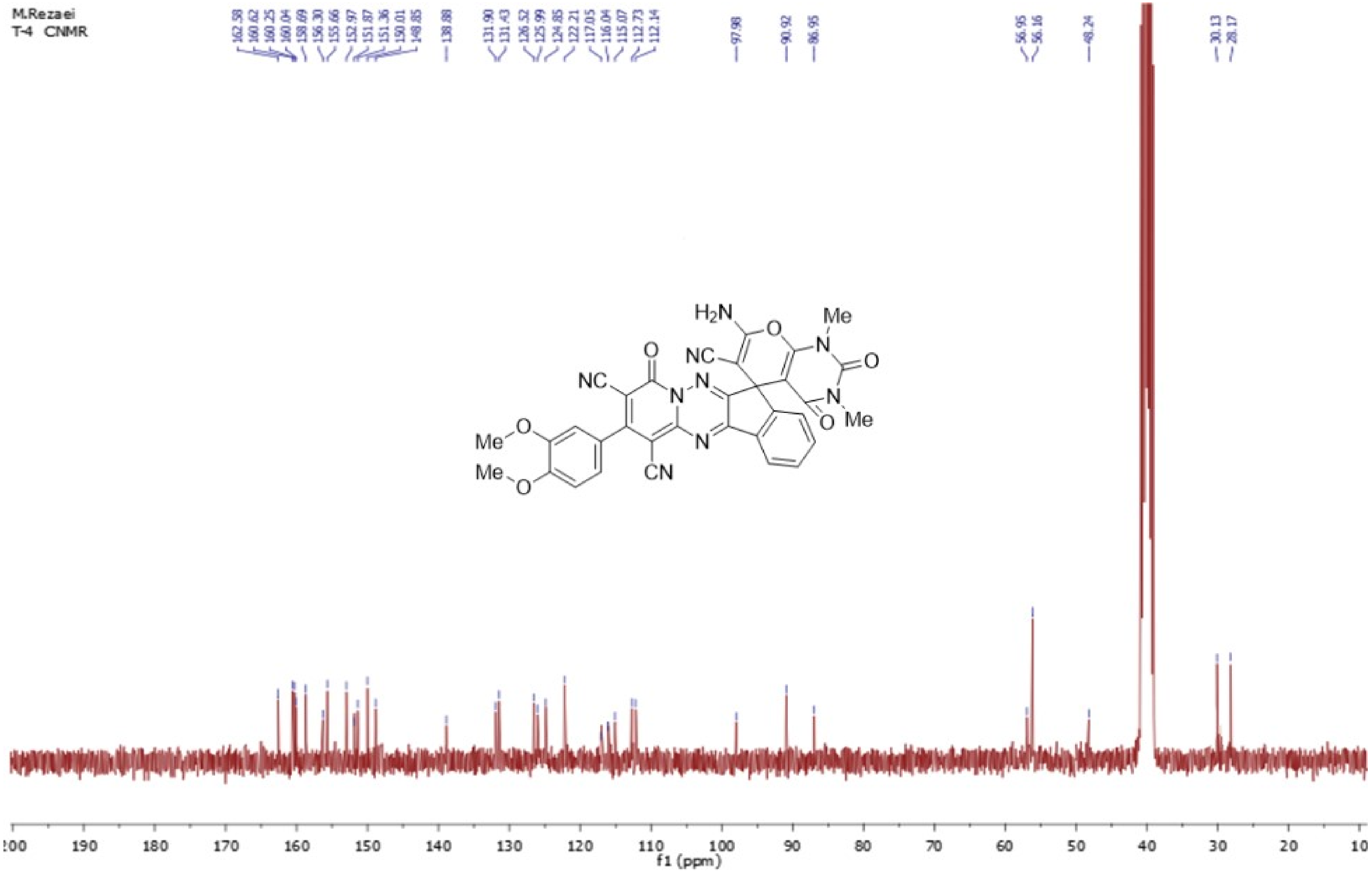
M.Rezaei
T-4 CNMR
162.58
160.62
160.25
160.04
158.69
156.30
155.66
152.97
151.87
151.36
150.01
148.85
138.88
131.90
131.43
126.52
125.99
124.85
122.21
117.05
116.04
115.07
112.73
112.14
97.98
90.92
86.95
56.95
56.16
48.24
30.13
28.17
H2N
O
Me
N
O
NC
O
NC
N
N
N
O
Me
O
N
Me
Me
O
O
N
CN
200 190 180 170 160 150 140 130 120 110 100 90 80 70 60 50 40 30 20 10
f1 (ppm)

^{13}C NMR of 6k

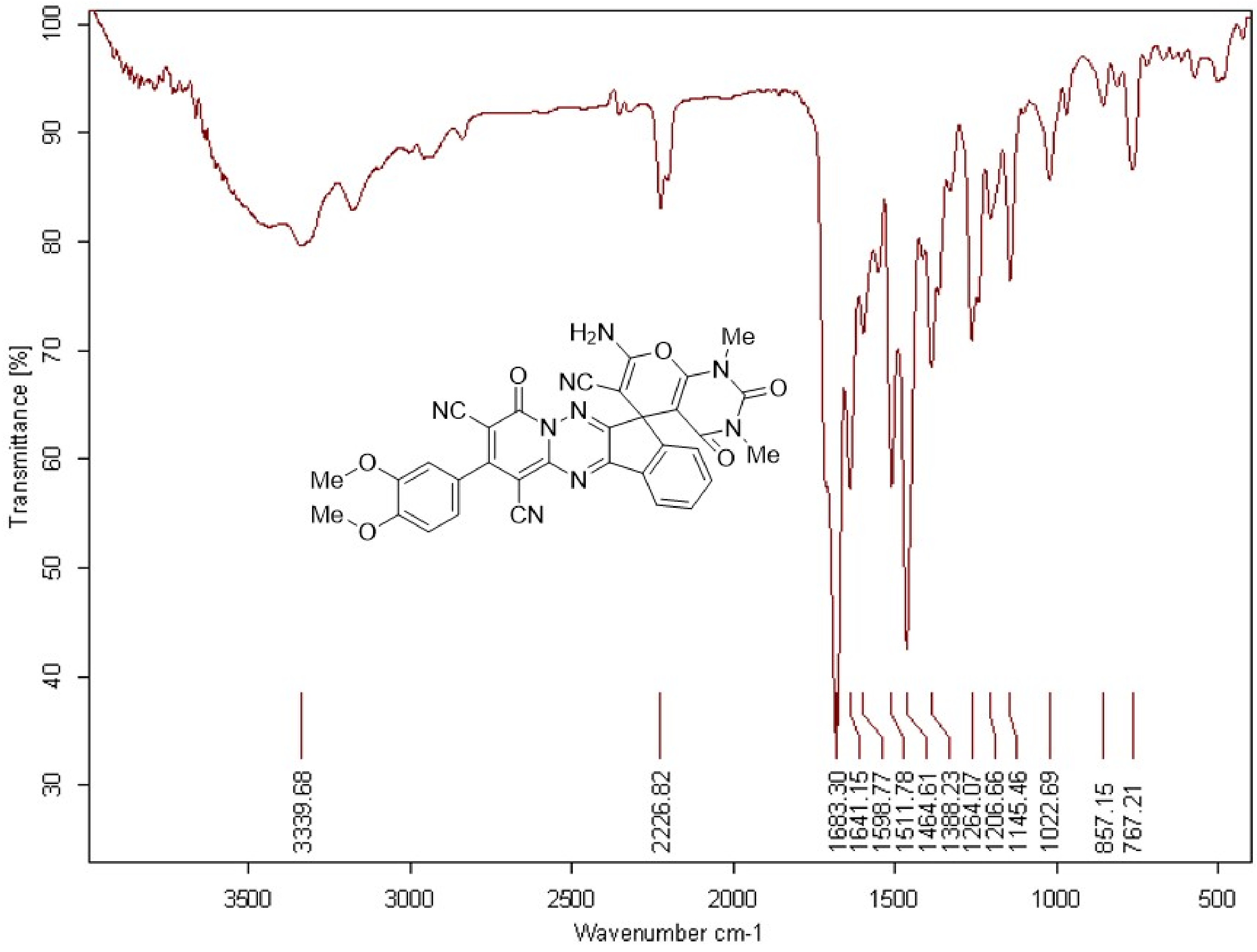

Transmittance [%]
Wavenumber cm-1
3339.68
2226.82
1683.30
1641.15
1598.77
1511.78
1464.61
1388.23
1264.07
1206.66
1145.46
1022.69
857.15
767.21

IR of 6k

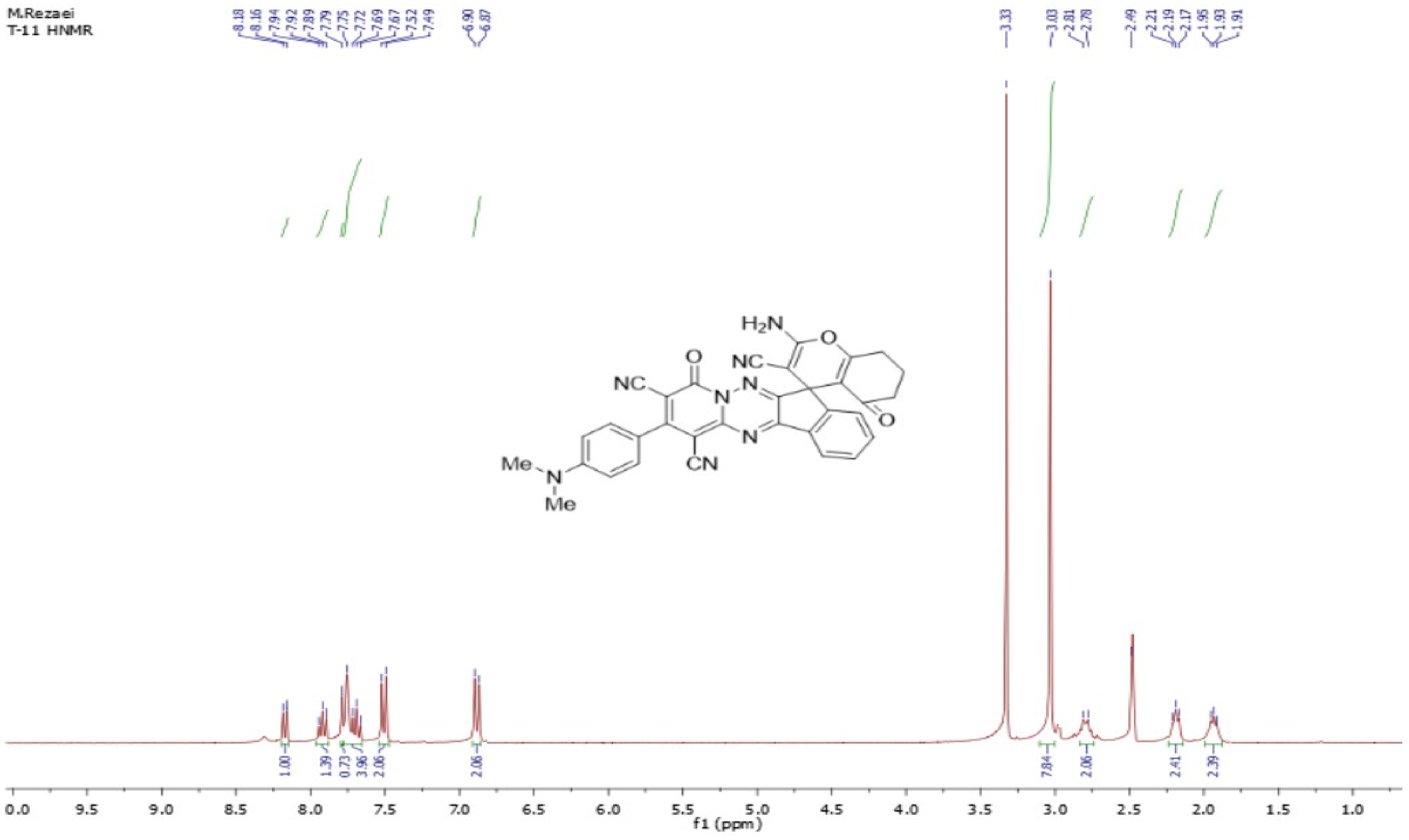

M.Rezaei
T-11 HNMR
8.18
8.16
7.94
7.92
7.89
7.79
7.75
7.72
7.69
7.67
7.52
7.49
6.90
6.87
3.33
3.03
2.81
2.78
2.49
2.21
2.19
2.17
1.95
1.93
1.91
H2N
O
NC
NC
O
N
N
N
O
CN
Me
N
Me
1.00
1.39
0.73
3.96
2.06
2.06
7.84
2.06
2.41
2.39
0.0
9.5
9.0
8.5
8.0
7.5
7.0
6.5
6.0
5.5
5.0
4.5
4.0
3.5
3.0
2.5
2.0
1.5
1.0
f1 (ppm)

^{1}H NMR of 6l

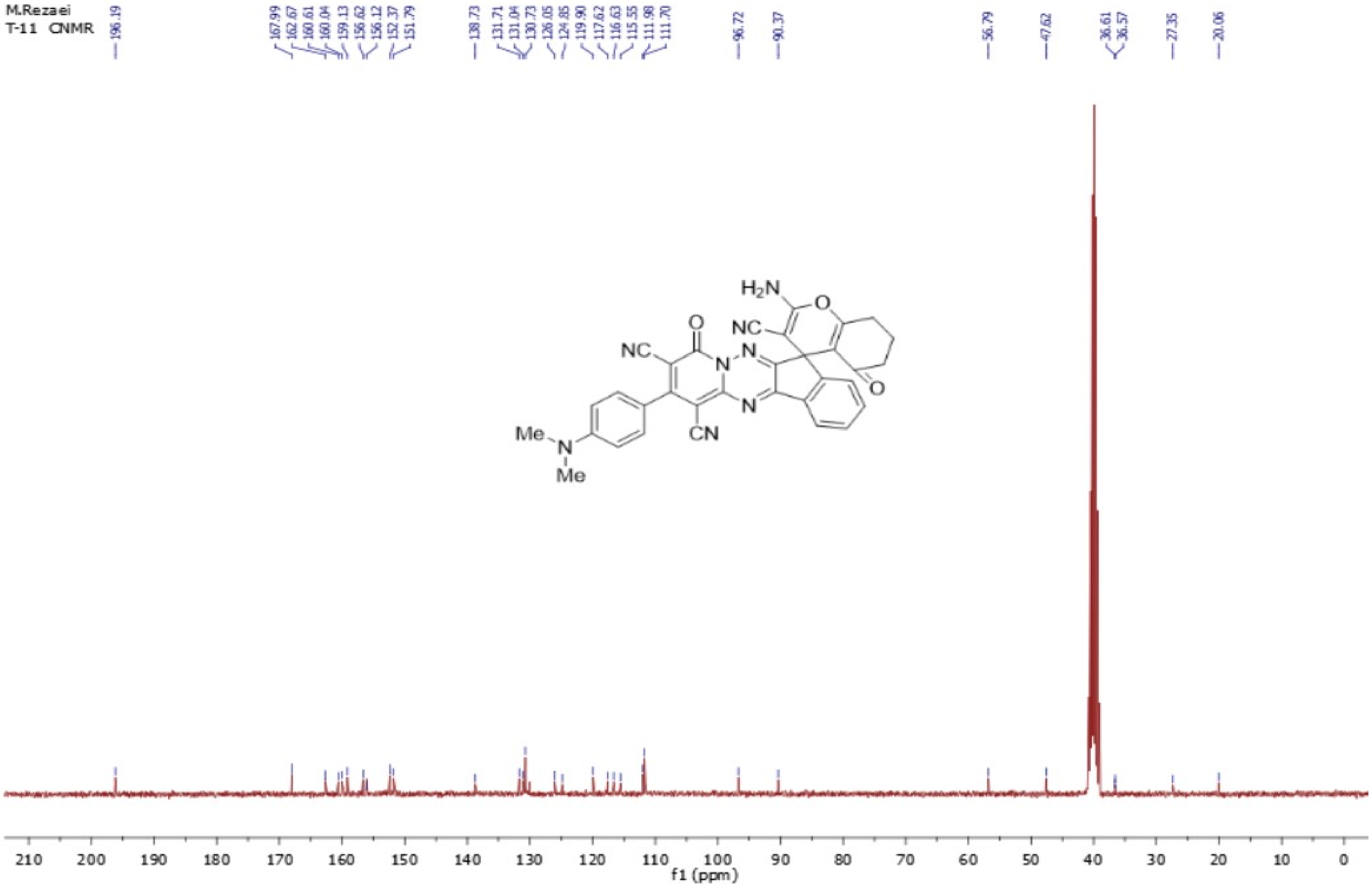
M.Rezaei
T-11 CNMR
196.19
167.99
162.67
160.61
160.04
159.13
156.62
156.12
152.37
151.79
138.73
131.71
131.04
130.73
126.05
124.85
119.90
117.62
116.63
115.55
111.98
111.70
96.72
90.37
56.79
47.62
36.61
36.57
27.35
20.06
H2N
O
NC
NC
O
N
N
N
O
CN
Me
N
Me
210
200
190
180
170
160
150
140
130
120
110
100
90
80
70
60
50
40
30
20
10
0
f1 (ppm)

^{13}C NMR of 6l

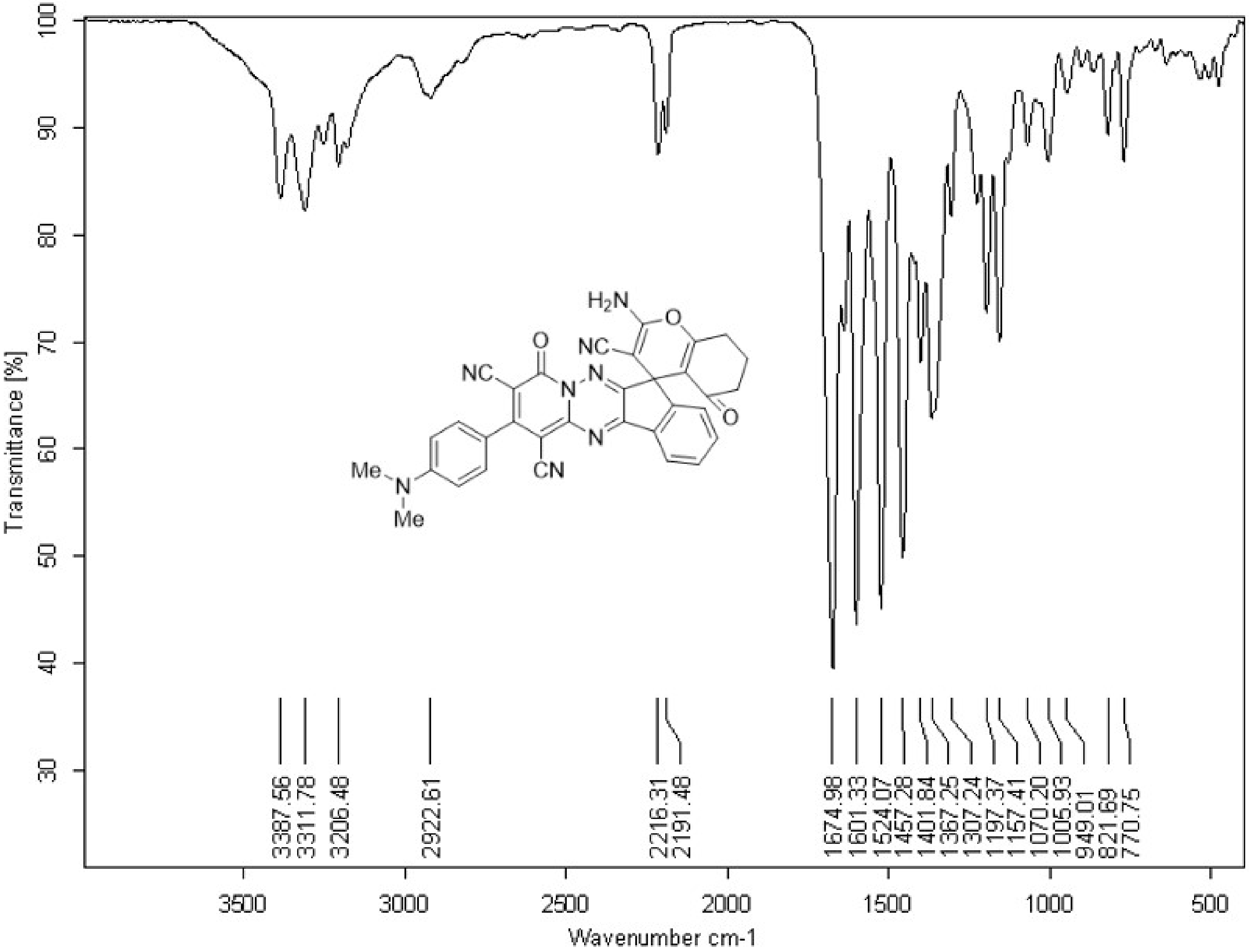
Transmittance [%]
Wavenumber cm-1
100
90
80
70
60
50
40
30
3500
3000
2500
2000
1500
1000
500
3387.56
3311.78
3206.48
2922.61
2216.31
2191.48
1674.98
1601.33
1524.07
1457.28
1401.84
1367.25
1307.24
1197.37
1157.41
1070.20
1005.93
949.01
821.69
770.75
H2N
NC
NC
CN
O
O
O
N
N
N
N
Me
Me
N

IR of 6l

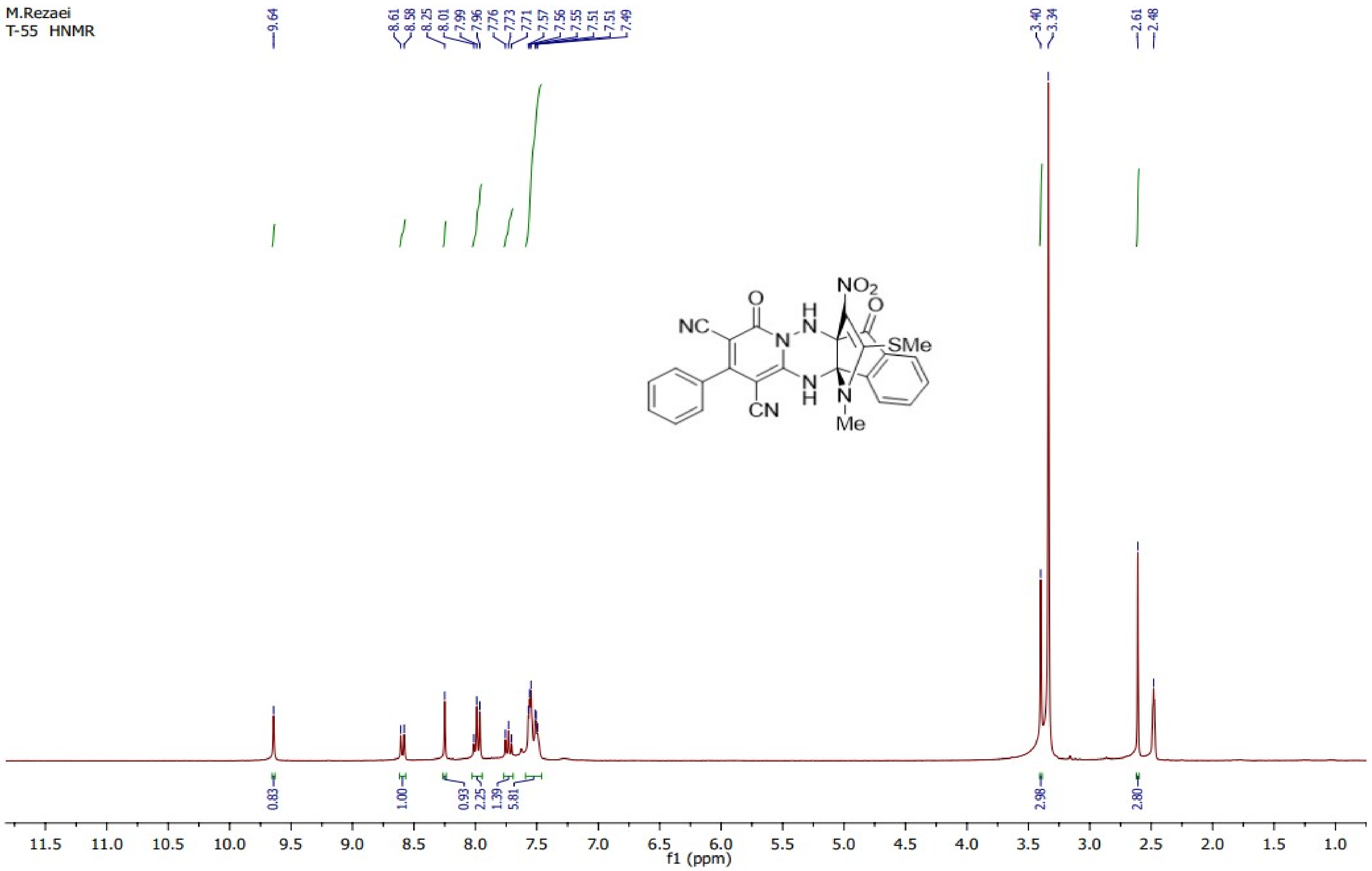
M.Rezaei
T-55 HNMR
9.64
8.61
8.58
8.25
8.01
7.99
7.96
7.76
7.73
7.71
7.57
7.56
7.55
7.51
7.51
7.49
3.40
3.34
2.61
2.48
0.83
1.00
0.93
2.25
1.39
5.81
2.98
2.80
11.5
11.0
10.5
10.0
9.5
9.0
8.5
8.0
7.5
7.0
6.5
6.0
5.5
5.0
4.5
4.0
3.5
3.0
2.5
2.0
1.5
1.0
f1 (ppm)
NC
O
H
N
N
NO2
O
SMe
N
H
CN
N
Me

^{1}H NMR of 8a

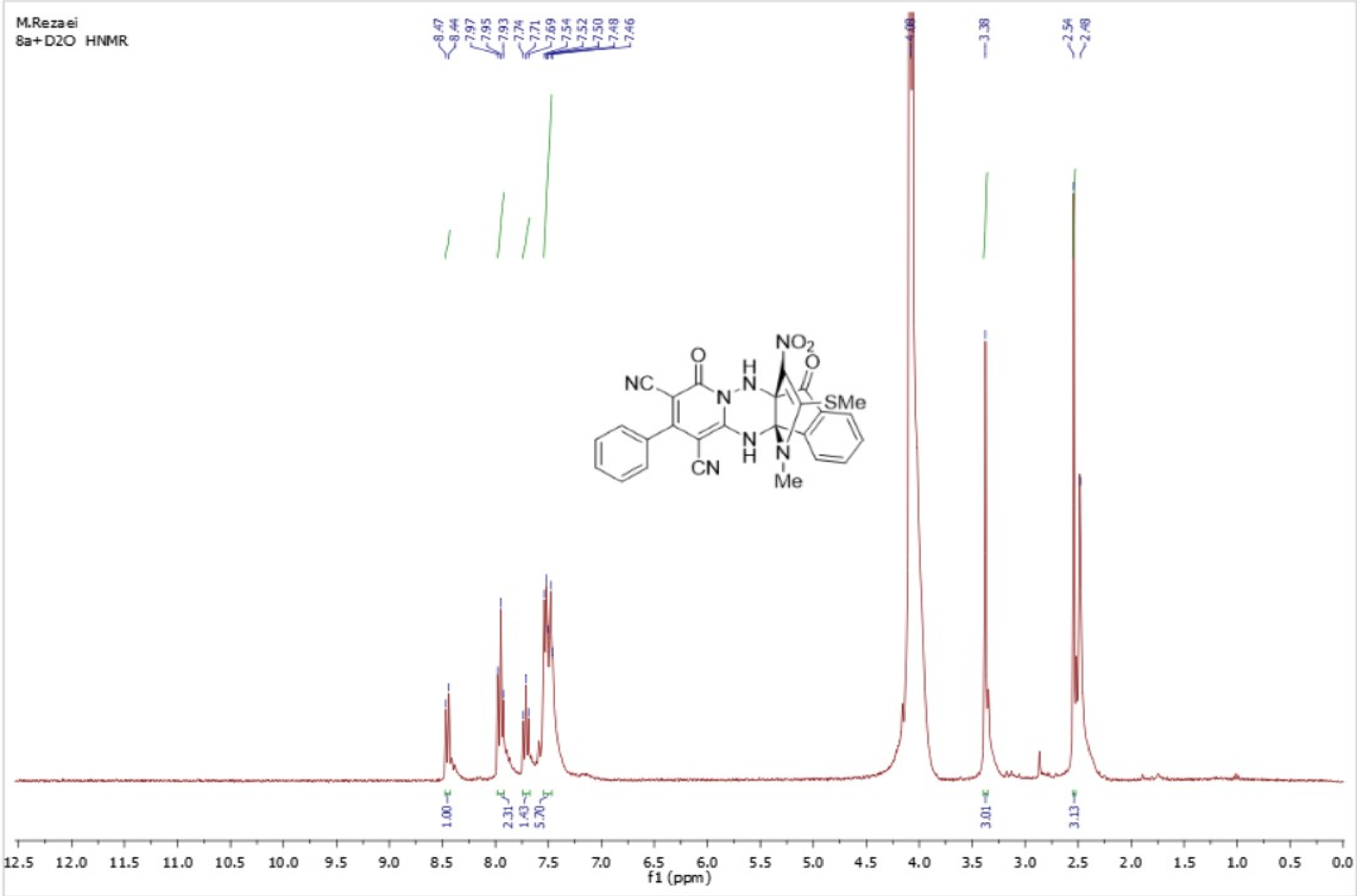

^{1}H NMR of 8a (D$_2$O exchangeable)

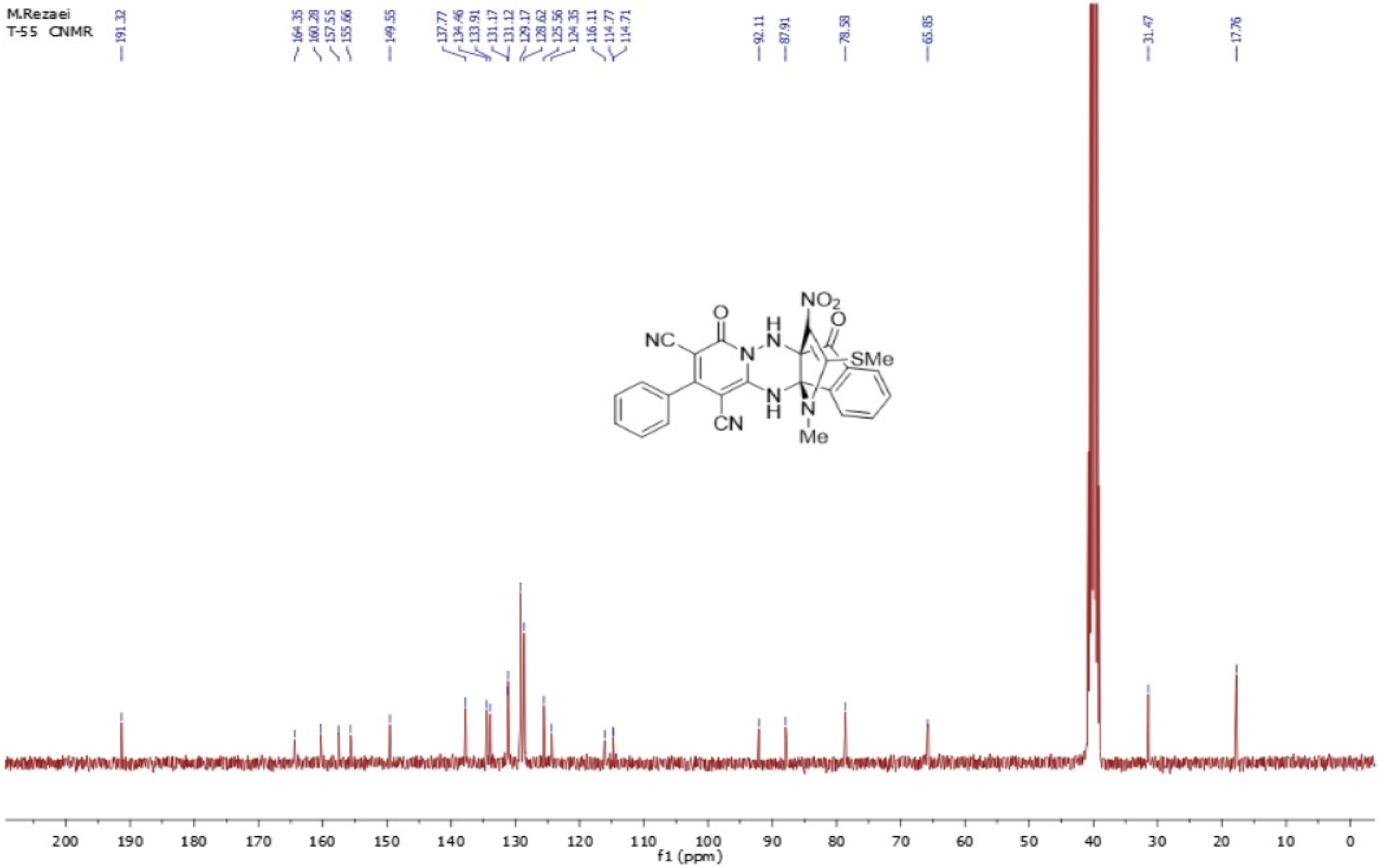
M.Rezaei
T-55 CNMR
191.32
164.35
160.28
157.55
155.66
149.55
137.77
134.46
133.91
131.17
131.12
129.17
128.62
125.56
124.35
116.11
114.77
114.71
92.11
87.91
78.58
65.85
31.47
17.76
NO2
O
NC
SMe
CN
Me
f1 (ppm)

^{13}C NMR of 8a

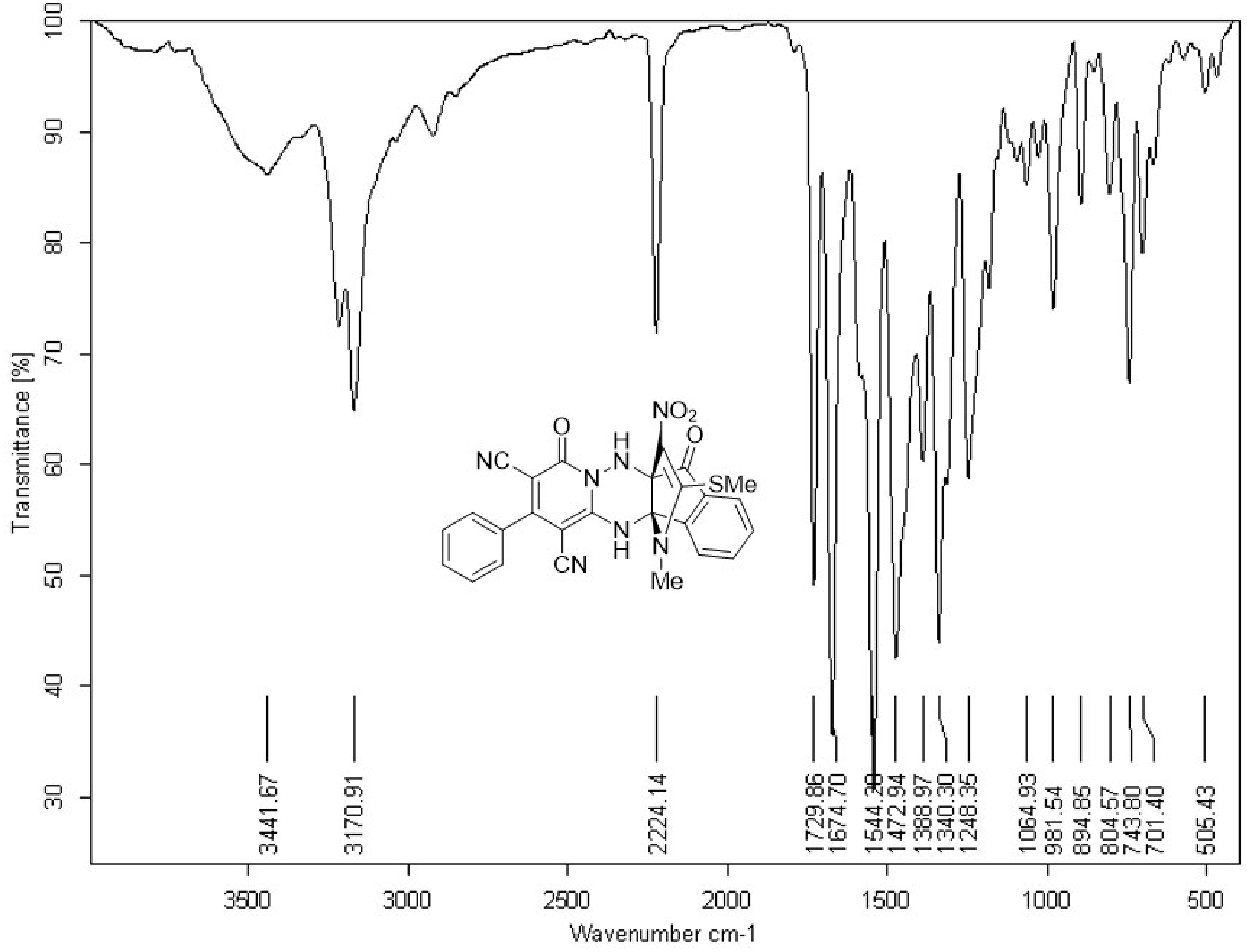
Transmittance [%]
Wavenumber cm-1
3441.67
3170.91
2224.14
1729.86
1674.70
1544.20
1472.94
1388.97
1340.30
1248.35
1064.93
981.54
894.85
804.57
743.80
701.40
505.43
NO2
O
NC
O
H
N
N
SMe
N
H
N
H
CN
Me

IR of 8a

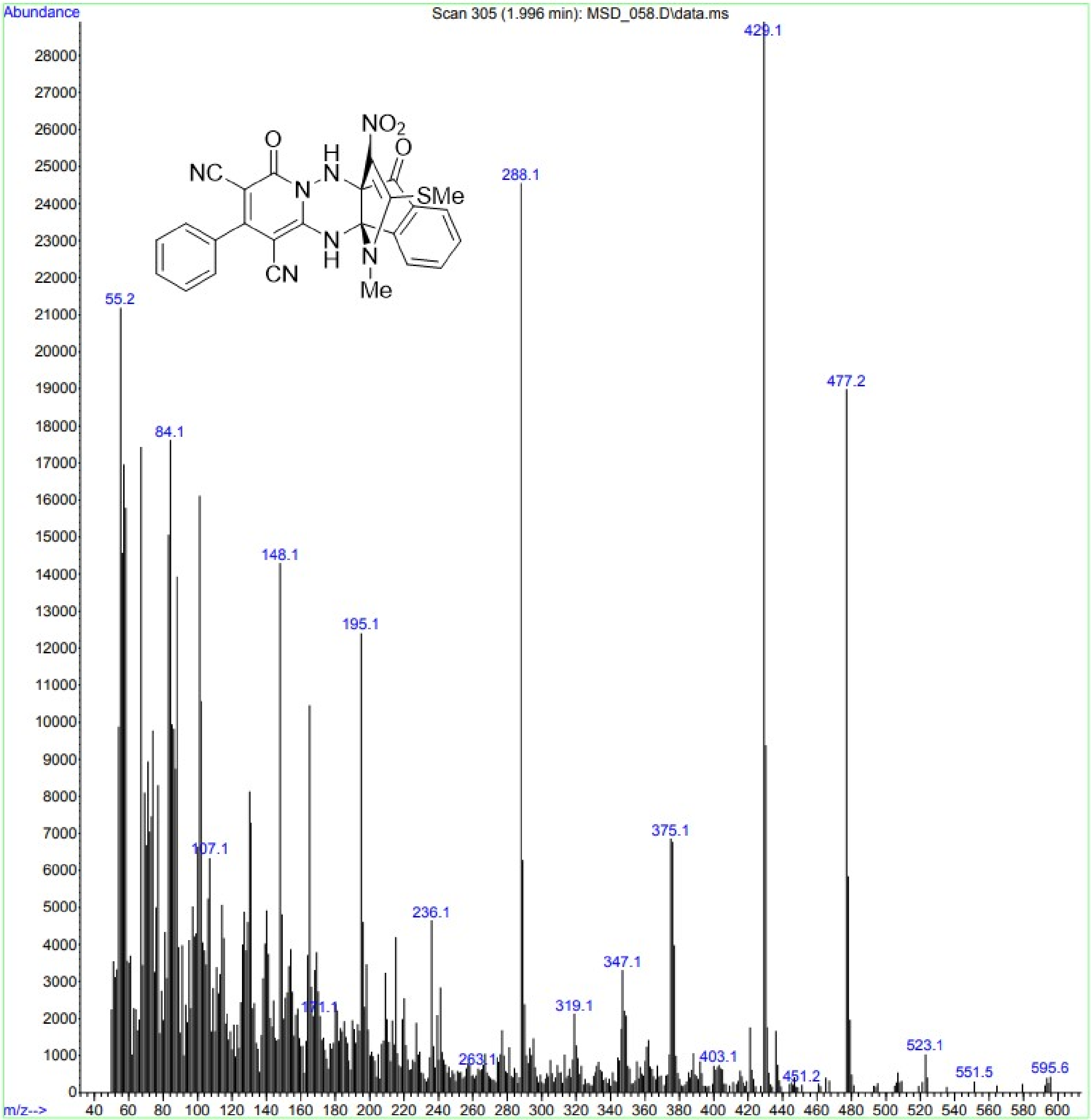
Abundance
Scan 305 (1.996 min): MSD_058.D\data.ms
NC
O
H
N
N
NO2
O
SMe
CN
H
N
N
Me
429.1
288.1
55.2
477.2
84.1
148.1
195.1
107.1
375.1
236.1
347.1
319.1
171.1
263.1
403.1
451.2
523.1
551.5
595.6
m/z-->

MS of 8a

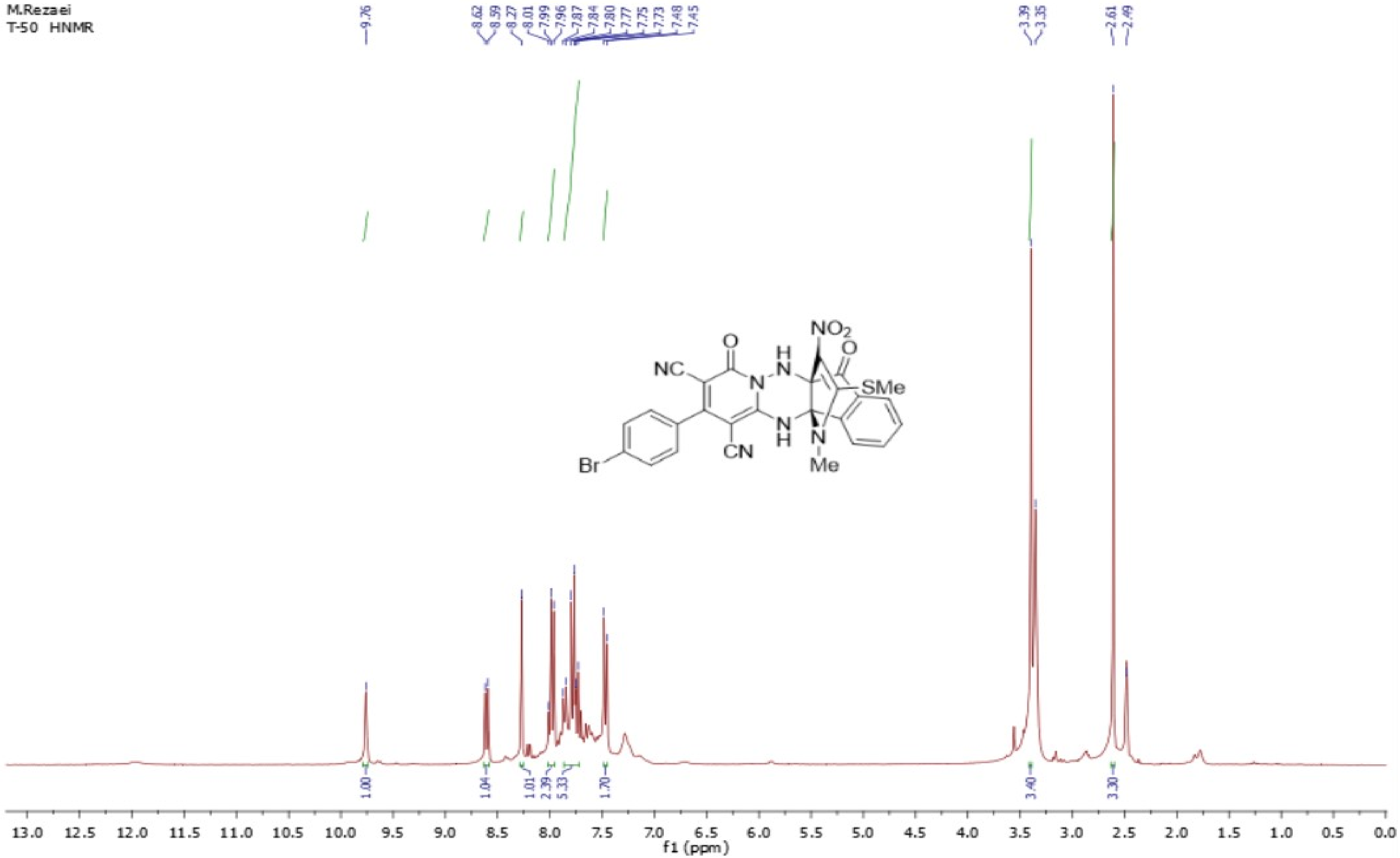
M.Rezaei
T-50 HNMR
9.76
8.62
8.59
8.27
8.01
7.99
7.96
7.87
7.84
7.80
7.77
7.75
7.73
7.48
7.45
3.39
3.35
2.61
2.49
1.00
1.04
1.01
2.39
5.33
1.70
3.40
3.30
f1 (ppm)
NO2
O
H
NC
N
SMe
Br
CN
Me

^{1}**H NMR of 8b**

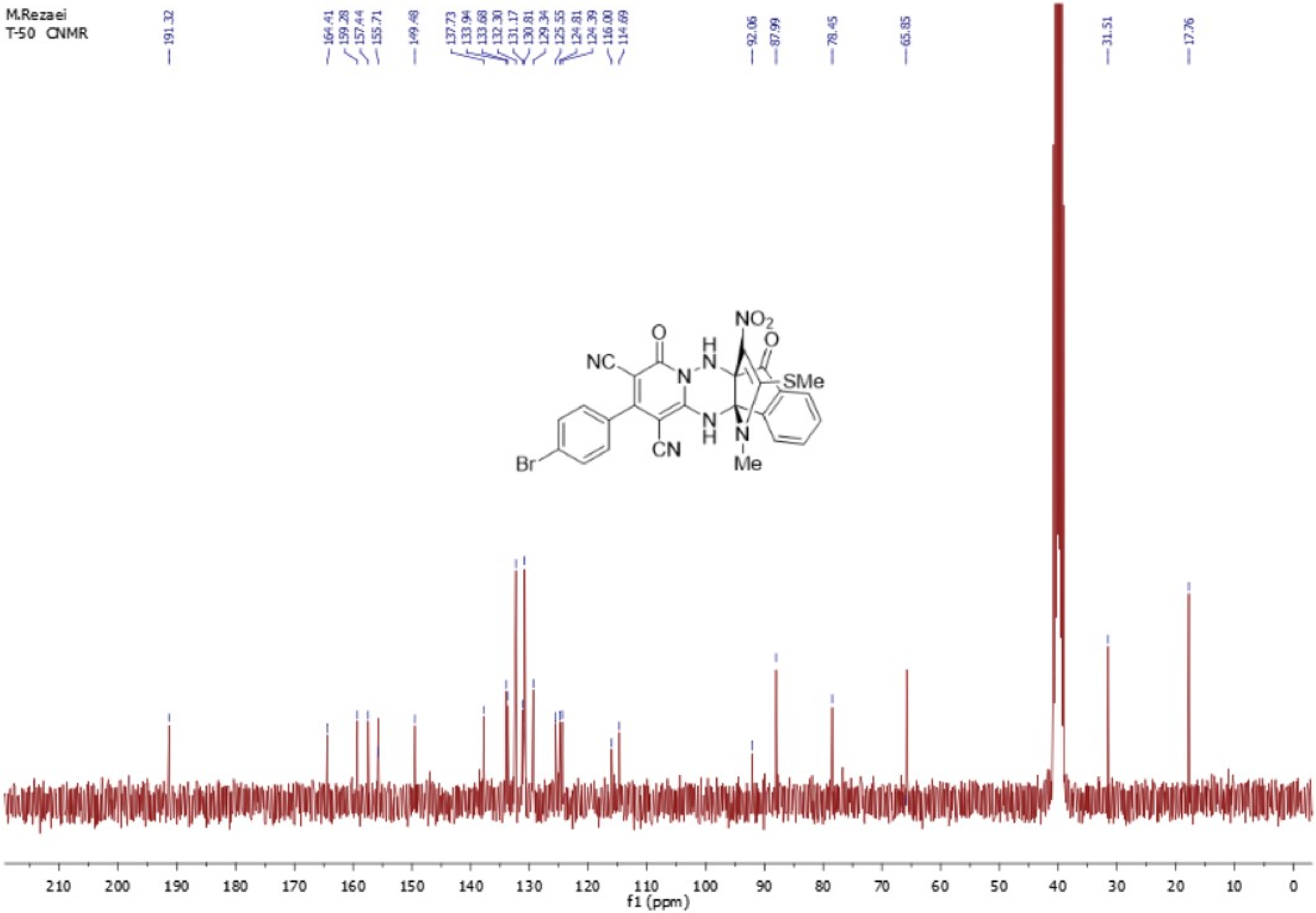
M.Rezaei
T-50 CNMR
191.32
164.41
159.28
157.44
155.71
149.48
137.73
133.94
133.68
132.30
131.17
130.81
129.34
125.55
124.81
124.39
116.00
114.69
92.06
87.99
78.45
65.85
31.51
17.76
NO2
O
NC
SMe
CN
Br
Me
210 200 190 180 170 160 150 140 130 120 110 100 90 80 70 60 50 40 30 20 10 0
f1 (ppm)

^{13}C NMR of 8b

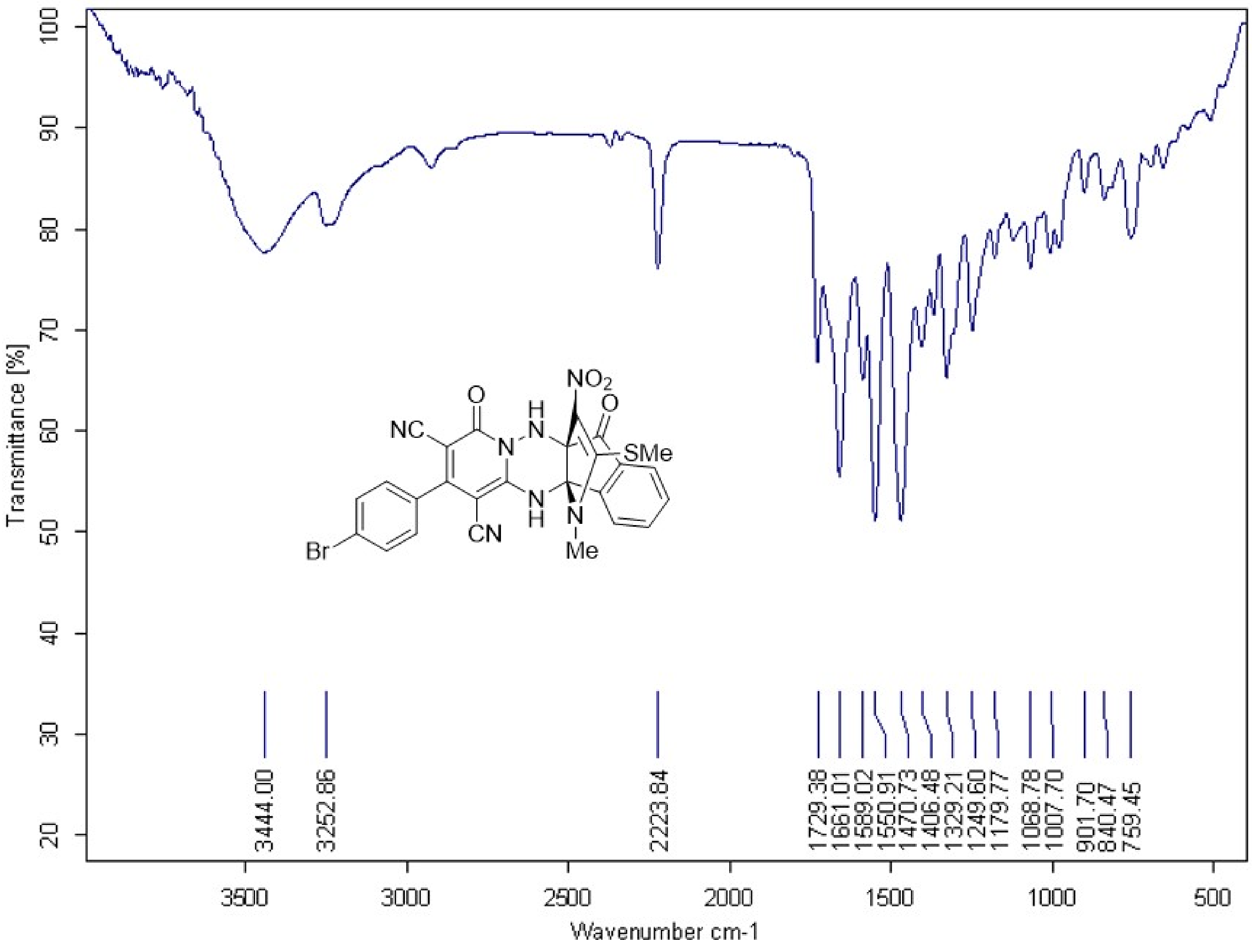

Transmittance [%]
Wavenumber cm-1
100
90
80
70
60
50
40
30
20
3500
3000
2500
2000
1500
1000
500
3444.00
3252.86
2223.84
1729.38
1661.01
1589.02
1550.91
1470.73
1406.48
1329.21
1249.60
1179.77
1068.78
1007.70
901.70
840.47
759.45
NO2
O
O
NC
N
H
SMe
N
H
Br
CN
N
H
N
Me

IR of 8b

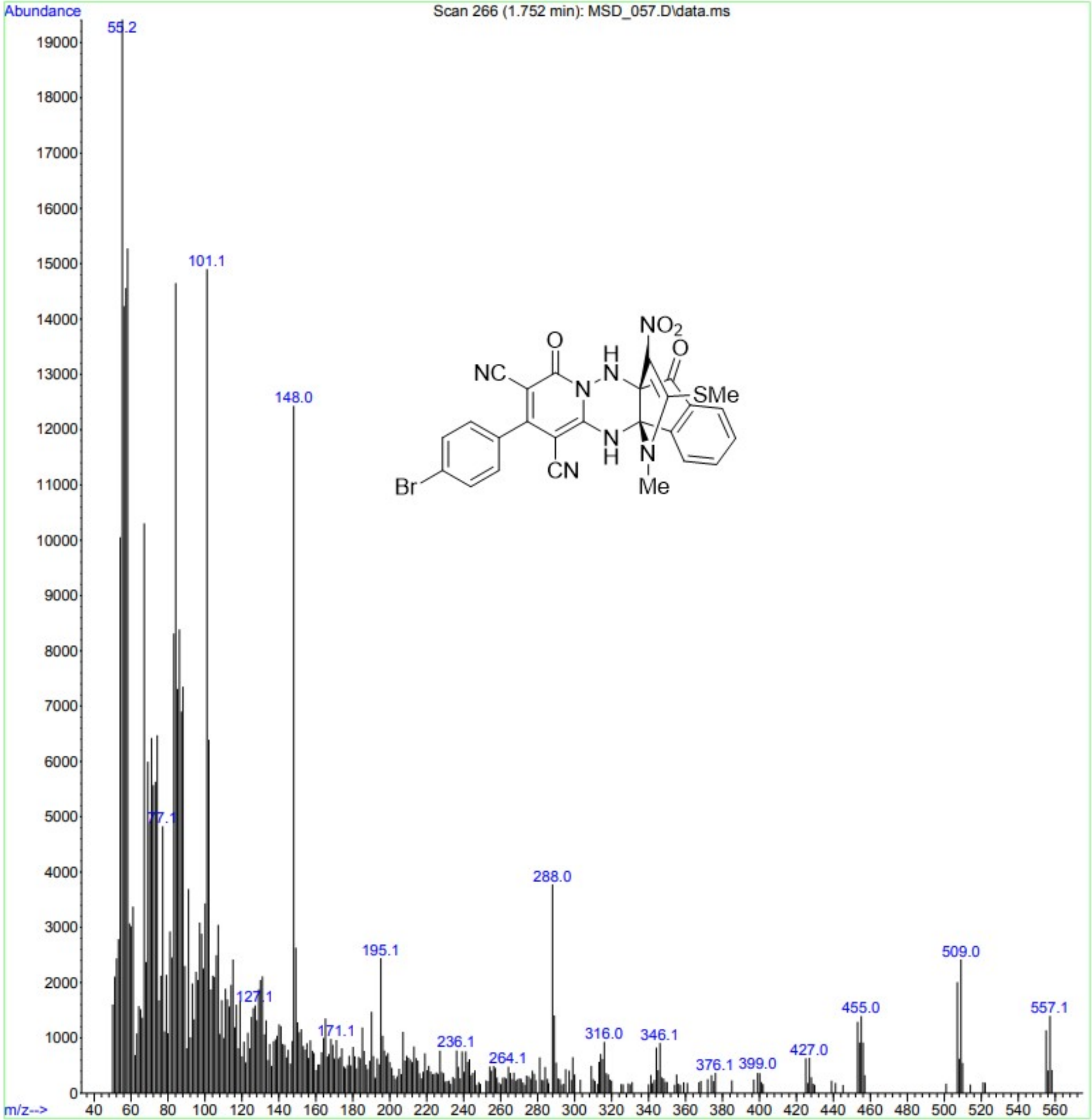
Abundance
Scan 266 (1.752 min): MSD_057.D\data.ms
55.2
101.1
148.0
77.1
127.1
171.1
195.1
236.1
264.1
288.0
316.0
346.1
376.1
399.0
427.0
455.0
509.0
557.1
m/z-->
NC
O
H
N
NO2
O
SMe
CN
Br
Me

MS of 8b

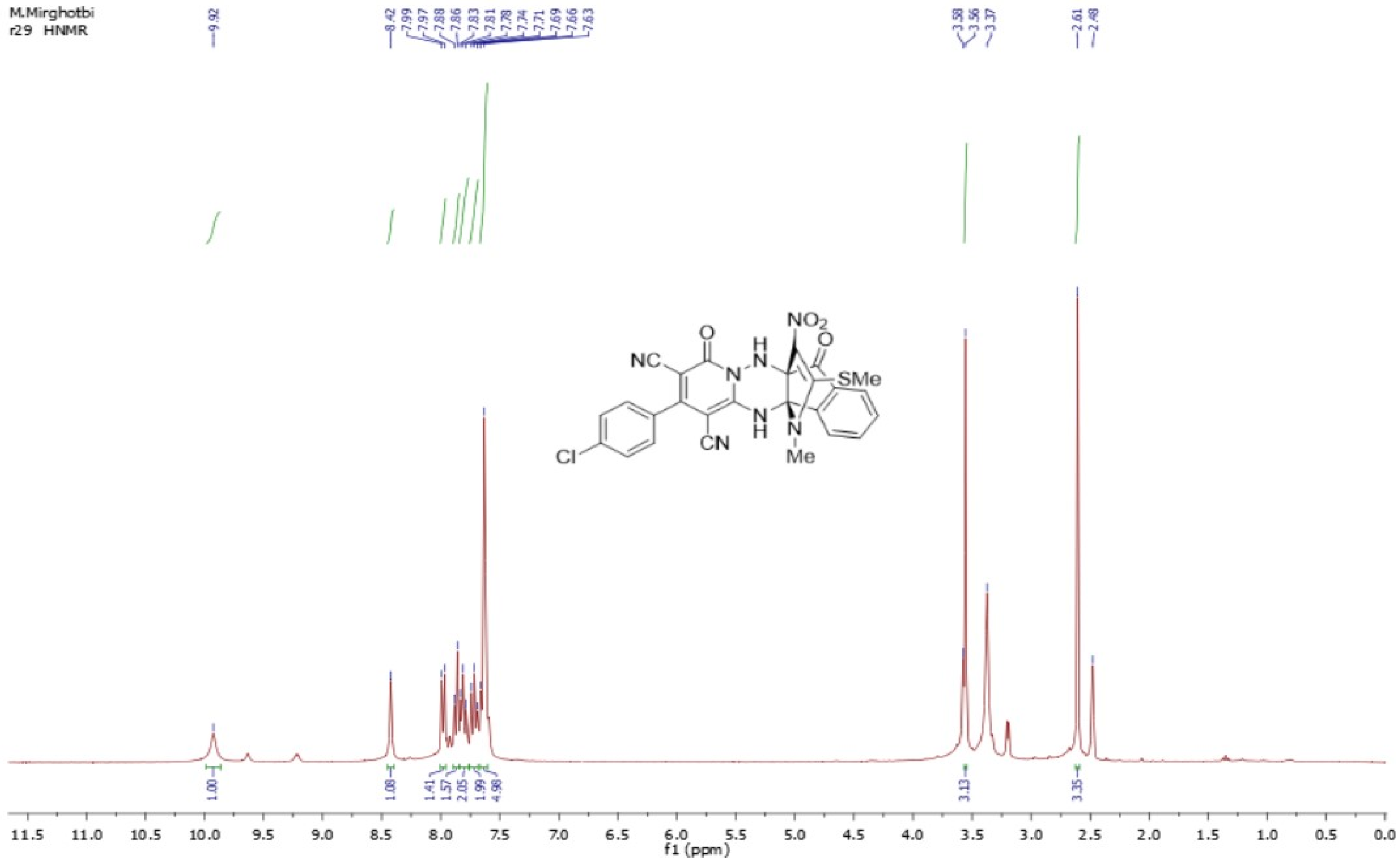

^{1}H NMR of 8c

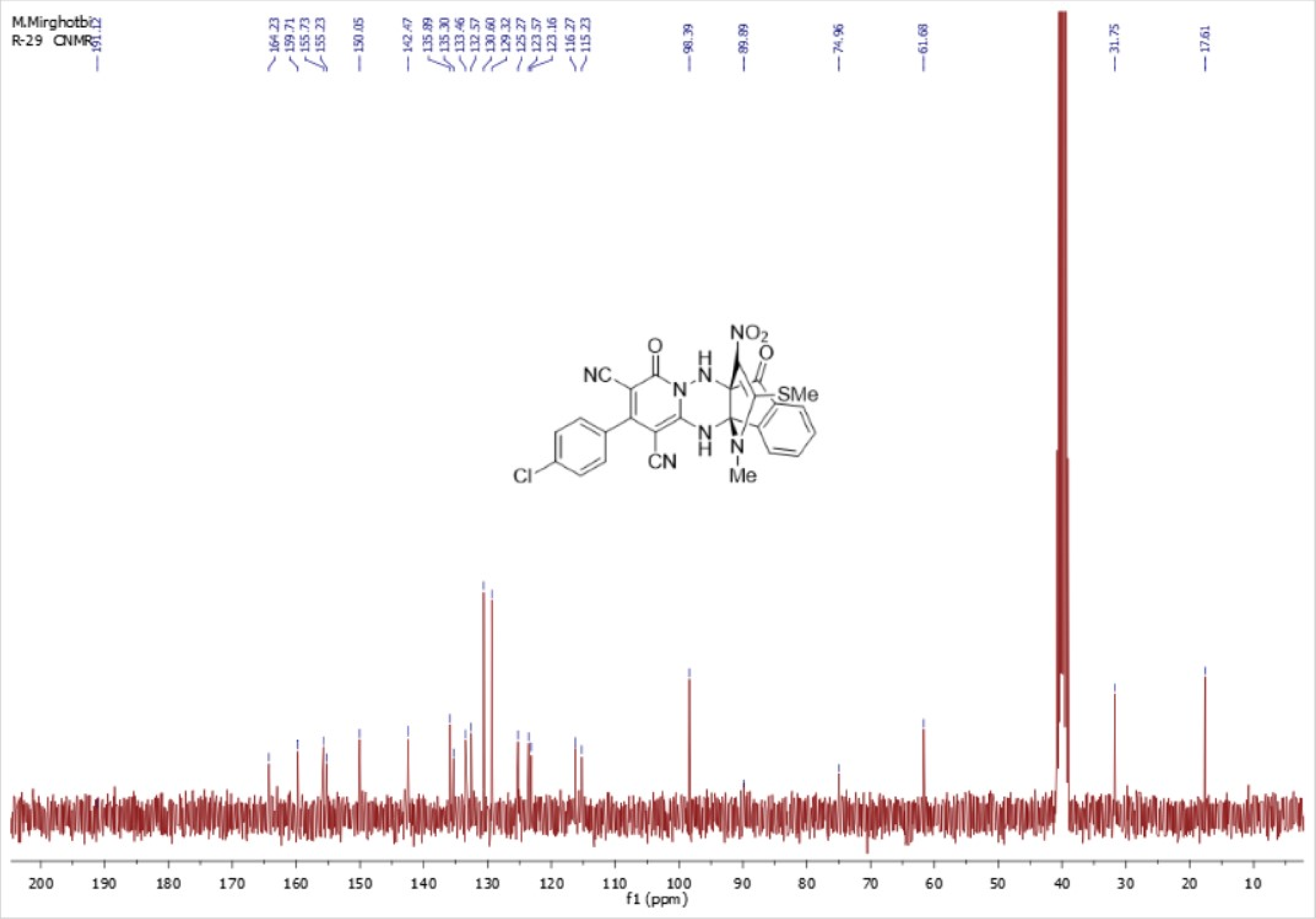
M.Mirghotbi
R-29 CNMR
191.12
164.23
159.71
155.73
155.23
150.05
142.47
135.89
135.30
133.46
132.57
130.60
129.32
125.27
123.57
123.16
116.27
115.23
98.39
89.89
74.96
61.68
31.75
17.61
NC
CN
Cl
NO2
SMe
Me
f1 (ppm)

^{13}**C NMR of 8c**

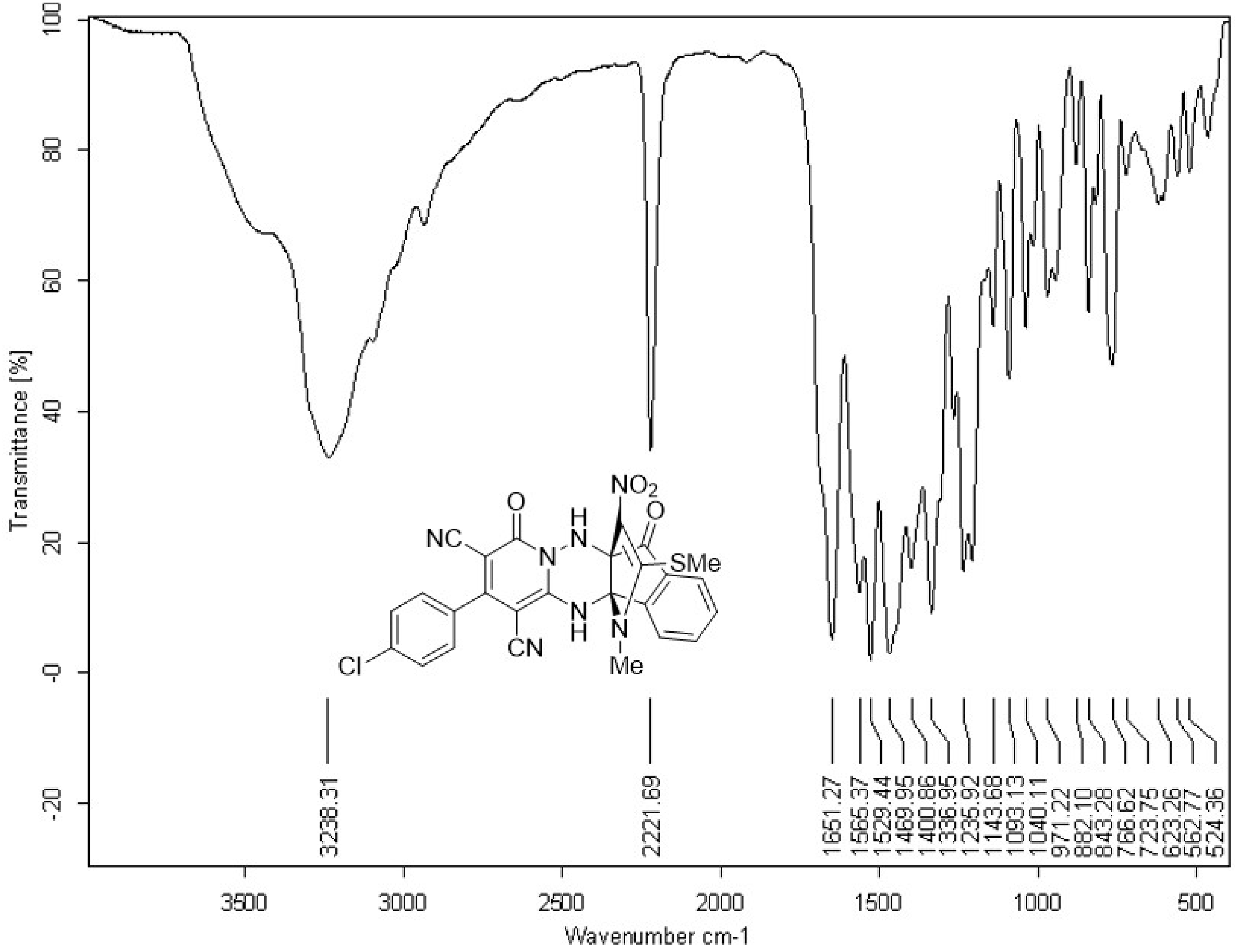
Transmittance [%]
100
80
60
40
20
-0
-20
3500
3000
2500
2000
1500
1000
500
Wavenumber cm-1
3238.31
2221.69
1651.27
1565.37
1529.44
1469.95
1400.86
1336.95
1235.92
1143.68
1093.13
1040.11
971.22
882.10
843.28
766.62
723.75
623.26
562.77
524.36
NO2
O
O
NC
H
N
N
SMe
N
H
N
CN
Cl
Me

IR of 8c

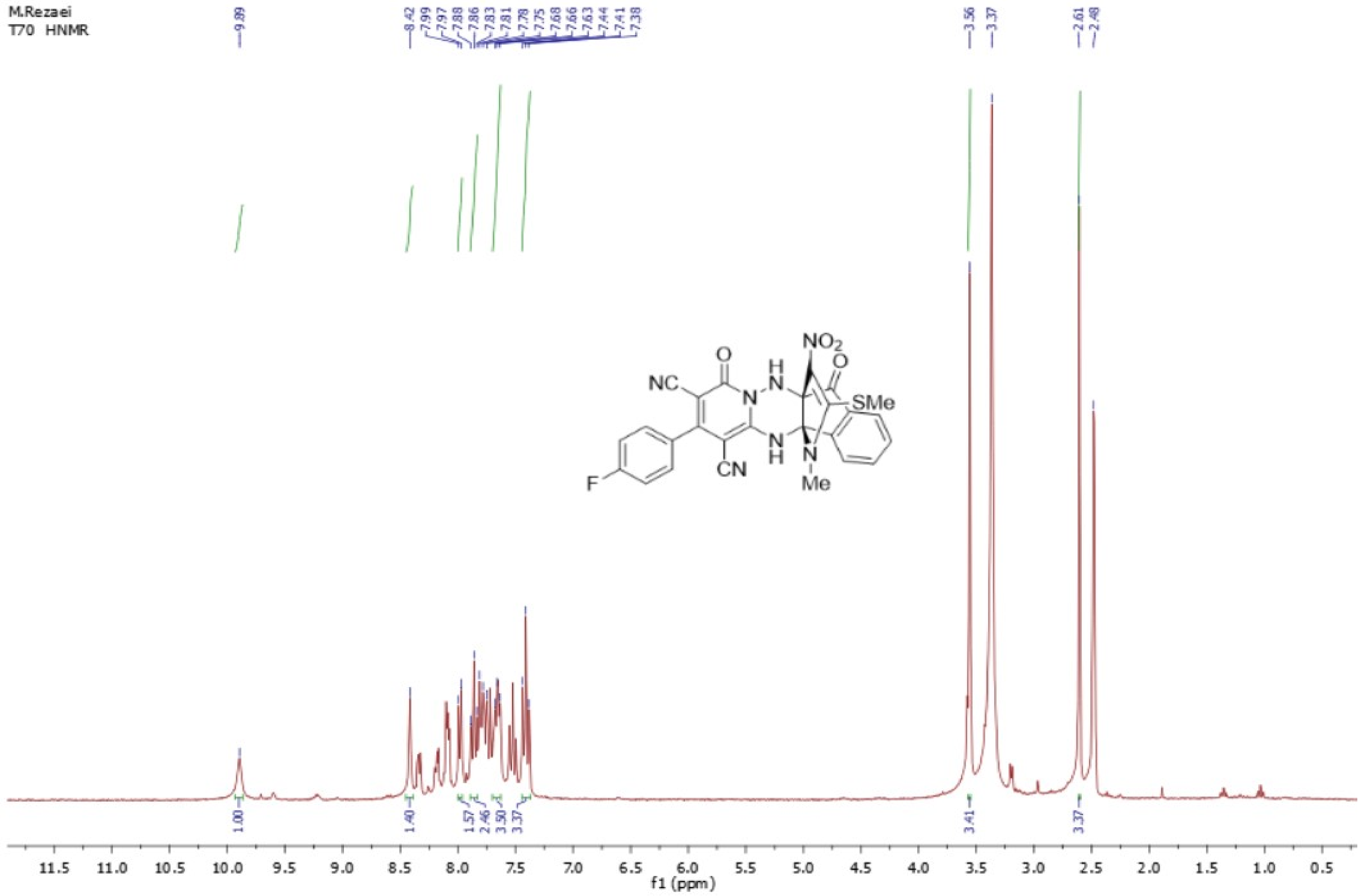
M.Rezaei
T70 HNMR
9.89
8.42
7.99
7.97
7.88
7.86
7.83
7.81
7.78
7.75
7.68
7.66
7.63
7.44
7.41
7.38
3.56
3.37
2.61
2.48
1.00
1.40
1.57
2.46
3.50
3.37
3.41
3.37
f1 (ppm)
NC
CN
F
NO2
SMe
Me

^{1}H NMR of 8d

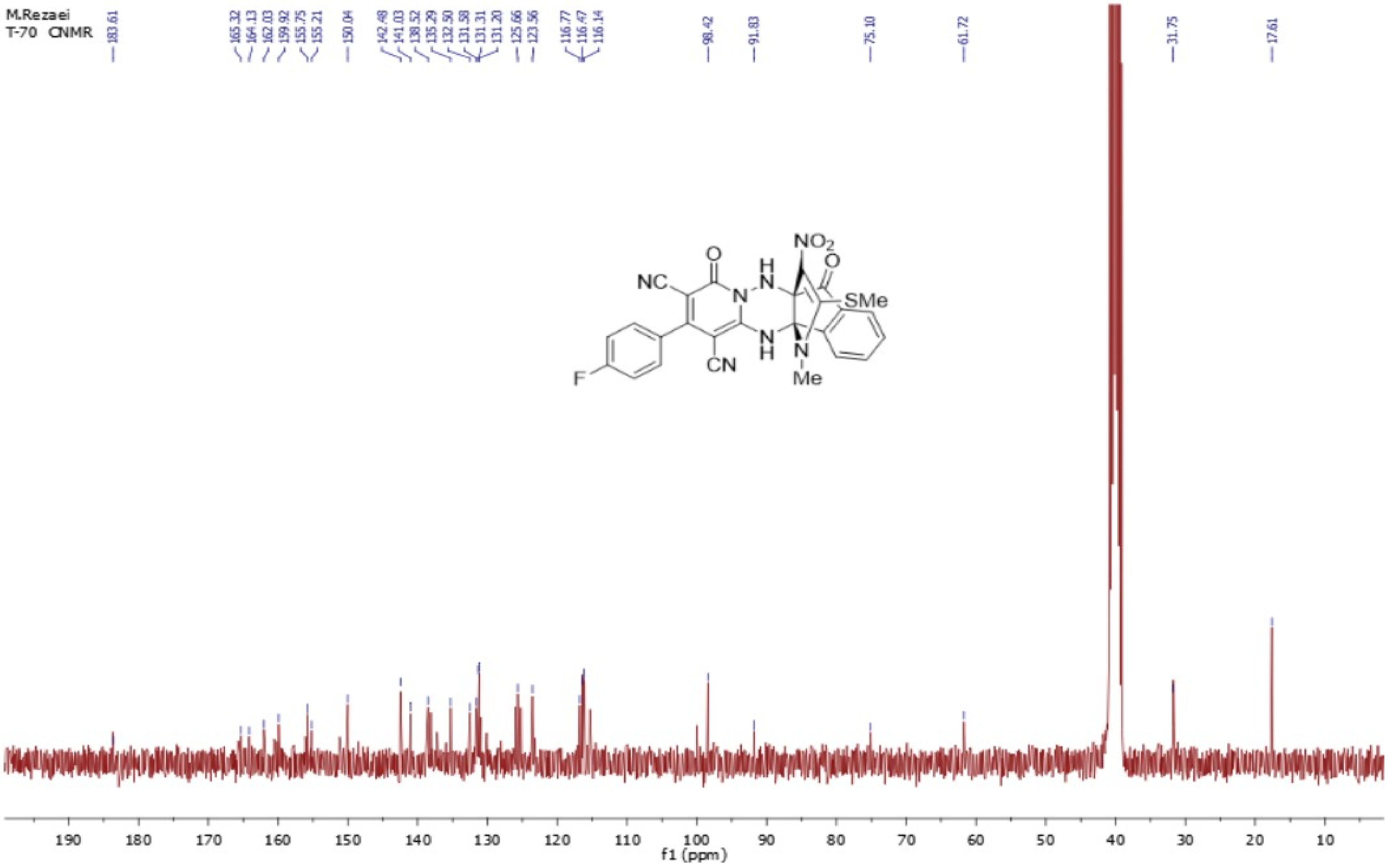
M.Rezaei
T-70 CNMR
183.61
165.32
164.13
162.03
159.92
155.75
155.21
150.04
142.48
141.03
138.52
135.29
132.50
131.58
131.31
131.20
125.66
123.56
116.77
116.47
116.14
98.42
91.83
75.10
61.72
31.75
17.61
NC
O
H
N
N
NO2
O
SMe
F
CN
H
N
Me
190
180
170
160
150
140
130
120
110
100
90
80
70
60
50
40
30
20
10
f1 (ppm)

^{13}C NMR of 8d
